# Supplementary material for: Hagfish genome elucidates vertebrate whole-genome duplication events and their evolutionary consequences
Source: Nat Ecol Evol. 2024 Jan 12;8(3):519–35. doi: 10.1038/s41559-023-02299-z (PMC10927551; doi:10.1038/s41559-023-02299-z)
Supplement: Supplementary file 1 — Supplementary Information. [file 41559_2023_2299_MOESM1_ESM.pdf]

nature ecology & evolution

Article

https://doi.org/10.1038/s41559-023-02299-z

# Hagfish genome elucidates vertebrate whole-genome duplication events and their evolutionary consequences

In the format provided by the authors and unedited

## SUPPLEMENTARY INFORMATION

## INDEX

**1. ASSEMBLY AND ANNOTATION OF THE HAGFISH GENOME ............................................... 2**
1.1) Hagfish genome assembly. ................................................................................................... 2
1.2) Ensembl. ................................................................................................................................ 8
**2. TIME-CALIBRATED PHYLOGENOMICS......................................................................... 14**
2.1) The phylogeny of vertebrates. .............................................................................................. 14
2.2) Calibrations........................................................................................................................... 15
**3. GENE FAMILY EVOLUTION .............................................................................................. 30**
3.1) Gene family gains and losses across vertebrate evolution.................................................. 30
3.2) Analysis of hagfish gene families of interest........................................................................ 31
**4. MACROSYNTENY ANALYSES OF VERTEBRATE GENOMES .............................................. 40**
4.1) Source of genomic data. ...................................................................................................... 40
4.2) Definition of homologous genes........................................................................................... 40
4.3) Definition of homologous chromosomes.............................................................................. 41
4.4) Macrosynteny between chicken and spotted gar. ................................................................ 41
4.5) Inference of vertebrate ancestral chromosomes................................................................... 44
4.6) Resolving how 2R WGD leads to the extant chromosomal phylogeny of gnathostomes. ................. 48
4.7) Phylogenetic analyses of cyclostome and gnathostome genes. ........................................... 54
4.8) Further supra-scaffolding of the 2018-version of the sea lamprey germline genome assembly. ........ 55
4.9) Investigating whether the cyclostome lineage shared gnathostome pre-1R and post-1R fusions. ...... 56
4.10) Gene retention profile-based analyses. .............................................................................. 57
4.11) Estimating the chance of paralogous chromosomal fusions in hagfish evolution. ............................ 63
**5. REGULATORY EVOLUTION................................................................................................. 64**
5.1) Ohnologs................................................................................................................................ 64
5.2) ATAC-seq analysis................................................................................................................. 66
**6. DATING WGD EVENTS AND ASSESSING THEIR MORPHOLOGICAL EFFECT ............... 71**
6.1) Background........................................................................................................................... 71
6.2) Dating Whole Genome Duplications.................................................................................... 72
6.3) Phenotypic disparity. ............................................................................................................ 73
6.4) Discussion.............................................................................................................................. 73
**BIBLIOGRAPHY ........................................................................................................................ 78**

## 1. ASSEMBLY AND ANNOTATION OF THE HAGFISH GENOME

### 1.1) Hagfish genome assembly.

**1.1.1) Genome sequencing.** We extracted high-molecular-weight genomic DNA from the testis of a single, sexually mature individual of the inshore hagfish, *Eptatretus burgeri,* following a standard phenol-chloroform and Proteinase K extraction protocol for high-molecular-weight DNA (Chapter 6, Protocol 1 from Sambrook and Russell[1]) and was used to prepare a total of six paired-end libraries (IDs MM1-MM5 and PE250, with insert size means of 174, 234, 242, 279, 612 and 300 base pairs (bp), respectively) and five long-insert, mate-pair libraries (IDs MM6-MM10, with insert size means of 5, 6, 8.5, 12.5 and 17.5 kilobases (kb)) (Supplementary Table 2). These libraries were sequenced using Illumina HiSeq 2500 (libraries MM1-MM5) and Illumina MiSeq (library PE250) platforms, generating over 753 Gb (Gigabase) of clean data after some filtering (see below), representing ~240X of sequencing coverage (based on an estimated size of 3.12 Gb, see below) (Supplementary Table 2).

**1.1.2) Filtering and correcting raw reads.** Low quality reads from paired-end (MM1-MM5) and mate-pair (MM6-MM10) data were removed with an in-house Perl script. For mate-pair data, reads were shortened 5 bp at the 5' end, and 20 bp at the 3' end. Then, read pairs with more than 30 low-quality bases (Illumina quality score < 7) or more than 20% of unknown bases in at least one read were discarded. For paired-end data, reads were shortened 2 bp at both 5' and 3' end. Read pairs with more than 30 low-quality bases or more than 5% unknown bases in at least one read were discarded. Then, all reads were filtered for duplications and adapter sequences were discarded. Finally, short-insert-size library reads data (MM1-MM5) were corrected with SOAPec v. 2.03 (Luo et al., 2012), with the following parameters: `-k 21 -i 500000000 -g`

`120000000000`. The basic sequencing, filtering and correction stats are shown in Supplementary Tables 2, 3. For the PE250 library with the highest sequencing length (paired end 250 bp) but moderate mean insertion size (300 bp), we applied FLASH software with default parameters to merge a pair of reads into one[2]. As a result, a median length of 385 bp single end library was obtained (Supplementary Table 2).

**1.1.3) Genome size estimation.** We estimated the genome size of *E. burgeri* to be 3.12 Gb using a k-mer distribution of all 17-mers using sequencing data from the five short-insert paired-end libraries (libraries MM1 to MM5; Extended Data Figure 1a, Supplementary Table 4) following previously reported methods[3,4]. Briefly, we calculated the estimated genome size by dividing the total base pairs used for the analysis (filtered reads) by the real sequencing depth (N). N is calculated using the formula $N = (M * L) / (L – k + 1)$ (REF. 3), where L is the average read length, k the k-mer size and M the k-mer coverage at the maximum frequency of the 17-mer distribution (Extended Data Figure 1a). Our size estimation was in line with previous studies[5] and within the range of other hagfish species[6].

**1.1.4) Primary genome assembly and Chicago (Dovetail) scaffolding.** For whole genome sequence assembly, filtered and corrected reads (MM1-MM5, PE250) were firstly assembled into contigs using ABySS[7] (v1.9.055) with a k-mer size of 79. Contigs were assembled into scaffolds with SOAPdenovo2 (REF. 8) (v2.04-r24154, parameters '`-K 41 -d 1 -M 2 -F`') after remapping reads from libraries MM6-MM10 to contigs. Finally, we retrieved the read pairs wherein one end was uniquely mapped to one contig and the other end was located in a gap region, and performed a local assembly for these collected reads to fill the gaps. This gap filling process was performed with GapCloser v1.12-r6, from the SOAPdenovo2 package[8]. To further improve the large-scale structure of this assembly, long-range ligation data from three Chicago libraries

were used to correct and improve the scaffolding of the assembly, using the HiRise pipeline at Dovetail Genomics[9] (Supplementary Table 5). This step increased the contiguity of our assembly by 7-fold, increasing the scaffold N50 from 439 kb to 2.69 Mb, and reducing the number of scaffolds >1kb from 21,115 to 5,465 (Supplementary Table 6). We assembled ~2.60 Gb of the 3.12-Gb estimated size, probably missing hnighly repetitive, difficult-to-sequence microchromosomes[10,11] (see below). This assembly was polished using Pilon[12] (version 1.22) using all data from short-insert libraries and mapped to the genome with Bowtie2 (REF. 13). The resulting corrected genome assembly, version 3.2 in our pipeline, was first publicly opened in Ensembl (http://www.ensembl.org/Eptatretus_burgeri), release 93, in July 2018 (https://www.ensembl.info/2018/07/17/ensembl-93-has-been-released/), with the goal of providing the scientific community with early access to the genome[14].

**1.1.5) Generation of a Hi-C guided hagfish assembly.** We next decided to generate a chromosome-level assembly to enable macrosynteny comparative analysis, crucial to resolve the timing of whole genome duplication (WGD) events in early vertebrate evolution. We generated a Hi-C library from DNA extracted from the testis of a second male individual following a previously described method[15]. We sequenced 2,380 million Hi-C read pairs with an Illumina HiSeq X Ten platform and mapped them to the draft assembly with the HiCUP (version 0.7.2) pipeline[16]. We retained 363 million (15% of the library) valid read pairs, which corresponds to approximately 350X genomic coverage. In a previous study, for a draft human genome assembly with N50 of 437 kb, 240 million retained Hi-C read pairs lead to a new assembly with the clustering error rate below 0.2% and the ordering or orienting error below 2% (REF. 17). Since the *E. burgeri* genome has a similar size than the human genome (3.12 Gb vs 3.1 Gb, respectively) and our Dovetail assembly already reached a N50 of 2.69 Mb, we expected our Hi-C data to be enough to

achieve a high-quality chromosome-level assembly for *E. burgeri*. Since small scaffolds cause serious computing load and are difficult to cluster due to limited Hi-C read pairs mapped to them, we only analyzed 1,635 scaffolds longer than 50 kb, which constitute 99.1% (2,583,606,940/2,608,383,542) of the version 3.2 genome assembly (Dovetail). After loading read pairs mapped to these 1,635 scaffolds into the Hi-C assembly software LACHESIS[17] (compiled on Apr 19, 2019), we tried different parameters in order to optimize the coverage toward the Dovetail assembly. We first used `CLUSTER_N = 18` and `CLUSTER_N = 26`, which represent somatic and germline haploid chromosome number, respectively[18]. Note that although we used a testis sample in order to get germline DNA, it actually represents a mixture of both somatic and germline cells (from the presence of blood vessels and connective tissue of testis). The Hi-C read counts were normalised by a square root vanilla coverage normalisation method[19]. Then it was used as input to draw a heatmap plot in R v3.6.0 via the `ggplot2` package v3.3.5. With this heatmap, we found that one cluster obviously consists of two separate Hi-C contact blocks in case of `CLUSTER_N = 18`. For `CLUSTER_N = 26`, multiple clusters show extensive between-cluster Hi-C contacts. So, we adjusted the parameters to '`CLUSTER_N = 19 CLUSTER_MIN_RE_SITES = 200 CLUSTER_MAX_LINK_DENSITY = 3.6 CLUSTER_NONINFORMATIVE_RATIO = 6 ORDER_MIN_N_RES_IN_TRUNK = 100 ORDER_MIN_N_RES_IN_SHREDS = 50`' and assigned 96.2% (1573/1635) scaffolds longer than 50 kb into 19 clusters (preliminary Hi-C assembly; Supplementary Figure 1).

Next, by integrating different strategies to identify chromosomal rearrangements or misassemblies[17,20,21], and to polish likely Dovetail missassemblies (Supplementary Figure 1), we divided each scaffold into 200 kb sized segments and treated scaffolds smaller than 200 kb as a single segment, and then developed a hybrid strategy to detect and correct assembly errors based

on three types of Hi-C contact information (see Supplementary Figure 2 for an example). First, we examined how a segment interacted with its original cluster and other clusters. To do so, we calculated the read pair count between any segment pair. For a segment of interest, we calculated the cluster level interaction intensity as the median of this segment relative to all segments harboured by one cluster of interest. For any 200 kb-segment, if its highest Hi-C contact occurs in another cluster not associated with its scaffold (Supplementary Figure 2), the corresponding scaffold could be misassembled. Second, for each candidate misassembled scaffold, the read pair densities between this scaffold and two clusters (only the cluster the scaffold belongs to and the other cluster that show highest Hi-C contact with segments of the scaffold) would be partitioned where different segments interact with two cluster differentially (Supplementary Figure 2). Third, for each candidate misassembled scaffold, the read pair densities within this scaffold would be partitioned too where more than one Hi-C interaction block would emerge (Supplementary Figure 2). With these three lines of information, we identified 280 scaffolds associated with 364 misassembly boundaries since a single scaffold could harbour two or more misjoinings (Supplementary Table 7; Supplementary File 1). Given the boundary coordinates, we split these scaffolds into smaller scaffolds and performed a second round of Hi-C based assembly with the same parameters.

Given Hi-C interaction heatmaps, we examined orders of scaffolds within a cluster and performed adjustment in 10 clusters, including cluster1, cluster2, cluster3, cluster5, cluster6, cluster7, cluster8, cluster9, cluster10 and cluster11. We assigned 98.7% (1873/1999) of all corrected scaffolds longer than 50 kb to 19 clusters (main Figure 1d; Supplementary Tables 8, 9). These scaffolds constitute 98.4% (2,578,071,452/2,608,383,542) of the Dovetail hagfish draft assembly in size and include 99.1% (16363/16513) of the annotated protein coding genes.

Accordingly, we translated the gene coordinates from the Dovetail to the Hi-C assembly and excluded 41 genes that overlapped with boundaries of misjoins (Supplementary Table 10). This corrected assembly (v4.0 in our pipeline) served as the final assembly used in the main text.

**1.1.6) Investigation on hagfish Hi-C cluster 19.** The Hi-C contact cluster 19 is the smallest cluster in size (~32 Mb; Figure 1d, Supplementary Table 9). *E. burgeri* germline haploid number is n=26, but during development 8 pairs of germline-specific chromosomes are eliminated through chromosome elimination, making the haploid number of somatic tissues n=18 (REF. 10). These germline-specific chromosomes are highly enriched in repetitive sequences, making them difficult to assemble with short-read sequencing technologies like the one used in this study. We calculated the read coverage across Hi-C clusters by randomly selecting raw data from the genome sequencing (MM-1 lane 1, MM-2 lane 2, MM-5 lane 4 and MM-5 lane 4_2, Supplementary Table 3) and measuring the read depth of uniquely mapped reads to each cluster of the Hi-C assembly. DNA sequencing reads were mapped to the hagfish Hi-C genome assembly using BWA[22], version 0.7.2-r351. Only reads with mapping quality exceeding 30 were reported. Base level read coverage was calculated with Bedtools genomecov tool[23], version 2.25.0. We found that all 19 clusters have a comparable read depth (Supplementary Table 9). However, compared with the remaining clusters, Hi-C cluster 19 depicts the most uneven distribution of read coverage along its base pairs (Supplementary Figure 3), probably because it contains the highest proportion of N's in its sequence (73% v.s. 42-45%; Supplementary Table 9), indicating that it might consist mostly of difficult-to-assemble, highly repetitive sequences, especially when using only short read data. In conclusion, Hi-C cluster 19 most likely represents a germline-specific, difficult-to-assemble microchromosome[10,11], and clusters 1-18 bona fide chromosomes.

## 1.2) Ensembl.

**1.2.1) Ensembl annotation (Vertebrate Annotation team).** The detailed process of annotation of the hagfish genome can be found in Ensembl site: http://www.ensembl.org/info/genome/genebuild/2018_06_eptatretus_burgeri_genebuild.pdf.

***1.2.1.1) Methods.*** A set of potential transcripts was generated using two major techniques: primarily through alignment of short read RNA-seq data and also through gap filling with protein-to-genome alignments of a subset of vertebrate proteins with experimental evidence from UniProt (The UniProt Consortium, 2019) vertebrate proteins. The short-read RNA-seq data were sourced from samples generated as part of this study (uploaded to ENA under Study accession number PRJEB21290) from blood, brain, gills, heart, intestine, kidneys, liver, testes, skeletal muscle. Data from a second study (PRJNA371391) covering development stages at days 30, 35 and 40 were also used[24]. The UniProt vertebrate proteins had experimental evidence for existence at the protein or transcript level, termed protein existence (PE) level 1 and 2 proteins. Due to the high evolutionary distance from most species to hagfish, all vertebrate PE1/2 proteins were downloaded and aligned in order to increase sensitivity for detecting coding exons. At each locus, low quality transcript models were removed, and the data were collapsed and consolidated into a final gene model plus its associated non-redundant transcript set. When collapsing the data, priority was given to models derived from transcriptomic data. For each putative transcript, the coverage of the longest open reading frame was assessed in relation to known vertebrate proteins, to help differentiate between true isoforms and fragments. In loci where the RNA-seq data were fragmented or missing, homology data took precedence, with preference given to longer transcripts that had strong intron support from the short read data. Gene models were classified, based on the alignment quality of their supporting evidence, into three main types: protein-coding, pseudogene,

and long non-coding RNA. Models with hits to known proteins, and few structural abnormalities (i.e. they had canonical splice sites, introns passing a minimum size threshold, low level of repeat coverage) were classified as protein-coding. Models with hits to known protein, but having multiple issues in their underlying structure, were classified as pseudogenes. Single-exon models with a corresponding multi-exon copy elsewhere in the genome were classified as processed pseudogenes. Models with transcriptomic support, but no evidence of a protein-coding ORF were listed as potential lncRNAs. Potential lncRNAs were further filtered to remove transcripts that had overlap with a protein coding gene (regardless of strand) or if the transcript had a signature of a protein domain. Single exon lncRNAs were filtered out due to lack of evidence to separate them from background transcriptional noise. A separate pipeline was run to annotate small non-coding genes. miRNAs were annotated via a BLAST[25] of miRbase[26] against the genome, before passing the results into RNAfold[27]. Poor quality and repeat-ridden alignments were discarded. Other types of small non-coding genes were annotated by scanning Rfam[28] against the genome and passing the results into Infernal[29].

***1.2.1.2) Results.*** *Building tissue- and development stage-specific transcript models*: A breakdown of the number of transcript structures identified across the nine tissues and three development stages, in addition to the merged sample that contained all mapped reads can be seen in Supplementary Table 11. The number of structures identified per sample ranged from 21,748 from skeletal muscle to 40,619 in the merged sample. Samples with relatively low (blood, skeletal muscle, liver) or high (brain, testis, embryo) counts of annotated transcript structures were in line with observations from other species annotated with the Ensembl gene annotation system.

*Final gene set*: Counts genes and transcripts of the finalised gene set can be seen in Extended Data Figure 1b. The gene annotation of hagfish was challenging due to the large evolutionary distance

between it and other annotated vertebrates. A lot of the data available for vertebrates are biased towards mammals, with human and mouse in particular having the most well annotated genomes. As such, the annotation relied heavily on the transcriptomic data over homology-based structures wherever possible. The protein-coding gene count of 16,513 is likely to be an underestimate of the true number of coding genes and more representative of highly conserved or highly expressed genes. The annotation of hagfish was made available as part of Ensembl version 93 (July 2018).

**1.2.2) Ensembl comparative analyses (Compara team).** Hagfish (*E. burgeri*, Eburgeri_3.2) was included in the Ensembl comparative analyses from its version 93. The analyses include phylogenetic trees (on both coding and non-coding genes), orthology and paralogy relationships, as well as whole-genome alignments with some model organisms.

***1.2.2.1) Phylogenetic tree – whole genomes.*** We started with building a phylogenetic tree of all Ensembl species using the pairwise Mash[30] distances (computed on the whole, unmasked, genomes). In this analysis, hagfish is closer to the Cyclostomata ancestral node than lamprey (*Petromyzon marinus*, Pmarinus_7.0) with a distance of about 0.13 vs. 0.17.

- Download links: https://ftp.ensembl.org/pub/release-93/compara/species_trees/ensembl_species-tree_Ensembl.nh

***1.2.2.2) Whole-genome alignments.*** The hagfish genome was aligned using LastZ[31] (version 1.04) against lamprey, zebrafish (*Danio rerio*, GRCz11), and human (*Homo sapiens*, GRCh38). For all three alignments, we used our standard parameters for vertebrates (Supplementary Table 13). In short, the pipeline[32] does an all-vs-all comparison of two genomes using LastZ, and then smoothens the alignment blocks by building *chains* and *nets*[33]. Only about 0.5% of the hagfish genome (between 12 and 13 Mb) could be aligned to either of the three species. As in other long-

distance alignments, most of the alignment (49.4% of hagfish-lamprey, 59.9% of hagfish-zebrafish, 63.3% of hagfish-human) is concentrated on the coding exons, covering respectively 26.4%, 32.2%, and 31.7% of these. The hagfish alignments cover more of the zebrafish and human genomes (resp. 21.6 Mb and 22.0 Mb) than the lamprey alignments (resp. 19.3 Mb and 17.0 Mb).

- Download links:

https://ftp.ensembl.org/pub/release-93/ensembl-compara/pairwise_alignments/pmar_pmarinus_7.0.v.ebur_eburgeri_3.2.lastz_net/

https://ftp.ensembl.org/pub/release-93/ensembl-compara/pairwise_alignments/drer_grcz11.v.ebur_eburgeri_3.2.lastz_net/

https://ftp.ensembl.org/pub/release-93/ensembl-compara/pairwise_alignments/hsap_grch38.v.ebur_eburgeri_3.2.lastz_net/

***1.2.2.3) Phylogenetic trees and orthologies***. We computed phylogenetic trees on the genes from a set comprising hagfish and 100 other genomes in Ensembl, mostly vertebrates, on coding and non-coding genes, using the methods described in REFs. 32, 34. From those phylogenetic trees, orthology and paralogy relationships were derived. The first stage of the pipeline is to cluster the genes together into families, from blast-derived distances. Statistics about the resulting clusters are presented in Supplementary Table 14. In short, hagfish's non-coding gene set is too small to pursue meaningful statistics. Hagfish's coding set is about half larger than lamprey's, making the comparison of the respective statistics of both species hard. Then, for each cluster, the pipeline pulls and aligns the protein sequences, then builds a tree on the back-translated alignments with TreeBest, which itself makes a consensus of trees built with different methods and substitution models. The trees are reconciled with the species tree, and pairwise relationships (incl. orthologies)

are derived. Overall, hagfish has about 7,500 orthologues with lamprey, and 11,000 with zebrafish and human, as seen in Supplementary Table 15. In all three cases, more than 5,000 of them are in a single-copy in both species. In contrast (Supplementary Table 16), lamprey has about 8,000 orthologues with zebrafish and human, about 4,000 of them being in single-copy, but lamprey's gene annotation is far smaller than hagfish's. For each pair of orthologues, we compare whether the loci were aligned by LastZ, and flag the orthologues that have sufficient LastZ coverage and sequence identity [https://jul2018.archive.ensembl.org/info/genome/compara/Ortholog_qc_manual.html] as "high confidence". Hagfish has about 4,500 high-confidence orthologues with lamprey, and 7,100 with zebrafish and human. We also systematically compare certain gene features across species, and flag genes that are split across several models, and genes that are significantly shorter or longer than their counterpart in other species. However, these comparisons are less reliable when there are few closely related species. Figures of hagfish and lamprey (Supplementary Table 17) must be interpreted with caution.

- Download links: https://ftp.ensembl.org/pub/release-93/emf/ensembl-compara/homologies/Compara.93.protein_default.cds.fasta.gz

https://ftp.ensembl.org/pub/release-93/emf/ensembl-compara/homologies/Compara.93.protein_default.nhx.emf.gz

https://ftp.ensembl.org/pub/release-93/emf/ensembl-compara/homologies/Compara.93.ncrna_default.nt.fasta.gz

https://ftp.ensembl.org/pub/release-93/emf/ensembl-
compara/homologies/Compara.93.ncrna_default.nhx.emf.gz

https://ftp.ensembl.org/pub/release-93/tsv/ensembl-
compara/homologies/eptatretus_burgeri/Compara.93.protein_default.homologies.tsv.gz

https://ftp.ensembl.org/pub/release-93/tsv/ensembl-
compara/homologies/eptatretus_burgeri/Compara.93.ncrna_default.homologies.tsv.gz

https://ftp.ensembl.org/pub/release-93/mysql/ensembl_compara_93/gene_member_qc.txt.gz

## 2. TIME-CALIBRATED PHYLOGENOMICS.

Whether hagfish form a clade with lampreys (Cyclostomata) or represent the sister to all other vertebrates (including lampreys) has, for a long time, depended on whether molecular or morphological evidence are considered. Morphological studies have tended to prefer Cyclostome paraphyly[35–41]. However, Heimberg et al.[42] demonstrated that this has been a consequence of the uncritical recycling of out-dated phenotype datasets and, after revision, they cannot discriminate statistically between cyclostome monophyly and paraphyly. Subsequent analyses of morphological data have recovered cyclostome monophyly[43,44] though it is not clear that they can reject cyclostome paraphyly[35,37]. Phylogenies inferred from molecular evidence have almost exclusively recovered cyclostome monophyly[42,45–50]. Further confusion arises from a paucity of genomic data from hagfish, addressed in this publication.

### 2.1) The phylogeny of vertebrates.

The time calibrated, reconstructed phylogeny of vertebrates with outgroup rooting (main text Figure 2 and Extended Data Figure 2b) supports monophyletic cyclostomes. All nodes were recovered with a posterior probability of 1 and topology of the rest of the tree reflects our current understanding of relationships of vertebrates.

We analysed the gene duplication and loss histories of all gene families in order to test different hypotheses of the root of vertebrates. An approximately unbiased test[51] strongly rejected hagfish sister (log likelihood difference = 7947.7, AU = 0.996, multiscale bootstrap probability < 0.001). Usual bootstrap probability, Bayesian posterior probability, a Kishino-Hasegawa test[52], a Shimodaira-Hasegawa test[53], a weighted Kishino-Hasegawa test and a weighted Shimodaira-Hasegawa test all gave a probability of monophyletic cyclostomes > 0.999.

Duplication events inferred from OrthoFinder (v2.3.5), with greater than 50% support, are strongly enriched in the vertebrate, gnathostome, lamprey and teleost stem branches. Within vertebrates, actinopterygians and cyclostomes are also inferred to have many gene duplication events present (Extended Data Figure 2b).

By computing the likelihood of the gene gain and loss histories of gene families in vertebrates, we show that they are significantly more likely under monophyletic cyclostomes, providing another line of evidence for the monophyly of Cyclostomes. This corroborates other molecular studies, and one recent morphological study[44]. We suggest that the position of hagfishes in the tree of life has now been fully resolved.

**2.2) Calibrations.**

The calibrations are described with respect to node labels in Supplementary Figure 5. New calibrations are fully justified; where we follow previously justified calibrations, we cite the source and provide a summary justification.

**Node 1: Crown Metazoa | 574-609 Ma.**

**Fossil taxon and specimen:** *Charnia masoni* (OUM ÁT.429/p) from level DRK-10 within the Drook Formation (sensu Matthews et al.[54]) at Mistaken Point, Newfoundland[55].

**Minimum justification:** *Charnia masoni* has been interpreted as a stem eumetazoan by Dunn et al.[56]. DRK-10 within the Drook Formation at Mistaken Point has been directly dated to 574.17 Ma ± 2.8 Myr through Zircon U/Pb dating 573.4 Ma ± 0.28 Myr using Pb/Pb dating[54]. However, Matthews et al.[54] demonstrate and Yang et al.[57] argue that rangeomorphs have been found in the rock record from 574 Ma.

**Soft maximum justification:** Lantian biota - this biota has extensive macrofossils but nothing that can definitively be classified as metazoans. Age from Yang et al.[57]. This age allows for the possibility of *Eoandromeda* being a crown metazoan.

**Node 2: Crown Eumetazoa | 561.1-590.8 Ma.**

**Fossil taxon and specimen:** *Auroralumina attenboroughii* (GSM 106119; British Geological Survey, Nottingham, UK) from Bed B, Bradgate Formation, Maplewell Group, Charnian Supergroup, Leicestershire, UK[58].

**Minimum justification:** *Auroralumina attenboroughii* has been shown to be a crown-group member based on tetraradial periderm with corner sulci, allying it with medusozoans[58]. It is from Bed B, Bradgate Formation, Charnian Supergroup, which has a minimum age of 563 ± 1.9 Ma, or 561.1 Ma[59]. Newer U-Pb data suggest the minimum age of the Bradgate Formation is 556.6 ± 6.4, however this large age uncertainty entirely encompasses the maximum age for the clade. For this reason, the age of 561.1 Ma, based on Wilby et al.[59], will be used.

**Soft maximum justification:** Weng'an biota[57] which may contain total group metazoans[60], but there is no convincing evidence of crown metazoans.

**Node 3: Crown Bilateria | 532-590.8 Ma.**

Our minimum constraint on crown-Bilateria follows the justification for crown-Mollusca in Benton et al.[61].

**Minimum justification:** *Aldanella janjiahensis*, or *Aldanella attleborensis*[62] is a widely accepted stem group gastropod dated to 532 Ma

**Soft maximum justification:** Weng'an biota[57] which may contain total group metazoans[60], but there is no convincing evidence of crown metazoans.

**Node 4: Crown Protostomia | 532-590.8 Ma.** Our minimum constraint on crown-Bilateria follows the justification for crown-Mollusca in Benton et al.[61].

**Minimum justification:** *Aldanella janjiahensis*, or *Aldanella attleborensis* is a widely accepted stem group gastropod[63], the oldest record of which is dated to 532 Ma[62].

**Soft maximum justification:** Weng'an biota[57] which may contain total group metazoans[60], but there is no convincing evidence of crown metazoans.

**Node 5: Mollusca-Annelida | 532-590.8 Ma.** Our minimum constraint on crown-Bilateria follows the justification for crown-Mollusca in Benton et al.[61].

**Minimum justification:** *Aldanella janjiahensis*, or *Aldanella attleborensis* is a widely accepted stem group gastropod[63], the oldest record of which is dated to 532 Ma[62].

**Soft maximum justification:** Weng'an biota[57] which may contain total group metazoans[60], but there is no convincing evidence of crown metazoans.

**Node 6: Myriapoda-Pancrustacea: Mandibulata | 514-543 Ma.** Our minimum constraint on crown-Mandibulata follows the justification in Benton et al.[61] and Wolfe et al.[64].

**Minimum justification:** *Yicaris dianensis* and *Wijicaris muelleri* both have limb characteristics that place them within crown Crustacea[61,64].

**Soft maximum justification:** *Rusophycus* trace fossils are widely accepted to be made by arthropod-grade organisms with bilateral symmetry and evidence of segmented limbs (age from REFs. 61, 64, 65. They evidence the existence of arthropods, but there is no evidence of mandibulates at this time.

**Node 7: Crown Deuterostomia | 517.32-590.8 Ma.** Our minimum constraint on crown-Deuterostomia follows the justification in Benton et al.[61].

**Minimum justification:** *Haikouichthys ercaicunensis* from the Chengjiang Biota have been identified as a crown chordate on the basis of branchial structures, myomeres, and a notochord[66]. The age of the Chengjiang Biota was established by Yang et al.[67].

**Soft maximum justification:** Weng'an biota[57] which may contain total group metazoans[60], but there is no convincing evidence of crown metazoans.

**Node 8: Crown Ambulacraria | 515.15-590.8 Ma.**
Our minimum constraint on crown-Ambulacraria follows the justification in Benton et al.[61].

**Minimum justification:** Isolated pelmatozoan columnals that have characteristic morphology and stereom structure of the echinoderm total group[68,69].

**Soft maximum justification:** Weng'an biota[57] which may contain total group metazoans[60], but there is no convincing evidence of crown metazoans.

**Node 9: crown-Chordata | 517.32-590.8 Ma.**
Our minimum constraint on crown-Chordata follows the justification in Benton et al.[61].

**Minimum justification:** *Haikouichthys ercaicunensis* from the Chengjiang Biota have been identified as a crown chordate on the basis of branchial structures, myomeres, and a notochord[66]. The age of the Chengjiang Biota was established by Yang et al.[67].

**Soft maximum justification:** Weng'an biota[57] which may contain total group metazoans[60], but there is no convincing evidence of crown metazoans.

**Node 10: crown-Olfactores | 517.32-590.8 Ma.**
Our minimum constraint on crown-Olfactores follows the justification in Benton et al.[61].

**Minimum justification:** *Haikouichthys ercaicunensis* from the Chengjiang Biota have been identified as a crown chordate on the basis of branchial structures, myomeres, and a notochord[66]. The age of the Chengjiang Biota was established by Yang et al.[67].

**Soft maximum justification:** Weng'an biota[57] which may contain total group metazoans[60], but there is no convincing evidence of crown metazoans.

**Node 11: crown-Vertebrates | 497-590.8 Ma.**

**Fossil taxon and specimen**: *Furnishina bigeminata* (GMPKU2589; Geological Museum of Peking University, Beijing) from the middle Cambrian Huaqiao Formation, Wangcun section[70].

**Minimum justification:** Conodonts are tooth-like 'elements' that have been interpreted as coming from vertebrates, possibly stem gnathostomes[71]. Soft tissue fossils show notochords, segmental trunk musculature, a caudal fin, paired sensory organs, and gill pouches, all of which demonstrate a vertebrate affinity. The oldest conodont is a *Furnishina*, a paraconodont in the euconodont lineage[72] and the earliest records of *Furnishina* are from the middle Cambrian Huaqiao Formation at Wangcun section, dated to 497 Ma[70].

**Soft maximum justification:** Weng'an biota[57] which may contain total group metazoans[60], but there is no convincing evidence of crown metazoans.

**Node 12: crown-Gnathostomata | 420.7-468.4 Ma.**
Our minimum constraint on crown-Gnathostomata follows the justification in Benton et al.[61].

**Minimum justification:** *Guiyu oneiros* has lobe-finned fish synapomorphies which identify it as a stem sarcopterygian and therefore crown Osteichthyes and crown Gnathostomata[61,73].

**Soft maximum justification:** Stem group gnathostomes ('ostracoderms') are present in the Ordovician, but there are no crown representatives in any of the well preserved fossil localities. This suggests the crown group was not yet present[61].

**Node 13: crown-Osteichthyes | 420.7-444.9 Ma.** Our minimum constraint on crown-Osteichthyes follows the justification in Benton et al.[61].

**Minimum justification:** *Guiyu oneiros* has lobe-finned fish synapomorphies which identify it as a stem sarcopterygian and therefore crown Osteichthyes and crown Gnathostomata[61,73].

**Soft maximum justification:** Early Silurian rocks have diverse jawless fishes, but there is nothing that is clearly crown-osteichthyan. The Llandovery can therefore provide the maximum age[61].

**Node 14: crown-Sarcopterygii | 408-427.9 Ma.** Our minimum constraint on crown-Sarcopterygii follows the justification in Benton et al.[61], which was based on *Youngolepis* sp. from the lower Xishacun Formation, Quijing, Yunnan, China (IVPP V10519.1, Institute of Vertebrate Palaeontology and Palaeoanthropology, Beijing). We defer to Benton et al.[61] for the phylogenetic and age justification of the soft maximum constraint.

**Node 15: crown-Rhipidistia | 408-427.9 Ma.** Our minimum constraint on crown-Rhipidistia follows the justification in Benton et al.[61], which was based on *Youngolepis* sp. from the lower Xishacun Formation, Quijing, Yunnan, China (IVPP

V10519.1, Insitute of Vertebrate Palaeontology and Palaeoanthropology, Beijing). We defer to Benton et al.[61] for the phylogenetic and age justification of the soft maximum constraint.

**Node 16: crown Tetrapoda: 337-351 Ma.**

Our minimum constraint on crown-Tetrapoda follows the justification in Benton et al.[61].

**Minimum justification:** *Lethiscus stocki* is either a Lepospondyli (within Batrachomorpha) or a Reptiliomorpha, but since both are crown Tetrapoda it can be safely interpreted as a crown tetrapod[61].

**Soft maximum justification:** Although both baphetids and colosteids are nearer the crown of Tetrapoda, both are found in much younger deposits. Whatcheerids are more distantly related, but are found in older deposits, so these are used as the maximum age constraint - crown tetrapods are not found alongside them[61].

**Node 17: crown Amniota: 318-332 Ma[61].**

Our minimum constraint on crown-Amniota follows the justification in Benton et al.[61].

**Minimum justification:** The eureptilian *Hylonomus lyelli* is a stem diapsid, making it a crown amniote[61].

**Soft maximum justification:** The East Kirkton locality has diverse batrachomorphs and reptiliomorphs, but no diapsids or synaposids, and older sites show the same thing[61].

**Node 18: crown-Reptilia: 255.9-295.9 Ma.** Our minimum constraint on crown-Reptilia follows the justification in Benton et al.[61] which is based on *Protorosaurus spenceri* Meyer, 1832 (Royal College of Surgeons, RCSHC/Fossil Reptiles 308), holotype of the oldest archosauromorph, from the Kupferschiefer of Germany and the Marl Slate of NE England[74]. We defer to Benton et al.[61] for the phylogenetic and age justification of the soft maximum constraint.

**Node 19: crown-Archelosauria | 250.6-295.9 Ma.** Our minimum constraint on crown-Reptilia follows the justification in Benton et al.[61] which is based on *Proterochersis robusta* which is the oldest unequivocal member of total-group Testudines. Though its phylogenetic placement within crown or stem[75,76] Testudines is contested, its membership of total-group Testudines is not. We defer to Benton et al.[61] for the phylogenetic and age justification of the soft maximum constraint.

**Node 20: crown Neognathae | 66.0-86.8 Ma.** Our minimum constraint on crown-Neognathae follows the justification in Benton et al.[61] which is based on *Vegavis iaai* (holotype, Museo de la Plata, MLP 93-I-3-1), a partial skeleton from lithostratigraphic unit K3 of Vega Island, Antarctica. We defer to Benton et al.[61] for the phylogenetic and age justification of the soft maximum constraint.

**Node 22: crown-Mammalia | 176.03-252.2 Ma.**

**Fossil taxon and specimens:** *Henosferus molus* (MPEF 2353; Museo Paleontológico Egidio Feruglio, Trelew, Patagonia, Argentina) from strata belonging to the lowermost Cañadón Asfalto Formation (or beds transitional with the underlying Lonco Trapial Formation) at Queso Rallado[77,78].

**Minimum justification:** Rougier et al.(2007) identify *Henosferus molus* as an early diverging australosphenidan (pan-monotreme) based on a formal morphology-based phylogenetic analysis.

**Age justification**: Cúneo et al.[77] established a minimum age for the Cañadón Asfalto Formation of 176.15 ± 0.12 Ma, thus 176.03 Ma.

**Soft maximum justification:** Some stem-monotremes may extend into the Triassic, and to include the possibility of the haramiyids being crown mammals the Permian-Triassic boundary can be used as the soft maximum[79].

**Node 23: crown-Theria | 121.56-169.6 Ma.**

**Node Calibrated:** The clade comprised of Marsupialia and Placentalia.

**Fossil Taxon and Specimens:** *Sinodelphys szalayi* (Chinese Academy of Geological Sciences CAGS00-IG03, articulated holotype specimen).

**Phylogenetic Justification:** *Sinodelphys szalayi* was resolved on stem to Marsupialia, within Theria, by Luo et al.[80,81], Yuan et al.[82] and Bi et al.[83].

**Age Justification:** Fossils from the Jehol biota of northeast China come from Barremian to Aptian-age deposits, although there is some ambiguity about their age[84]. Here we take the youngest

age estimate from tuffs overlying the main fossil-bearing layers, dated to 121.96 Ma ± 0.5 Myr[85], thus 121.56 Ma. We conservatively use the age of the Barremian to define the ages of Jehol specimens, which has an upper margin of 125.0 Ma. Given the possibility that southern, tribosphenic mammals such as *Ambondro* are therian (cf. REF. 86), we set the soft maximum age for Theria in the Bathonian, 168.3 Ma ± 1.3 Myr so 169.6 Ma[85].

**Node 24: crown-Placentalia | 61.6-164.6 Ma.**
Our minimum constraint on crown-Placentalia follows the justification in Benton et al.[61] which is based on the carnivoran *Ravenictis krausei* (UALVP 31175) from the Ravenscrag Formation, Saskatchewan[87,88]. We defer to Benton et al.[61] for the phylogenetic and age justification of the soft maximum constraint.

**Node 25: crown-Boreoeutheria | 61.6-164.6 Ma.**
Our minimum constraint on crown-Boreoeutheria follows the justification in Benton et al.[61] which is based on the carnivoran *Ravenictis krausei* (UALVP 31175) from the Ravenscrag Formation, Saskatchewan[87,88]. We defer to Benton et al.[61] for the phylogenetic and age justification of the soft maximum constraint.

**Node 26: crown-Euarchontoglires | 61.6-164.6 Ma.**
Our minimum constraint on crown-Euarchontoglires follows the justification in Benton et al.[61] which is based on Torrejonian occurences of extinct primate sister taxa such as plesiadapids from

north-eastern Montana, e.g., *Paromomys farrandi* (UCMP 189520)[89]. We defer to Benton et al.[61] for the phylogenetic and age justification of the soft maximum constraint.

**Node 27: crown-Atlantogenata | 56.0-164.6 Ma.**
Our minimum constraint on crown-Atlantogenata follows the justification in Benton et al.[61] which is based on the extinct proboscidean *Eritherium azzouzorum* (MNHN PM69, Paris) from the Sidi Chennane quarries, phosphate bed IIa, lower bone-bed, Ouled Abdoun Basin of Morocco, regarded as upper Paleocene (early Thanetian)[90]. We defer to Benton et al.[61] for the phylogenetic and age justification of the soft maximum constraint.

**Node 28: crown-Marsupialia | 47.6-131.3 Ma.**
Our minimum constraint on crown-Marsupialia follows the justification in Benton et al.[61] which is based on *Djarthia murgonensis* (Queensland Museum, QM F52748)[91], from the early Eocene of Murgon, Australia. Phylogenetic Justification. *Djarthia* possesses a continuous lower ankle joint, a synapomorphy shared with the marsupial clade Australidelphia[91]. The status of Djarthia as a crown marsupial is stronger than that for Khasia[91,92], and thus it serves as a more definitive record of the latest point by which crown marsupials evolved. We defer to Benton et al.[61] for the phylogenetic and age justification of the soft maximum constraint.

**Node 29: crown-Pipoidea | 148.1-201.5 Ma.**
Our minimum constraint on crown-Pipoidea follows that of Cannatella[93] which we reiterate here:

**Node Calibrated:** The divergence between *Xenopus*-*Nanorana* which equates to crown-Pipoidea[93].

**Fossil Taxon and Specimens:** *Rhadinosteus parvus* (holotype DINO 14693, Dinosaur National Monument, Colorado, USA, partial skeleton, articulated to loosely articulated).

**Phylogenetic Justification:** *Rhadinosteus parvus* is the fossil representative of crown-Pipoidea, identified as a stem-rhinophrynid, more closely related to *Rhinophrynus* than Pipidae[94,95].

**Age Justification:** *Rhadinosteus parvus* was recovered from the Brushy Basin Member of the Morrison Formation of Utah[95] which has been dated to 148.1-150.3 Ma[96], thus our minimum constraint on crown-Pipoidea is 148.1 Ma. Our soft maximum constraint on the *Xenopus*-*Nanorana* divergence follows that for crown-Anura in Benton et al.[61] which was based on the Triassic-Jurassic boundary, allowing for finds of stem anurans and other small tetrapods from several continents, but as yet no crown representatives of these clades. The Triassic-Jurassic boundary is dated to 201.3 Ma ± 0.2 Myr[85], and so 201.5 Ma.

**Node 30: crown-Neopterygii | 250.0-331.1 Ma.**

Our minimum constraint on crown-Neopterygii follows the justification in Benton et al.[61], which was based on *Watsonulus eugnathoides*[97] from the Middle Sakamena Formation, Sakamena Group, Ambilombe Bay, Madagascar (syntype MNHN MAE 33a, b, Muséum national d'Histoire naturelle, Paris). We defer to Benton et al.[61] for the phylogenetic and age justification of the soft maximum constraint.

**Node 31: crown-Clupeocephala | 150.94-235 Ma.**
Our minimum constraint on crown-Clupeocephala follows the justification in Benton et al.[61] which is based on *Leptolepides haerteisi(Arratia 1997)* from the Solnhofen Formation, Zandt Member, Zandt, Bavaria, Germany (holotype JM SOS 2473, Jura Museum, Eichstätt, Germany). We defer to Benton et al.[61] for the phylogenetic and age justification of the soft maximum constraint.

**Node 32: Ovalentaria-Tetraodontiformes | 69.71-130.8 Ma.**
Our minimum constraint on the divergence between Ovalentaria and Tetraodontiformes follows the justification in Benton et al.[61] which is based on *Cretatriacanthus guidottii* from the 'Calcari Melissano' (of historical usage) at Canale near Nardò, Italy (holotype MCSNV 1377, Museo Civico di Storia Naturale, Verona, Italy). We defer to Benton et al.[61] for the phylogenetic and age justification of the soft maximum constraint.

**Node 33: crown-Chondrichthyes | 333.56-422.4 Ma.**
Our minimum constraint on crown-Chondrichthyes follows the justification in Benton et al.[61], which was based on *Chondrenchelys problematicus* from Mumbie Quarry, Glencartholm Volcanic Group, Upper Border Group of the Calcifererous Sandstone, Glencartholm, Scotland (NMS 1998.35.1, National. We defer to Benton et al.[61] for the phylogenetic and age justification. We defer to Benton et al.[61] for the phylogenetic and age justification of the soft maximum constraint.

**Node 36: crown-Cyclostomata | 358.5-614 Ma.** Our minimum constraint on crown-Cyclostomata follows the justification in Benton et al.[61], which was based on *Priscomyzon riniensis* (Albany Museum, Grahamstown, Eastern Cape, South Africa, catalogue number AM5750), holotype, consisting of a whole dorso-ventrally compressed organism. We defer to Benton et al.[61] for the phylogenetic and age justification. Our soft maximum constraint is based on the Ediacaran Weng'an Biota which preserves a diversity of multicellular eukaryotes preserved in subcellular fidelity, but which has yielded no evidence of chordates[98]. The Weng'an Biota has been dated to 609 Ma ± 5 Myr[99], hence we use it to evidence a 614 Ma soft maximum constraint on the age of crown-Chordata.

## 3. GENE FAMILY EVOLUTION

### 3.1) Gene family gains and losses across vertebrate evolution.

We used comparative genomic analyses (see Methods) to infer the gene complements of different ancestors within deuterostomes. The ancestral vertebrate genome was composed of at least 9952 gene families, from which ~17,000 modern human genes descend. The most abundant biological functions in the ancestral vertebrate genome are related to metabolite interconversion, regulation of transcription, and transmembrane proteins involved in signal reception and transport. The most predominant pathways are related to Wnt, inflammation, gonadotropin-releasing hormones, integrins, and angiogenesis (Supplementary Table 23).

Gnathostomata and Vertebrata are among the nodes with the largest amount of gene gains and lowest number of losses (respectively Novel Homology Groups (HGs) and Lost HGs, Figure 2, Extended Data Figure 3a and Supplementary Table 24), supporting a major role for genomic novelty and conservation in the origins of those lineages (see Novel Core HG below). On the other hand, Cyclostomata and Olfactores are among the clades showing the largest number of gene losses (Figure 2, Extended Data Figure 3a). The gene gains and loss values observed here are similar to the numbers observed in other major metazoan transitions in previous studies[100,101].

The evolution of gene novelties and their functions across the different nodes shows that the Novel HG of the LCA of vertebrates are dominated by genes involved in signalling, cell adhesion, and regulation of transcription (e.g., 'gene-specific transcriptional regulator', 'nucleic acid binding protein'; Supplementary Table 24, Extended Data Figure 3b). The Novel HGs of the LCA of gnathostomes display many novel genes related to the immune system (Supplementary Table 24).

An interesting subset of novel genes is those highly retained after their emergence (Novel Core HG), as they may contain genes of high functional relevance. Cyclostomata, Vertebrata, and Gnathostomata show the highest numbers of conserved gene novelties (Novel Core HG, Figure 2, Extended Data Figure 3a). This is consistent with the patterns of WGD described elsewhere in this study, and hints at the importance of novelty over loss in these three clades. These numbers here are indeed larger than the equivalent values for other metazoan transitions; for example, here Cyclostomata, Vertebrata, and Gnathostomata respectively have ++ 98, ++ 86, and ++ 81 Novel Core HG, compared to ++ 25 in Metazoa, ++ 0 in Bilateria, ++ 1 / ++ 5 in the three bilaterian superclades[100,101].

Our analysis found 5 Novel Core HG in chordates, including the ZHX homeobox genes family, a keratin gene (KRT), or MARCH7 which is an ubiquitin involved in immune response. As in the case of Novel HG, vertebrate lineages have retained many Novel Core HGs that are also involved in signalling (Supplementary Table 25); thus, signalling genes not only were predominant among the novel genes, but they were also highly retained later across all vertebrate lineages. Similarly, gnathostome Novel Core HGs (Supplementary Table 26) shows that over 71% of conserved novel genes are immunity genes, also seen among the Novel HG (Supplementary Table 26, Extended Data Figure 3b). Gnathostome lineages retained genes involved in gene regulation (gene-specific transcriptional regulators, nucleic acid binding protein) compared to their Novel HGs.

**3.2) Analysis of hagfish gene families of interest.**

**3.2.1) Immune system related genes.** Due to their pivotal phylogenetic position, the agnathans serve as model organisms for understanding the origin and early evolution of the vertebrate

adaptive immune system[102]. Both hagfish and lamprey possess B-like and T-like lymphocytes, but unlike the jawed vertebrates which use immunoglobulin domain-based anticipatory receptors, they use leucine-rich repeat (LRR)-based variable lymphocyte receptors (VLRA, VLRB and VLRC)[102]. We carried out similarity searches against the *E. burgeri* genome using available assembled hagfish *VLR* cDNAs. In the *E. burgeri* genome sequence, the majority of *VLR* donor cassettes and three germline *VLR* genes are found on three large scaffolds (Supplementary Figure 6). The *VLRB* and *VLRC* germline genes are located next to 94 and 19 donor cassettes, respectively, on assembly cluster15 (Supplementary Figure 6a), while the *VLRA* germline gene flanks 14 donor cassettes on cluster12 (Supplementary Figure 6b). Two additional clusters containing 16 and 36 *VLRA*-like donor *LRRV* cassettes are separated by 4.6 Mbp on cluster1 (Supplementary Figure 6b). Shared *LRR* cassette usage for *VLRA* and *VLRC*[103] suggests that a conserved functional relationship could exist between the VLRA- and VLRC-producing T-cell lineages in hagfish (Supplementary Figure 6c). The analysis of the hagfish genome assembly reveals that it harbours gene homologues of important genes that function in adaptive and innate immunity in jawed vertebrates, although orthology is complicated by the divergent genomic history of the agnathans (e.g., *GATA2/3* and *TLR13/21*) (Supplementary Table 27). Our results indicate that despite the differences in the anticipatory receptors in jawed and jawless vertebrates, several fundamental components of lymphocyte development and regulation were present in the common ancestor of living vertebrates.

***Methodology***: Variable lymphocyte receptor BLAST searches of the *E. burgeri* genome sequence assembly were carried out using sequence fragments taken from available hagfish *VLR* cDNA sequence. Accession numbers are as follows: *E. burgeri VLRC* (initially labeled as VLRA):

AY964719.1-AY964729.1, AY964731.1-AY964762.1, AY964764.1-AY964782.1, AY964786.1-AY964789.1; *E. burgeri VLRB*: AB519991.1-AY965525.1, AY965527.1-AY965534.1; *E. stoutii VLRA* (third VLR): KF314046.1-KF314109.1; *E. stoutii VLRC*: AY964837.1, AY964783.1-AY964785.1, AY964790.1-AY964809.1, AY964811.1-AY964822.1,AY964825.1-AY964836.1, AY964838.1-AY964872.1, AY964874.1-AY964881.1, AY964883.1-AY964906.1, AY964908.1-AY964918.1 and AY964920.1-AY964931.1. Fragments used in the searches were as follows: LRRNT, LRR1, LRRV, LRRVe-CP, LRRCT, germline signal peptide and germline C-terminal regions. Both BLASTN and TBLASTN (using translated fragment sequences) were carried out using expect values of e-value ≤ 10e-6, although higher expect values were sometimes used to detect short sequence matches to connecting peptide (CP) and LRR1 sequences. All matches were individually inspected.

**3.2.2) Hagfish vision related genes.** The vertebrate visual pathway includes a diversity of proteins conserved across the entire clade. However, how this pathway was assembled in Deuterostomia remains unclear, and multiple lineage-specific gene-losses across Vertebrata confuse its subsequent evolution. While lamprey genomes have been available for a while, the absence of a hagfish genome, together with some uncertainty on the evolutionary relationships between the hagfishes –with their simpler and perhaps secondarily degenerated eyes[104]—, the lampreys and the gnathostomes, hampered progress in our understanding of the evolution of vision at the root of the vertebrate tree.

We investigated patterns of presence and absence in 49 vision-associated proteins across 39 deuterostomes, including the hagfish *E. burgeri*. Our results show that while a subsample of these genes (average = 13.2) is shared across all deuterostomes, cyclostomes have approximately

twice as many visual genes (Average = 30.5), and *E. burgeri* has 27. The average number of genes associated with vision further increase in the gnathostomes, with an average of 42.5 visual genes.

Three visual pigments (Opsins) were found in the genome of *E. burgeri*: Melanopsin (Opn4), Peropsin (RRH) and Rhodopsin 1 (RH1 or RHO) (Supplementary Figure 7). In vertebrates, Melanopsin is expressed in ipRGCs cells or their homologues[104] and this opsin is not involved in processes of image-forming vision. Differently, RH1 is known to be expressed in rod cells and is generally involved in dim-light (scotopic) image forming vision[104–106]. Peropsin[107,108] is also not directly involved in the process of image-forming vision. However, Peropsin plays a key role (together with the RGR Opsin – that we did not detect in the hagfish) in re-isomerising (recycling) *all-trans* retinal to *11-cis* retinal[109]. In the process of vertebrate vision, *11-cis* retinal, which is attached to opsins, like the RH1 opsin, through the retinal binding domain, transforms into *all-trans* retinal after being hit by a photon of light. *All-trans* retinal is then detached from its opsin (a process known as opsin bleaching). *All-trans* retinal is re-isomerised to *11-cis* retinal and reattached to visual opsins so that they can be re-used in the process of image-forming vision. Peropsin (RRH), that we found in the hagfish genome, is one of the key proteins in the process of retinal re-isomerisation[109], and its presence in the hagfish genome is evidence that RH1 is functional in the hagfish. None of the other vertebrate opsins used for image-forming vision (LWS, SWS1, SWS2 and RH2) are found in the genome of *E. burgeri*. However, these genes have previously been identified in lampreys, with the pouched lamprey *Geotria australis*, for example, possessing the full complement of vertebrate visual pigments[106]. These observations suggest that cones and rods, as well as cone-mediated bright-light colour vision and rod-mediated dim-light vision, most likely emerged in the stem vertebrate lineage[106], and that *E. burgeri* secondarily lost all its visual opsins with the exclusion of RH1. The hagfish eye has been suggested to be

degenerate, and its retina only seems to include one type of photoreceptor cell. While the morphology of these photoreceptors cannot discriminate whether the hagfish retina is composed of modified cones or rods, Lamb[104] has pointed out that "hagfish photoreceptors exhibit some rod-like properties". Our results agree with Lamb's assertion.

The emergence of both cones and rods in the stem vertebrate lineage is confirmed by the analysis of visual pathway genes (Supplementary Figure 7). Six of the 11 candidate genes that are known to be involved in both the cone and rod opsin pathways (PDC, GPSM2, GNB5, GUCY2F, RGS9 and RGS9BP) were present in the last common ancestor of all Deuterostomia, and predate the origin of cones, rods and of the cone- and rod-specific opsins. The remaining 5 (RCVRN, GRK1, GUCA1B, GUCY2D, and GUCA1A) are present in both Cyclostomata and Gnathostomata, but are not found outside Vertebrata; suggesting that these genes emerged in the stem vertebrate lineage. In Cyclostomata, *E. burgeri* secondarily lost GUCA1A and GUCY2D, while the brook lamprey (*Lampetra planeri*) lacks GNB5, RGS9 and RGS9BP. *L. planeri* and *E. burgeri* also shared the absence of RCVRN and GRK1, while *L. planeri* and a second lamprey (Arctic lamprey, *Lethenteron camtschaticum*) miss GUCA1A (see Supplementary Figure 7).

Ten genes that are exclusively expressed in cones were considered. Three of these genes (CNGB3, SLC24A2, CNGA3) predate the emergence of the cone opsins, being present across all Deuterostomia. Six genes (ARR3, GRK7, GNAT2, GNGT2, PDE6H and PDE6C) emerge in the stem vertebrate lineage, while GNB3 is gnathostome-specific despite having been lost in some Chondrichthyes (see Supplementary Figure 7). Within Cyclostomata, GNAT2, GNGT2 and PDE6H were each only present in one of the studied species: *L. planeri*, *L. camtschaticum* and the sea lamprey *Petromyzon marinus* respectively. The genomes of the brook and Arctic lampreys also lack PDE6C and CNGB3. While the hagfish has the three genes used by cones that are found

across all deuterostomes, only two of the vertebrate-specific cone-expressed genes are present (ARR3 and GRK7). This pattern of losses suggests a loss of cone-associated visual processes in the hagfish (see Supplementary Figure 7).

All rod-opsin pathway specific genes are present in at least one cyclostome, suggesting that this pathway was present in their last common ancestor. As these genes are also broadly distributed across gnathostomes, we can conclude that the rod-opsin pathway also evolved in the stem vertebrate lineage (as suggested by the presence/absence of opsin genes). Three rod-expressed genes CNGB1, CNGA1 and SLC24A1 are broadly distributed in Deuterostomia, indicating a longer evolutionary history, similar to their cone-expressed paralogs (CNGB3, CNGA3, SLC24A2), and a broader, non-rod specific, functional role. Five rod-specific genes (GNGT1, PDE6B, PDA6A, CNGB1 and CNGA1) could not be found in *E. burgeri.* As these genes are broadly distributed across Deuterostomia, it is possible that some of these genes might not have been sequenced or they might have been incorrectly annotated. GNGT1, on the other hand, is absent from all cyclostomes and seems gnathostome-specific (see Supplementary Figure 7).

The level of conservation of genes involved in the visual cascade seems much more variable. Six of the 12 genes we considered are present across Deuterostomia (RDH8, RDH12, RPE65, RGR, RDH10 and RDH11). However, RDH11 appears to have been independently lost within Hemichordata and Cephalochordata, while RDH8 appears to have been independently lost in Cephalochordata and Urochordata. RBP3 is present in the amphioxus *Branchiostoma belcheri* but could not be found in any other Cephalochordate or Urochordate considered. If the presence of this gene (which is broadly distributed across vertebrates) in *B. belcheri* is not the result of some form of contamination, it would suggest that RBP3 emerged in the stem chordate lineage and was independently lost multiple times in Cephalochordata and Urochordata. Differently, RLBP1

appears to be specific to the Olfactores (tunicates and vertebrates); LRAT, RBP1 and ABCA4 emerge as vertebrate-specific and RDH4 is Gnathostome-specific. Within Cyclostomata, *E. burgeri* and *L. plnaeri* share the absence of RP3 and ABCA4, whilst *L. planeri* and *L. camtschaticum* both lack RDH8. Further independent absences are also observable, such as that of RBP1 in *L. planeri*, and of RDH11 and RGR in *E. burgeri*, respectively.

Given the monophyly of Cyclostomata, our results suggest that the cone and rod opsin specific pathways were established in the stem vertebrate lineage, and that the last common vertebrate ancestor was potentially capable of both bright-light colour vision and dim-light vision. The last common ancestor of the cyclostomes possessed all known visual opsins[106], as well as cone- and rod-specific opsin pathways genes. Hagfishes have eyes with a simplified retina and photoreceptors of unknown type. The presence of only one visual pigment (the rod-specific RH1) in *E. burgeri* and the loss of most cone-specific genes in this taxon, as well as several rod-specific genes, indicates that the hagfish have secondarily simplified eyes, that they lost the ability to see in colour, and that their photoreceptors may be modified rods.

All visual-related genes found in this analysis are provided as a multifasta file in the Supplementary File 2.

***Methods.*** 53 vision-associated and photosensitive proteins were selected based on Emerling and Springer[110], with the addition of the opsin sequences Peropsin and Melanopsin. These genes include those involved in both the cone and rod phototransduction pathways, alongside visual pathway genes and genes associated with visual disorders, and genes involved in light detection that are not directly used for image forming vision (Melanopsin and Peropsin). These genes have previously been used to establish secondary adaptation to low photic environments in mammals[110].

Independent BLAST searches for each of the 53 candidate genes (see Supplementary Figure 7 for a list) were conducted across 39 high coverage genomes selected from across Deuterostomia. Significant hits (e-value < e-20) were subjected to a BLAST search against the nr database in NCBI to confirm identity. Sequences that were positively confirmed as belonging to the candidate gene family were marked as present in Supplementary Figure 7. Those that could not be positively confirmed after these two steps were marked as absent.

**3.2.3) Circadian clock genes.** Cyclostomes are representatives of an ancient vertebrate lineage. The study of the circadian clock and of the non-visual photoreception in this taxon is useful to understand the evolution of the circadian timekeeping system and of photic entrainment (synchronization) in vertebrates.

In gnathostomes, the suprachiasmatic nucleus (SCN) of the hypothalamus, the retina and the pineal gland have been reported to be important sites of circadian oscillations. Hagfishes show daily rhythms of locomotor activity in light-dark cycles with a marked nocturnal pattern[111,112]. The rhythm is maintained in constant darkness demonstrating the existence of a functional circadian clock. Interestingly, the hagfish lack of a pineal gland and experiments of ablation showed that the central pacemaker generating behavioural rhythmicity in hagfish is located in the preoptic nuclei (PON) of the hypothalamus[113]. The light signal is perceived by the retina of the eyes and sent to the PON via the pretectum[114]. Hagfish eyes do not allow an image-forming vision, but they are able to sense light and drive behavioural responses, such as shadow detection and photophobic response[115]. A previous investigation showed the expression of Melanopsin in the ganglion cells of the retina, which indicate that these cells could mediate the photic entrainment[116]. Furthermore,

as in jawed vertebrates, extraocular photoreception has been demonstrated in the hagfish: it has a direct behavioural reaction to external light stimuli via the photoreceptor of the skin[117]. Interestingly, the photic signal through skin does not entrain behavioural circadian rhythms. However, it would be expected that light also penetrates deep into the brain and tissues of hagfishes, thereby participating in photic entrainment. Until now the molecular circadian clock of hagfish has not been characterised.

We surveyed the genome of *E. burgeri* for clock genes and clock-controlled genes, and found a primordial vertebrate circadian system, with fewer orthologous genes, in respect to gnathostomes, that presumably cover all basic functions. In particular, we found the following *bona fide* clock genes: 2 *Clock*, 3 *Bmal*, 1 *Timeless* (*Tim*), 2 *Period* (*Per*), 1 *Cryptochrome* (*Cry*), *Nfil3*-6 (*E4bp4*) and *Tef* (Supplementary Table 28). Interestingly, the opsin repertoire in hagfish (see previous section) is constituted exclusively by a Rhodopsin (RH1 or RHO), the light-sensitive receptor protein involved in phototransduction, a Peropsin (RRH) that recycle *all-trans* retinal to *11-cis* retinal, and a Melanopsin (Opsin 4), necessary for the photic entrainment. It would be very interesting to study in the future their expression profile in the retina, brain, gonads and skin.

Genomic and transcriptomic searches failed, nevertheless, in finding the Melatonin synthesis (Aanat) and Melatonin receptor (MtnR) genes. This is probably correlated with the documented anatomical absence of the pineal gland in hagfish. Conversely, lampreys have the pineal gland, which plays a central role in the generation of circadian locomotor rhythms[118]. We also failed to identify genes involved in the DNA repair, such as *6-4 photolyase* (*Cry5*), *DASH* and *CPD photolyase* (*Cpdp*). As previously shown in cavefish, *Phreatichthys andruzzii*[119], the absence of these genes could be correlated with the adaptation to extreme photic environments.

## 4. MACROSYNTENY ANALYSES OF VERTEBRATE GENOMES

### 4.1) Source of genomic data.

All genome assemblies and protein annotations used for these analyses are provided in Supplementary Table 1.

### 4.2) Definition of homologous genes.

We conducted phylogeny-aware BLASTP (v2.6.0+) genome-wide searches to identify between-species homologs. That is, for analysis between chicken, gar and elephant shark, or between hagfish and sea lamprey, or between sea cucumber and lancelet, we used reciprocal best hit gene pairs as homologs (Supplementary File 3). By contrast, for the alignment between vertebrate species (chicken, spotted gar, elephant shark, lamprey and hagfish) and non-vertebrate species (sea cucumber, amphioxus), we used genes of later species as reference and searched for the one-way best hit gene from the former species as homologs (Supplementary File 3). In this way, we identified multiple homologs in vertebrates corresponding to one gene in non-vertebrate species, which are diverged before WGDs. Since we reconstructed the gene content of 17 ACs by taking amphioxus genes (Belcher's lancelet, *Branchiostoma belcheri*) as surrogates, gene homology relationships between vertebrate species and the inferred ACs were directly proxied from those between vertebrate species and amphioxus. For chicken or gar, we also performed all-against-all BLASTP searches to identify reciprocal interchromosomal best hit pairs as within-species homologs. That is, BLASTP-matched pairs from the same chromosomes or scaffolds were excluded to exclude small scale duplication events.

### 4.3) Definition of homologous chromosomes.

Given the between- and within-species homology relationships at the gene level, we tested whether two chromosomes harbour a significant number of homologous pairs via Chi-Square test. Specifically, we counted that there are *m* genes on a single reference chromosome *B*. There are in total *M* genes on all reference chromosomes. We then identified *n* homologous genes between query chromosome A and reference chromosome B. And there are in total *N* homologous genes harboured by chromosome A relative to all reference chromosomes. We used these four numbers (*n*, *N*-*n*, *m*, *M*-*m*) to conduct the Chi-Square test followed by FDR correction. Homologous gene pair numbers over 5 and FDR q value less than 0.05 are used as the cut-off threshold. This strategy is used across the whole manuscript.

### 4.4) Macrosynteny between chicken and spotted gar.

Although chicken chromosomes and spotted gar linkage groups (LOCs) largely form a one-to-one relationship in terms of distribution of homologous pairs (Supplementary Figure 10), there are a few one-to-many or many-to-one relationships, suggesting chicken- or spotted gar-specific chromosomal fusion or fission events. We polarised such events by taking the shark genome as the outgroup. For example, spotted gar LOC5 is simultaneously homologous to chicken chr6 and chr12. We examined whether chr6 and chr12 of chicken correspond to different sets of shark scaffolds. If true, a spotted gar-specific fusion is inferred. Otherwise, one chicken-specific fission event would be more plausible. To increase the certainty of our inference, we required at least two shark scaffolds to support one scenario. As a result, we identified three spotted gar-specific fissions (LOC9 and LOC11; LOC1 and LOC16; LOC3, LOC17 and LOC14), ten spotted gar-specific fusions (LOC1, LOC3, LOC4, LOC5, LOC6, LOC7, LOC10, LOC12, LOC14, and LOC26), and

three chicken-specific fusions (chr1, 4 and 5). Note that chicken chr1 seems to arise from a fusion of four ancestral chromosomes, while only two chromosomes were involved in the emergence of chr4 or chr5. In addition, we identified one ambiguous case where both spotted gar LOC2 and LOC4 are co-homologous to chicken chr4 and Z, while shark scaffolds support both the linkage of spotted gar LOC2 and LOC4, and the linkage of chicken chr4 and Z. That is, all three species differ in their karyotype, and we thus left this case unclarified.

After polarization, we artificially back-mutated chicken and spotted gar chromosomes to approximate the ancestral karyotype. In other words, for chicken- or spotted gar-specific fusions, we manually split these chromosomes. The boundary was inferred based on the correspondence between chicken chromosomes and spotted gar LOCs. If necessary, elephant shark scaffolds were also used as outgroup to confirm. Taking chicken chr5 as an example, genes from 1.0 Mb to 24.2 Mb (chr5:1.0-24.2 Mb) are defined as chr5.1, which corresponds to spotted gar LOC27 (Supplementary Figure 11). The remaining two parts of chr5:0–1.0 Mb and chr5:24.2 – 59.6 Mb are homologous to spotted gar LOC7 and to the same set of elephant shark genome scaffolds (Supplementary Figure 11). Thus, we grouped them as chr5.2. The situation can be more complex. Taking chicken chr1 subjected to three fusions as an example, genes from chr1:0 Mb-75.7 Mb but excluding a middle region chr1:48.0-52.2 Mb are defined as chr1.1. We grouped chr1:0-48.0 Mb and chr1:52.2-75.7 Mb together since both of them correspond to spotted gar LOC8 or the same set of shark scaffolds (Supplementary Figure 12). Then, chr1:48.0-52.2 Mb, chr1:75.8-78.0 Mb and chr1: 78.1-196.2 Mb are defined as chr1.2, chr1.3 and chr1.4, respectively. By following the same practice, we back-mutated chicken chr4 and 10 gar-specific fusions (Supplementary Figures 13, 14). Analogously, for three gar-specific fissions (LOC9 and LOC11, LOC1 and LOC16, LOC3, LOC14 and LOC17), we manually fused the related chromosomes. Since LOC1, LOC3,

and LOC14 have been already back-mutated due to occurrence of ancestral fusions, we re-evaluated the homologous relationships between these LOCs and shark scaffolds and then fused the corresponding chromosome groups, which were later referred as LOC9LOC11, LOC1.2LOC16, and LOC3.1LOC14.2LOC17.

During the analysis of macrosynteny, we identified six spotted gar-specific chromosomal regions (LOC28, LOC6.3, LOC1.3, LOC2.2, LOC5.3 and LOC24.2) sharing no homology to any chicken chromosome (Supplementary Figure 14). First, spotted gar LOC28 does not share an excess of homologs with any chicken chromosome. Second, when back-mutating spotted gar genome we found spotted gar LOC1.3, LOC5.3 and LOC6.3 are not homologous to any chicken chromosomes. Third, to increase the resolution, we performed a sliding-window (window size of 50 genes) analysis by examining whether less than five out of 50 genes could find homologs between chicken and spotted gar. We further examined whether homologous regions are present (more than 10 out of 50 genes with homolog present) in shark and whether the region is not full (25 out of 50 genes) of small-scale duplicates since these duplicates would confound homolog mapping process. With this fine-scale analysis, we additionally found two spotted gar-specific segments: LOC2:56.8-68.6 Mb (LOC2.2) harbouring 425 genes and LOC24:0-4.5 Mb (LOC24.2) harbouring 174 genes. For them, only 26 and 13 genes harbour homologs scattered across multiple chicken chromosomes. Accordingly, we named the remaining chromosomal regions as LOC2.1 and LOC24.1, respectively. Notably, only LOC2.2 and LOC6.3 could find homologous scaffolds in elephant shark genome, and thus the corresponding regions get lost in chicken.

After back-mutation and identification of gar-specific chromosomal segments, almost all chicken and gar chromosomal segments depict a one-to-one homology (Supplementary Table 31) except the aforementioned ambiguous cases where gar LOC2 and LOC4 are co-homologous to

chicken chr4 and Z, and this co-homology relationship could not be detected in shark scaffolds. With introduction of back-mutation, this case becomes LOC2.1 and LOC4.2 co-homologous to chicken chr4.2 and Z (Supplementary Table 31).

We reasoned that there should be two separate ancient chromosomes before the split, with each mainly contributing to chicken chrZ (spotted gar LOC2.1) and chicken chr4.2 (spotted gar LOC4.2) respectively based on the following two observations: (1) chicken chrZ harboured more (355 vs. 56) homologs with spotted gar LOC2.1, and chicken chr4.2 harboured more (354 vs. 67) homologs with spotted gar LOC4.2 (Supplementary Table 31); (2) both chicken chrZ and spotted gar LOC2.1 are homologous to sea cucumber LG01/LG06 and LG08, while chicken chr4.2 and spotted gar LOC4.2 are homologous to sea cucumber LG09/LG12 and LG19 (Supplementary Table 31, Supplementary Figure 15). Therefore, we used chicken chrZ (spotted gar LOC2.1) and chr4.2 (spotted gar LOC4.2) to represent two ancestral gnathostome chromosomes. Their co-homology could be due to recurrent gene shuffling between this pair of chromosomes in both chicken and gar lineages.

**4.5) Inference of vertebrate ancestral chromosomes.**

**4.5.1) Macrosynteny conservation analysis between sea cucumber, chicken and spotted gar.** Consistent with the prediction of WGD, in either chicken or gar genome, almost all individual chromosomes homologous to one sea cucumber LG shares a significant number of homologs between each other (Supplementary Figure 15). For example, chicken chr15 and 19 (homolog of sea cucumber LG04) shared 70 best-to-best paralogs, which was significant and could not happen by chance. And these genes are scattered across the whole chromosome pair (Supplementary Figure 16). More dramatically, the chromosome-level homology is not unique to chr15 and chr19

but applies for the whole chicken genome (Supplementary Figure 16). Thus, we corroborated the previous conclusion that WGD events rather than multiple independent segmental duplications accounts for these observations.

Notably, in four cases, two or three sea cucumber LGs are simultaneously homologous to the same set of three or four chromosomes in either chicken or spotted gar genome (Supplementary Figure 15; Supplementary Table 32). They are LG01/LG11/LG17 (chicken: chr2, chr7, chr27, chr33), LG01/LG06 (chicken: chrZ, chr10, chr28, chr25), LG09/LG12 (chicken: chr6, chr4.2, chr13, chr22), and LG16/LG22 (chicken: chr2, chr20, chr3). We proposed that these four groups of sea cucumber LGs are very likely to be fused as one ancestral chromosome before gnathostome WGDs. Consistently, we built a phylogenetic tree covering chicken and spotted gar homologous chromosome sets and sea cucumber LGs (like LG01, LG11 and LG17). We concatenated proteins from the same chromosome and observed the same tree topology for LG01, LG11 and LG17 (Supplementary Figure 17). Sea cucumber LG01/LG06, LG09/LG12 and LG16/LG22 represent similar cases (Supplementary Figures 18-20). Therefore, these four cases should represent four pre-WGD fused chromosomes.

Given the homologous relationships between sea cucumber LGs and gnathostome chromosomes, the corresponding sea cucumber LGs and gnathostome chromosomes are divided into 17 separate groups (Supplementary Figure 15, Supplementary Table 32) representing 17 vertebrate ancestral chromosomes (ACs). These 17 ACs contributed to gnathostome karyotype by 2R WGDs. 11 out of these 17 ACs correspond to four chicken or spotted gar chromosomes, and 6 ACs, including AC7, AC8, AC9, AC15, AC16 and AC17 correspond to three (Supplementary Table 32).

**4.5.2) Reconstruction of the gene contents of 17 ACs.** We reconstructed the gene content of 17 ACs based on annotated genes of the Belcher's amphioxus, *B. belcheri*, genome[120] by combining two strategies, as depicted in Supplementary Figure 21:

(1) First, we identified *B. belcheri* scaffolds homologous to sea cucumber LGs (Supplementary Table 33), and used this information to map lancelet scaffolds to the ACs based on the homology relationships between sea cucumber LGs and ACs (the latter depicted in Supplementary Figure 15). Second, with this information (*B. belcheri* scaffold ↔ *A. japonicus* LG → AC), we then mapped a *B. belcheri*'s gene into one specific AC if it met both of the following two conditions: (i) it was located on a scaffold corresponding to this AC (according to the first step above), and (ii) if there was at least one chicken gene and at least one spotted gar gene, both harboured by chromosomes descending from the same AC, having this lancelet gene as their blast best hit (and thus considered homologous). This strategy is depicted in Supplementary Figure 21a.

(2) We also used the direct homology relationship between chicken and/or spotted gar genes and *B. belcheri* genes, independently of sea cucumber LGs: if at least five chicken and spotted gar genes located on five separate chromosomes, in any combination, and all corresponding to the same AC, had the same amphioxus gene as their blast best hit homologous gene, it was mapped to that specific AC.

All in all, 5,065 *B. belcheri* genes were mapped to 17 ACs (Supplementary Table 34), ranging from 115 in AC9 to 534 in AC1, and with the exception of AC17, for which we recovered only 20 genes and was thus excluded from several subsequent analyses.

**4.5.3) Comparing sea cucumber and Florida lancelet genomes as outgroup taxa for macrosynteny conservation analysis.** Our inference of the ancestral vertebrate karyotype was first performed before any chromosome-level genome assembly was available from any amphioxus species, a slow-evolving lineage closer to vertebrates than the sea cucumber (echinoderm). To test the robustness of our inference of 17 vertebrate ACs, we first assessed the macrosynteny conservation between the sea cucumber *A. japonicus* genome and that of the Floridian amphioxus *B. floridae*. Since the latest Florida lancelet chromosome-level assembly (GCF_000003815.2) does not provide a gene annotation, we first found orthologous genes between the sea cucumber genome annotation and the annotated genes of a previous *B. floridae* scaffold-level assembly (GCF_000003815.1) via reciprocal best blast hits. Then, genes from the GCF_000003815.1 scaffold-level assembly were mapped to GCF_000003815.2 chromosome-level assembly via LASTZ (v1.04) whole genome alignment. As a result, we found the chromosomal-level synteny between sea cucumber and Florida lancelet genomes is largely conserved, with even 10 amphioxus chromosomes and sea cucumber LGs showing direct one-to-one orthology (Supplementary Table 35). Last, we compared *B. floridae* genome with our ACs by means of reciprocal blast best hits, and found a very similar pattern than with *A. japonicus*: most amphioxus chromosomes correspond to one AC depicting, whereas AC1, AC2, AC3 and AC6 corresponded to two or three amphioxus chromosomes (Supplementary Table 36). All in all, we conclude that the sea cucumber represents a suitable outgroup lineage to vertebrates.

**4.5.4) Comparison of the 17-AC model with previous inferences.** Last, we compared our 17 ACs with previous models of pre-WGD ancestral chromosomes, including the 17-Tetrad[121], 17-CLG[122] (although it most recently recognised 18 CLGs[123]) and 18-PVC[124] models (Supplementary Tables 37, 38). The gene contents of the 17 Tetrads and 17 CLGs were mapped to our ACs via

unidirectional blast best hit search. The gene content of the 18 PVCs[124] is based on the amphioxus *B. floridae*, so in this case we compared with our *B. belcheri* genes anchored to our ACs via reciprocal blast best hit search. The statistical test of significance follows the same rule as described in section (4.3) wherein a Chi-squared test and FDR correction were used. The detailed gene level homologies are described in Supplementary Table 38 new.

**4.6) Resolving how 2R WGD leads to the extant chromosomal phylogeny of gnathostomes.**

**4.6.1) Chromosomal phylogeny of gnathostomes.** With the homology map between the sea cucumber LGs, the reconstructed ACs (proxied with genes from the amphioxus *B. belcheri*), and sets of gnathostome chromosomes (approximated by one-to-one spotted gar LOCs and chicken chromosomes), we attempted to dissect the chromosomal phylogeny of gnathostomes generated by 2R WGDs. Specifically, we defined homologous gene sets as homologs harboured by chicken, spotted gar and amphioxus (Belcher's lancelet unless specified) with the following requirements: (1) for homologs of chicken and gar in one gene sets, their corresponding chromosome needs to map to a single AC (Supplementary Table 39); (2) at least a total of five different chromosomes (chicken) or LOCs (spotted gar) needs to be covered, which has a maximum value of eight (four in chicken, and four in spotted gar) under the 2R WGD model; (3) gnathostome genes included in one set have the highest BLASTP scores with the amphioxus gene in the same set compared to remaining genes harboured by chicken or gar genomes. These criteria were applied in all phylogenetic analyses unless otherwise specified. To increase the number of homologous gene sets, we additionally supplemented amphioxus genes from Florida lancelet besides Belcher's lancelet. Among all 701 homologous gene sets (Supplementary Table 39), genes of Florida lancelet constitute only 19 homologous gene sets, and these 19 genes are likely unannotated in the genome of *B. belcheri*.

As aforementioned, the spotted gar LOC2.1 and LOC4.2 are co-homologous to chicken chr4.2 and Z. In subsequent analysis, we took advantage of co-homology between chrZ (spotted gar LOC2.1) and chicken chr4.2 (spotted gar LOC4.2) to include more applicable genes. That is, homologous genes on chicken chr4.2 (spotted gar LOC4.2) were used to complement homologous genes on chrZ (LOC2.1) in AC2 and AC11 related analysis given the correspondence between chrZ (LOC2.1) and AC2 or AC11. And homologous genes on chicken chrZ (spotted gar LOC2.1) were used to complement homologous genes on chr4.2 (LOC4.2) in AC3 and AC4 related analysis. For example, 71 chr4.2 genes and three chrZ genes were added into AC3 homologous gene sets (Supplementary Table 39).

If applicable, we added homologous sea cucumber genes into homologous gene sets to supplement phylogenetic information. Different from chicken and gar, we herein did not strictly follow the correspondence between sea cucumber LGs and ACs (Supplementary Table 39). For example, sea cucumber LG01/LG11/LG17 corresponds to AC1. But in homologous gene sets related to AC1, sea cucumber genes could be harboured by LG02 (1 gene) and LG09 (1 gene) in addition to LG01, LG11 and LG17 (a total of 48 homologous gene sets). The rationale is that translocations may occur due to the remote relationship between sea cucumber and gnathostomes and possible misassembly in sea cucumber may also confound the chromosomal linkage. Therefore, although we used a single sea cucumber linkage group name to denote a sea cucumber outgroup LG in the phylogenetic analyses, like “LG01/LG11/LG17” in (Extended Data Figure 5a), we clarify that the sea cucumber genes from other LGs make a small contribution too (Supplementary Table 39).

With homologous gene sets, we built and polished multiple sequence alignments (MSAs). Specifically, Prank v150803 was used to generate MSA with the parameter ‘-F’ to imply

permanent insertions[125]. Since annotation error is pervasive in non-model species[126] (e.g. lamprey), we visualised every MSA in MEGA7 (REF. 127) v7.0.18 and perform a series of manual curation: 1) for tandemly positioned genes in one species but annotated as one gene model in all other species, we interpreted them as artificial gene fission and corrected them back; 2) for alignments consisting of a highly diverged region from one protein, we viewed them as annotation error due to erroneous gene structure and thus discarded this region; 3) for a gene harbouring an alignment gap relative to other homologs, we interpreted it as a missing exon, analysed such region with BLAT and UCSC genome browser[127–129] given the surrounding alignable region of this gene and performed the correction according to annotation tracks of UCSC, NCBI orf-finder or TBLASTN gateways and the latest Ensembl annotation track of the same or closely related species. In total we curated 1,957 sequences from all these species and attached the correct sequences in Supplementary File 4. After this manual curation, gaps and poorly aligned regions were automatically filtered using Gblocks v0.91b with parameters as ‘’-b3=10 -b4=5 -b5=h” specifying a loose criteria, which is generally more superior than the default stringent settings when searching for ML tree based on high divergence alignments[130]. By contrast, for gene level tree analyses with much limited information, we did not implement Gblocks since filtering alignments sometimes leads to a worse result and the more aggressive filtering strategy is, the less reliable trees would be finally obtained[131].

We then performed phylogenetic tree reconstruction. For any MSA, we used ModelFinder with BIC criteria to find the best-fitting evolutionary model and with the parameter ‘-mtree’ to enable an exhaustive search[132]. To infer chromosome level phylogeny, MSAs corresponding to the same AC are concatenated to form a super matrix. Specifically, outgroup genes from sea cucumber or amphioxus were separately concatenated, vertebrate genes from the same chicken or gar

chromosomes were concatenated. Each MSA within the super matrix was defined as a separate partition with dedicated evolutionary models[133]. We concurrently ran RAxML-ng[134] v0.9.0 and IQ-TREE[135] v1.6.12 to infer chromosome level maximum-likelihood (ML) trees. For RAxML-ng, bootstraps were automatically implemented and bootstrap supporting values were calculated with the TBE method[136]. For IQ-TREE, 1000 ultrafast bootstraps were conducted. Finally, by applying a coalescence approach to generate a chromosomal level consensus tree, all bootstrapped MSAs corresponding to the same AC were grouped as the input to Astral-III[137], version 5.6.3.

**4.6.2) Nature of 1R and 2R polyploidy events.** It has been recently proposed that 1R was an autotetraploidization event, and that 2R was an allotetraploidization event[122,124], based on gene retention asymmetries after each WGD event, which is expected to be symmetrical after an autotetraploidization, and notably asymmetrical after an allotetraploization[138–140]. To further consolidate this model, we quantified the asymmetry of paralogous chromosome after either 1R or 2R (Extended Data Figure 5b). The asymmetry in gene retention ratios after 2R ranges between 1:1.84 and 1:21.71 (median 1:2.48), which is significantly higher than that after 1R (between 1:1.00 and 1:1.53 with a median of 1:1.16, Wilcoxon rank sum test $P$=3.4 x $10^{-7}$). Therefore, we conclude that gnathostome genomes have experienced a genome-wide gene retention bias after 2R, while this phenomenon is largely absent after the 1R. So, our chromosome level phylogeny and reconstructed gnathostome karyotypes shaped by 2R also supports that 1R was an autotetraploidization and 2R an allotetraploidization.

Note that our method is novel compared to Simakov et al.[122] and Nakatani et al[124] because our observation is based on chromosome level phylogeny, and this strategy is essential. For example, chicken chr2, 7, 27 and 33 are descendant copies of AC1 (Extended Data Fig. 5a, b). The observed phylogeny for the four paralogous chromosomes is ((chr2, chr27), (chr7, chr33))

(Extended Data Fig. 5a), which, given the gene asymmetries, supports the 2R-allopolyploidy hypothesis. However, if the phylogeny had been ((chr2, chr7), (chr27, chr33)), it would then support a 1R-allopolyploidy event instead, since the size asymmetry will be more profound after 1R than after 2R. So, our chromosome level phylogeny is critical for dissecting the polyploidy modes of 1R and 2R.

**4.6.3) Chromosomal fusion and karyotype evolution before, between and after 2R WGDs.** By following the parsimony rule, we reconstructed how gnathostome karyotype evolved through 2R WGDs (Extended Data Figure 5b). After the first round of WGD, 34 chromosomes were derived from 17 ACs. The correspondence between ACs and back-mutated chicken/gar chromosomes shows signals of eight post-1R fusions, in which one fusion event involved three chromosomes (Supplementary Figure 22). Thus, 34 chromosomes became 25 chromosomes. After the second round of WGD, 50 chromosomes emerged. Six chromosomes, including one copy derived from AC7, AC8, AC9, AC15, AC16 and AC17, respectively, appeared to be lost before the split of chicken and gar because these ACs correspond to 3 modern chromosomal sections, instead of four (Extended Data Figure 5). Therefore, only 44 chromosomes were retained. Four post-2R fusions occurred before the split of chicken and gar lineage, leading to chicken chr2 (spotted gar LOC9LOC11), chicken chr3 (gar LOC1.2LOC16), chicken chr1.4 (spotted gar LOC3.1LOC14.2LOC17) and gar LOC2.2 separately. Therefore, 40 chromosomes were present before the split of chicken and gar lineages. Six chromosomes were then lost specifically in chicken resulting in the aforementioned six gar-specific chromosomal segments.

There are two notable issues: (1) we only showed the evolutionary process from 17 ACs to 50 chromosomes after the second rounds of WGD in Extended Data Figure 5b, since post-2R events were irrelevant with our major focus; (2) the sizes of the chromosomes in Extended Data

Figure 5b were predicted as the total count of retained genes from chicken and gar orthologous chromosomes for post-2R chromosomes, and the total count of retained genes from two gnathostome chromosomes (reconstructed based on chicken and gar) for post-1R chromosomes.

**4.6.4) Comparing the gnathostome chromosomal fusions in our model to previous models.** We evaluated how our pre-1R and post-1R chromosomal fusion events differ from previous literatures. Pre-1R fusions were not formally discussed in the 17-Tetrad model[121] or the 17-CLG model[122]. Post-1R fusions are provided as tetrad pairs[121], CLG pairs[122,123], or PVCs pairs[124] from the original articles. Mapping our 17 ACs to the 17 Tetrads[121], the 17 CLGs[122] and the recent 18 PVCs[124] (Supplementary Tables 37, 38, 41), confirms most of the identified post-1R fusions events, except the one pairwise fusion events found between CGLI and CLGQ in Simakov et al.[122,123] and Pvc8 and Pvc9 in Nakatani et al.[124], which corresponds to a single pre-1R fusion in our model, giving rise to AC3 (Supplementary Table 41).

**4.6.5) Simulating pairwise post-1R fusions.** Under our model of 17 ACs, we infer eight post-1R fusion events. However, under the pairwise "post-1R fusion" model, AC3 does not represent a pre-WGD fused chromosome but instead represents two separate pre-WGD chromosomes, CLGI and CLGQ in Simakov's model[122,123] and Pvc8 and Pvc9 in Nakatani's model[124] that happened to have fused twice after 1R (Supplementary Figure 23). In this scenario, 10 post-1R fusion events are inferred, including thus a post-1R pairwise fusion. To estimate the likelihood of a pairwise fusion, we ran a simulation of 10 random post-1R fusions and recorded the number of pairwise fusions occurring in such a simulation. We performed three technical replicates with each replicate including 10,000 independent simulations (Supplementary Table 40). We found that around 30% of the simulated cases implied at least one event of two post-1R pairwise fusions (involving two of the same ohnologous chromosome pairs), and at least two cases of four pairwise fusions

(involving four pairs of ohnologous chromosomes) in approximately 3% of cases (Supplementary Table 40).

**4.7) Phylogenetic analyses of cyclostome and gnathostome genes.**

To decipher whether cyclostomes and gnathostomes share WGD(s), we anchored cyclostome genes into the aforementioned phylogenetic trees of gnathostomes (see section 4.6.1). That is, we incorporated genes from shark, hagfish and lamprey based on homologous amphioxus genes, built MSAs and reconstructed maximum likelihood (ML) trees (Supplementary Table 42). To increase phylogenetic information, we also added homologous Florida lancelet genes of all vertebrate genes in a homologous gene set. For each single MSA, we separately and repeatedly run RAxML-ng and IQ-TREE for 10 times using different seed numbers to increase the power of ML tree search[141]. For the 20 obtained ML trees for each MSA, we used RAxML-ng to re-evaluate their likelihoods and chose the best tree as the final tree for a MSA.

To increase the number of applicable trees, we performed three separate analyses by only incorporating hagfish genes, lamprey genes, or genes of hagfish and lamprey, respectively (Supplementary Files 5-8). For each separate trial, shark genes were included as a positive control given their stable topological positions.

To choose high-quality phylogenetic trees, we implemented the ETE toolkit[142] (v3.1.3) to require that all bootstrap values in a single gene tree should be greater than 70%. The only exception is the monophyletic clade consisting of a shark gene, a gar gene and a chicken gene, for which the cutoff was relaxed to 60%. The reason is due to possible incomplete lineage sorting wherein shark genes may cluster more closely with chicken or gar genes. However, the internal topology of this clade did not affect the position of cyclostome genes.

We finally manually examined all trees to require that the topology of chicken and gar genes were consistent with the chromosome level tree. We required that the phylogenetic relationships of chicken genes and gar genes exactly recapitulated the chromosome level trees depicted in aforementioned gnathostome chromosome level tree (Extended Data Figure 5a), since only these trees provide information to evaluate the positions of cyclostome genes relative to two rounds of WGD.

The possible position of tested genes from each model follows the example presented in Fig. 4a of the main text. For each tree, we classified it into shared 1R-compatible or -incompatible first. If it was classified as shared 1R-compatible, we further classified it into shared 2R-compatible or -incompatible. Note that unlike the cases where there is a one-to-four correspondence in Fig. 4a, sometimes one AC gene corresponds to three chicken or gar genes (Extended Data Figure 5a), in which case we could not distinguish between shared 2R-compatible or incompatible. These cases would be only used to infer shared 1R compatibility, but not 2R. Last, when multiple hagfish genes or sea lamprey genes were included simultaneously, we required they support the same compatibility scenario when assessing 1R compatibility and 2R compatibility.

**4.8) Further supra-scaffolding of the 2018-version of the sea lamprey germline genome assembly.**

We performed most of our analysis with the 2018 version of sea lamprey germline genome[143], before the most recent chromosome-level assembly was published[144] and thus free from embargo. To further increase the continuity of the scaffold-level sea lamprey assembly[143], we first took advantage of the closely related Pacific lamprey linkage group (LG) information published

together with the same genome assembly by Smith et al.[143]. We extracted the orthologous mapping information between the two species provided by Smith et al.[143], and in those cases in which more than one sea lamprey scaffolds were mapped to the same Pacific lamprey LG, and when they corresponded to the same AC, we interpreted such signal as incomplete assembly of one chromosome and manually fused them, which was represented as the scaffold name of the larger scaffold (Supplementary Table 48). Once the chromosome-level assembly was published[144], a short time before our study was submitted, we used to confirm 17 out 18 cases detected in our supra-scaffolding (Supplementary Figure 24).

**4.9) Investigating whether the cyclostome lineage shared gnathostome pre-1R and post-1R fusions.**

In our analysis, we predicted four pre-1R fusions (Supplementary Figure 15; Supplementary Table 32). For instance, AC1 emerged after a pre-1R fusion of ancestral chordate linkage groups that correspond to sea cucumber LG01, LG11 and LG17 (Supplementary Figure 17) and *B. floridae* chromosomes BFL16, BFL10 and BFL18, respectively (Supplementary Table 35). We thus analysed homologous relationships between sea cucumber LGs and sea lamprey improved supra-scaffolds and hagfish Hi-C clusters. For example, if any one lamprey scaffold or hagfish cluster is homologous to all LG01, LG11 and LG17 of the sea cucumber assembly, we deemed that a pre-1R fusion of sea cucumber LG01/LG11/LG17 is shared by sea lamprey genome and/or hagfish. We found signals for three in the sea lamprey and two in the hagfish, out of 4 of the pre-1R fusion events detected (Supplementary Figures 25, 26). This is expected since cyclostomes and gnathostomes diverged after 1R[121–124,145,146].

As aforementioned, we have also predicted eight post-1R fusions in gnathostome genomes (Supplementary Figure 22; Supplementary Table 41). We then checked whether the lamprey genome holds signals of any of these fusion events, because that would indicate that 2R also predated the divergence of cyclostome-gnathostome split. We found that scaf_00006 is a descendant copy of AC2 and AC11 (Extended Data Fig. 6a). However, we didn't find multiple copies (Extended Data Fig. 6a), which would be expected if this happened after 1R but before the cyclostome and gnathostome divergence and therefore before the cyclostome whole-genome triplication. Moreover, we also found that the two sets of homologous genes have a clear chromosomal boundary on scaf_0006 (Extended Data Fig. 6a). Such a pattern is unexpected if scaf_00006 emerged by an ancient post-1R fusion as intrachromosomal rearrangements should blur the boundary of the two ancient sets of homologous genes, derived each from AC2 and AC11. Therefore, it is more likely that sea lamprey scaf_00006 is the result of a recent fusion of two cyclostome chromosomes that are respectively descendant copies of AC2 and AC11. All in all, we concluded that all gnathostome post-1R fusions were not shared by sea lamprey genome.

In addition to the scaffold-level sea lamprey assembly reported by Smith et al.[143], we also corroborated the lack of reliable signals of any of the eight post-1R fusions in the most recent chromosome-level genome assembly of *P. marinus*[144] (Supplementary Figure 27).

**4.10) Gene retention profile-based analyses.**

**4.10.1) Definition of gene retention profile and overlapping ratio.** To enable phylogenetic analyses between cyclostomes and ACs, we implemented a novel summary statistic, i.e., gene retention profile. It was defined as a vector listing the presence or absence of homologous genes on a vertebrate chromosome for all genes of the corresponding AC. Since an AC contains genes

harboured by different Belcher's lancelet scaffolds, the order information of genes on an AC is unknown. So, when drawing the gene retention profile of a chromosome, the genes of one AC were firstly randomly ranked by the lancelet scaffold it originally belongs to and then by the real gene order within that scaffold (Supplementary Files 10, 11). Certainly, the order is irrelevant for all phylogenetic analyses, which were only based on presence or absence information. For two chromosomes corresponding to the same AC, the overlapping ratio was calculated based on two gene retention profiles or vectors (Figure 4b). To enhance robustness, we required at least 20 retained genes on descendent chromosomes from a given AC before calculation of the overlapping ratio; since it harboured only 20 genes, AC17 was excluded from the analysis.

To make overlapping ratio comparable between gnathostomes and cyclostomes, we corrected a bias inherited during reconstruction of the gene content of ACs based on amphioxus genes: genes encoded by an AC must share homologs in corresponding homologous chromosomes of both chicken and gar, while hagfish and lamprey genes were not considered. Therefore, the genes on ACs should be biassed towards gnathostome-specifically retained genes. We corrected the bias by requiring the genes on ACs to share homologous genes in corresponding chromosomes of both hagfish and lamprey. Before the correction, 4,325 out of 5,045 genes on all AC1-16 were retained in either hagfish or sea lamprey. After the correction, 3,081 genes were retained and used in cyclostome related analyses. Note, the cut-off (0.15 in Figure 4c-e) was derived based on all 5,045 genes on ACs. If only 3,081 genes were used, the cut-off (0.15) remained robust.

**4.10.2) Threshold of overlapping ratio to discriminate homologous chromosomes.** We generated the random expectation of *OR*s by artificially splitting a chromosome into two parts and calculating the ratios. In other words, for a vertebrate chromosome corresponding to one AC, we ordered the homologous genes by their actual positions on the chromosome and manually split

these homologous genes into two parts on all possible gene boundaries. Then, if each part harbours at least 20 retained genes, we calculated their *OR*s. Therefore, for each chromosome and the corresponding AC, there would be multiple *OR*s from random splits, the maximum value of which was recorded as the final ratio for this chromosome. Two rationales were invoked in this simulation: 1) we could not randomly sample two chromosomes since they may be duplicates derived from one AC; 2) we chose the maximum rather than median value in order to be conservative, i.e., to generate an upper-bound estimation of overlapping ratios of two random chromosomal segments. Finally, given each chromosome and the corresponding AC, there are a total of 49, 45, 48 and 47 simulated split results in chicken, gar, hagfish and sea lamprey genome, respectively (Supplementary Table 43).

By comparing the distribution of overlapping ratios for artificially split chromosome pairs and bona fide paralogous chromosome pairs in gnathostomes, we reasoned that OR=0.15 should serve as a good threshold. Only 3 of 94 (3.2%) random splits from either chicken or gar genome have an overlapping ratio > 0.15 (Supplementary Table 43). So, the specificity of our methodology to identify paralogous chromosome pairs has a lower bond of 96.8%.

**4.10.3) Application of overlapping ratio.** We performed a series of analyses based on overlapping ratios: (1) we identified two chromosomes as paralogous chromosomes when their overlapping ratio exceeds the cut-off of 0.15, which was specified based on the comparison of *OR* values between randomly split chromosome pairs and that of real paralogous chromosome pairs generated by WGD from chicken and gar data (Figure 4c, c); (2) we identified the orthologous cyclostomes chromosomes as a pair of hagfish and lamprey chromosomes that formed a single clade in the clustering of chromosomes derived from the same AC based on gene retention profile (Supplementary Files 10, 11). For (2), the distance was equal to one minus overlapping ratio and

the clustering was then conducted based on the distance matrix of these gene retention profiles with a "ward.D2" method embedded in the R package `pheatmap` (v1.0.12) using R v3.6.0 (Supplementary Tables 44, 45).

**4.10.4) Splitting three hagfish chromosomes identified further cyclostome orthology.** We found that hagfish cluster9 (corresponding to AC10), cluster10 (AC14) and cluster14 (AC16) show peculiar overlapping ratio patterns after manual split and examined why. Specifically, during the previous chromosomal split analyses in hagfish and sea lamprey, we noticed that two parts in these three hagfish clusters show overlapping ratios higher than 0.15 (Supplementary Table 43), suggesting chromosomal fusion of two paralogous chromosomes in hagfish evolution. We therefore manually split these hagfish clusters to examine whether two parts would separately cluster with two lamprey scaffolds (Supplementary Figure 28). To determine the boundary, we first obtained all homologous genes on the hagfish cluster that corresponds to a specific AC and sorted them according to their actual position. We then exhaustively scan all paralogs to search for a boundary to maximise the total number of shared retained genes between the two hagfish parts. We finally split the hagfish cluster on this position and redo the overlapping ratio-based clustering (Supplementary Figure 28).

**4.10.5) Simulating the decay of overlapping ratio after a third round of WGD.** We expected that the overlapping ratio of any sister chromosome pairs drops with the increase of the number of WGD(s) since only a proportion of duplicates could be retained and retained duplicates could be scattered to more paralogous chromosomes. To make a more quantitative inference, we made a mathematical deduction (Supplementary Figure 29) based on two simple but reasonable assumptions: (1) the gene loss and gene retention are symmetrical; (2) all chromosomes have the

same duplicate gene retention rate after a WGD. Specifically, for any paralogous chromosome pair, i.e., chr1 and chr2, their overlapping ratio (*OR*) is defined as,

$$OR(raw) = \frac{a}{min\ (a+b, a+c)}$$

After an additional round of WGD with duplicate gene retention rate $p$, two chromosomes became four chromosomes, which are named as chr1.1, chr1.2, chr2.1 and chr2.2. The *OR* between sister pairs (e.g., chr1.1 and chr1.2) is only dependent on the duplicate gene retention rate:

$$OR(new.1) = \frac{2p}{1+p}$$

while the *OR* between homologous chromosome pairs not directly generated by WGD (e.g., chr1.1 and chr2.1, chr1.2 and chr2.1) is

$$OR(new.2) = \frac{1+p}{2} * OR(raw)$$

Now, we would like to know the impact of a third round of WGD on *OR* among paralogous chromosomes. After two rounds of WGD, a maximum of four paralogous chromosomes are generated and the average *OR* for two out of four paralogous chromosomes is defined as

$$OR(2R)$$

After a third round of WGD, four paralogous chromosomes became eight paralogous chromosomes. And there are in total 28 different combinations of two paralogous chromosome pairs from 8 paralogous chromosomes, of which 4 chromosome pairs were directly generated by the third round of WGD and the remaining 24 not directly generated by the third round of WGD. For the former 4 sister chromosome pairs, their OR again is

$$OR(3R.1) = \frac{2p}{1+p}$$

For the later 24 pairs, their overlapped ratio is

$$OR(3R.2) = \frac{1+p}{2} * OR(2R)$$

Therefore, the average *OR* between any 2 out of 8 paralogous chromosomes after the third round of WGD is

$$OR(3R) = \frac{4}{28} * OR(3R.1) + \frac{24}{28} * OR(3R.2) = \frac{4}{28} * \frac{2p}{1+p} + \frac{24}{28} * \frac{1+p}{2} * OR(2R)$$

We then approximated *OR(2R)* in cyclostomes with the median *OR* of paralogous chromosomes contributed by 2R WGD (0.52) in gnathostomes (Supplementary Table 46). That is,

$$OR(2R) = 0.52$$

For teleost which was subject to the third round of WGD about 320-350 million years (myr) ago, the duplicate gene retention rate in extant species were 0.15-0.20 (REFs. [147–149]). We used this range to approximate the value of *p* for the third round of cyclostome WGD, which likely occurred before the split of hagfish and lamprey, that is, earlier than 470 mya. Performing in silico simulation with a reasonable p ranging between 0.08 and 0.30, we found that the mean *OR* after the third round of WGD is generally between 0.26 and 0.36. That is,

$$OR(3R) \sim (0.26, 0.36)$$

Thus, as expected, after a third round of WGD, the mean *OR* of paralogous chromosomes would further drop. Presumably, triplication would lead to a similar drop.

Finally, we conducted a direct test of our aforementioned conclusion using the zebrafish genome, which underwent the teleost-specific third round of WGD. Our findings are aligned with our model, as evidenced by the zebrafish paralogous chromosome pairs displaying a median OR of 0.28 (Supplementary Figure 29). This value is much lower compared to the median OR values of the chicken and spotted gar genomes, which are 0.49 and 0.54, respectively. In contrast, it is much similar to the observed median OR in cyclostome species (0.30, Supplementary Table 47), consistent with a larger polyploid event than a duplication, such as the suggested triplication event.

**4.11) Estimating the chance of paralogous chromosomal fusions in hagfish evolution.**

We wondered how frequent paralogous chromosomal fusions occurred in hagfish lineage since such events confound the analyses of overlapping ratios. For example, both hagfish cluster1 and cluster6 correspond to AC10, and cluster1 is paralogous to cluster6 on the AC10 derived regions according to their overlapping ratio. We firstly randomly shuffled the correspondence between ACs and hagfish clusters. We then counted the cases where two paralogous parts were shuffled to the same hagfish cluster. For example, if the cluster1-AC10 part and cluter6-AC10 part were shuffled into one cluster, it was counted as one fusion event between paralogous chromosomes. We performed two replicates of simulations with each replication consisting of 10,000 simulations (Supplementary Figure 30). The interquartile range (IQR) of paralogous chromosome fusion events were estimated to be between 3 and 6.

## 5. REGULATORY EVOLUTION

### 5.1) Ohnologs

**5.1.1) Defining ohnologs.** Because the homology between vertebrate chromosomes and ACs have already been established (Extended Data Figure 5a), a group of genes locating on different chromosomes but sharing the same homologous gene on the corresponding AC was considered an ohnolog family. To minimize the confounding effects from tandem duplicated genes, we required the count of homologous genes should be no more than one plus the count of unique chromosomes where these genes are located. It means for one ohnolog family, at most one tandem duplicate gene may be included. As a result, 2,137 hagfish and 3,594 chicken ohnologs were identified (Supplementary Tables 48, 49).

**5.1.2) Gene Ontology of ohnologs.** We mapped hagfish and chicken ohnologs to human genes and performed GO enrichment analysis with human homologs since human genes have far more comprehensive ontology annotation than hagfish and chicken. For 2,137 ohnologs and all 16,473 protein coding genes in hagfish, we identified 959 and 8,293 reciprocal best hits in the human genome, respectively. For 3,594 ohnologs and all 18,346 protein protein coding genes in the chicken genome, we identified 3,288 and 12,875 reciprocal best hits, respectively. The larger gene list served as the statistical background. Functional enrichment was examined with the Metascape[150] online tool (v3.5). We used the 959 human orthologs of hagfish ohnologs and randomly sampled 2,999 genes (as Metascape has a limit of 3,000) from a total of 3,594 chicken orthologs. We performed GO (Biological Process only) enrichment analysis against all genes of the two species. The top 20 enriched functions of ohnologs of two species and their parental GO terms have been shown in Extended Data Figure 8a,b. Semantic similarity score[151] between the two sets of enriched GO terms was 0.694, higher than random expectation (0.20~0.45)[152].

Furthermore, genes were annotated as either developmental ohnologs, non-developmental ohnologs or non-ohnologous genes according to their GO terms annotated by PANTHER[153] v17.0.

**5.1.3) Fate of ohnologs after WGD.** As aforementioned, hagfish transcriptome data of nine somatic tissues were generated in this study. Chicken transcriptome data of nine somatic tissues were retrieved from public sources[154]. Gene level transcriptomic quantification was performed with RSEM[155] software (version 1.3.1) using STAR[156] mapping method (version 2.6.1d). Following previous practice[4], we normalized transcriptome data of different tissues with the quantile method by in-house scripts (code provided in Supplementary File 12). After normalization, TPM (transcripts per million) > 5 is used as threshold to binarize a gene state as either 'expressed' or 'not expressed'. Tissue specificity is quantitatively described by Tau index[157]; we chose 0.9 value as a cut-off to define tissue specific genes.

Herein, we only analysed ohnolog families containing exactly two ohnologs. For an ohnolog pair, we required both genes to be expressed in at least one tissue of tissues assayed in this study for the hagfish or retrieved from public databases for the chicken[154]. Since we did not have information about expressional domains of orthologous amphioxus genes in homologous tissues, we followed a different strategy than that of Marlétaz et al.[4] to define the expressional fates of ohnologous genes. Ohnolog pairs were classified into three classes based on their expressional states across tissues[4] (Supplementary Figure 31): (i) potential redundancy, if the two ohnologs are expressed in the same set of tissues; (ii) potential subfunctionalization, if both ohnologs are each expressed in a tissue not shared with the other. In other words, each of them has tissue-specific expression domains; (iii) potential specializatoin, if one ohnolog has a reduced set of expression domains contained in a larger set of tissues in which the other ohnolog is expressed. Gene families within 'specialization' can be further defined as either 'potential strong specialization' when the

ohnolog with the narrower expression pattern is transcribed in < 40% of the domains than the ohnolog with the broader expression pattern; or 'potential mild specialization', when the ohnolog with the narrower expression pattern is transcribed in ≥ 40% of domains than the ohnolog with the broader expression pattern.

**5.2) ATAC-seq analysis**

**5.2.3) ATAC-seq of hagfish embryos.** Embryonic samples of the hagfish are extremely rare[158]. In this study, we collected two embryos of the inshore hagfish *E. burgeri*, at stages Dean 45 (collected in 2018) and 53 (collected in 2017) (REF. 159; Supplementary Figure 32), and performed ATAC-seq experiments following previous descriptions[160,161] with slight variations. Embryos were divided into two parts, anterior and posterior halves, at the level of the somite pair 27 (Supplementary Figure 32), in order to gain positional information for future projects.

Nuclei were extracted separately from each part using 2 mL glass bouncers in ice-cold PBS and lysed in cold lysis buffer (10 mM Tris pH 7.4, 10 mM NaCl, 3 mM MgCl2 and 0.1% Igepal). Each pool was further divided into two to obtain data from technical replicates (given the lack of biological replication). Transposition reaction was done on ~50,000 nuclei per sample and per replicate (total of 200,000 cells per embryo), with Tn5 enzyme (Illumina Nextera DNA Library Prep Kit, #FC-121-1030), during 5 min at 37 ºC. DNA was purified using a QIAGEN MinElute kit following manufacturer instructions. Number of PCR cycles per sample were first determined as previously described(Buenrostro et al., 2013) using 1 uL of the Tn5'ed chromatin, and then a final PCR reaction with the rest of the chromatin was performed for each sample, with the following number of cycles: 7 for the embryo at stage 45, and 8 cycles for the embryo at stage 53. PCRs were performed with the NEBNext High-Fidelity 2x PCR Master Mix (New England

Biolabs, #M0541), using primers Ad1F and Different sets of primers were used to multiplex the libraries, and obtained from Buenrostro et al.[161]. Primer combinations per sample used are as follows:

- stage 45 anterior 1: Ad2.5R; stage 45 anterior 2: Ad2.7R;
- stage 45 posterior 1: Ad2.6R; stage 45 posterior 2: Ad2.11R;
- stage 53 anterior 1:Ad2.3R; stage 53 anterior 2: Ad2.9R;
- stage 53 posterior 1, Ad2.4R; stage 53 posterior 2, Ad2.10R.

The resulting multiplexed libraries were checked in an Agilent 2100 BioAnalyzer using High Sensitivity DNA chips (nucleosome pattern was observed in the profiles) and sequenced in four lanes (2 lanes per embryo) of an Illumina HiSeq 4000 platform, at the Beijing Genome Institute (BGI).

**5.2.2) ATAC-seq data of amphioxus, zebrafish, medaka, chicken, and mouse.** For the ATAC-seq analysis of species except for hagfish, we used published ATAC-seq datasets (Accession ID: GSE106428 for amphioxus and zebrafish[4]; DRA006971 for medaka, chicken, and mouse[162] (Supplementary Table 51). While there are two replicate data for hagfish, amphioxus, and zebrafish, there are three replicate data for each sample for medaka, chicken, and mouse ATAC-seq data. In order to minimise analytical biases derived from the different numbers of the replicate ATAC-seq data, we removed the ATAC-seq data with the lowest number of aligned ATAC-seq reads for medaka, chicken, and mouse, so that we can use the same number of replicate data across different species data in the ATAC-seq analysis.

**5.2.3) ATAC-seq alignment and peak calling.** Adaptor removal of raw paired-end reads were performed by using NGmerge[163] (version 0.3) with the following parameters: -a -e 30. The filtered

reads were aligned to species-specific reference genomes (this study for *E. burgeri*, BraLan2 for *Branchiostoma lanceolatum*[4], GRCz11 for *Danio rerio*[164], ASM223467v1 for *Oryzias latipes*[165], GRCg6a for *Gallus gallus (International Chicken Genome Sequencing Consortium 2004)*, GRCm38 for *Mus musculus*[166]) by using bowtie2 (version 2.4.2) (REF. 13) with the following parameters: -k 4 -X 2000 --sensitive. These genomes were downloaded from the Ensembl database (release 101)[167]. Picard (version 2.23.8; http://broadinstitute.github.io/picard/index.html) was used to remove duplicate reads from properly paired aligned reads, which had been filtered by using SAMtools[168] (version 1.10). For peak calling, we basically followed the pipeline described in Marlétaz et al.[4]. First, to deplete ATAC-seq reads derived from the transposition events that happened on both sides of a nucleosome, we removed the properly paired reads whose mate is more than 120 bp for identification of accessible chromatin reginos (ACRs). Then, the start position of every aligned ATAC-seq read was corrected to account for the 9-bp insert between the adaptors, which was introduced by Tn5 transposase[161], and the single 5′-most base of each read was retained to obtain single-base resolution. For each sample, the data for the two replicates were pooled into a single dataset. For the pooled data and those for each replicate data, peak calling was performed by using MACS[169] (version 2.2.7.1) with the following parameters: callpeak --nomodel --keepdup 1 --llocal 10000 --extsize 74 --shift -37 -p 0.07. Finally, on the basis of replicate information, we obtained the high confidence ACRs by using the IDR framework[170] (idr 2.0.4 with the following parameter: -i 0.1). For each sample, we identified more than 130,000 accessible chromatin regions (ACRs) as putative regulatory regions (Supplementary Figure 33a). It should be noted that identified hagfish ACRs would not have any information on biological variation, as we have no biological replicates but only technical replicates in our ATAC-seq data because of the extreme difficulty in hagfish sampling, especially of embryonic material.

For newly generated hagfish ATAC-seq data, we confirmed that the determined accessible chromatin landscapes were reliable by several analyses. First, we examined the fragment sizes of ATAC-seq for each sample and confirmed the periodic distributions corresponding to integer multiples of nucleosomes (Supplementary Figure 33b). Second, quantifying the fraction of all aligned reads in the ACRs (FRiP scores, which is a genome-wide, quality metric of the signal-to-background ratio[171]), we confirmed a high signal-to-background ratio of all ATAC-seq data (Supplementary Figure 33c). In addition, identified ACRs were not distributed randomly through the genome but significantly enriched at promoter regions of transcription start sites (TSSs) (Supplementary Table 52). Moreover, the ATAC-seq signal intensities at ACRs were robustly reproduced between the two technical replicates (Supplementary Figure 33d). Collectively, the obtained results indicate that our embryonic ATAC-seq data provide a reliable accessible chromatin landscape of the hagfish genome.

**5.2.4) Mapping ACRs to genes.** To map ACRs to the putative target genes, we defined the *cis*-regulatory region of each gene according to the 'Genomic Regions Enrichment of Annotations Tool' (GREAT[172]). First, each gene is assigned a proximal regulatory region that extends 5 kb upstream and 1 kb downstream of the TSS, or until another TSS is found. Next, the proximal region is extended up to 1 Mbp in both directions, until another proximal region is encountered. This extended region was defined as the *cis*-regulatory regions of the gene. ACRs falling into the cis-regulatory region of one gene were considered to be related to its transcriptional regulation. We classified ACR-associated hagfish genes into three different groups: developmental ohnologs, non-developmental ohnologs, and non-ohnologs (see sections 5.1.1 and 5.1.2 for the definitions). Considering the length of GREAT regions are different across the groups, we also normalised the

GREAT length to examine the ACR density of hagfish genes with different roles in development (Extended Data Figure 8e).

**5.2.5) The genomic distribution of ACRs.** To examine the genomic distribution of ACRs in each species, all the ACRs at all the samples were consolidated into a single list. In the merged list, ACRs overlapping each other were combined into a single ACR. By using bedtools[23] (version 2.29.2) and the longest transcript for each gene, these ACRs were classified into four groups: (1) ACRs in promoter regions were defined as those overlapping with regions between 1 kbp upstream and 500 bp downstream of all TSSs of genes; (2) ACRs in proximal regulatory regions were defined as those overlapping with regions between 5 kb upstream and 1 kb downstream of all the TSSs but not overlapping with promoters; (3) ACRs in exons were defined as those overlapping with exons of genes but not overlapping with proximal regions; (4) distal ACRs were defined as those not in groups 1–3. In this analysis, we used the gene sets retrieved from the Ensembl database[167] except for hagfish. For comparing the genomic distributions of ACRs relative to the TSSs of genes across species, the raw distances between ACRs and the closest TSSs are used for Figure 5 and Extended Data Figure 8d; distances were normalized by the average length of intergenic regions for Extended Data Figure 8c.

# 6. DATING WGD EVENTS AND ASSESSING THEIR MORPHOLOGICAL EFFECT

## 6.1) Background.

WGD events have undoubtedly contributed to the expansion of gene families underpinning key developmental processes, but they have also been implicated causally in the evolution of the vertebrate body plan[146,173–176]. The relative timing of vertebrate WGD events has long been thought to coincide with dramatic increases or bursts in phenotypic innovation[173,177]. Testing such hypotheses of causality requires both an estimate of the absolute (geological) timing of the event and a measure of phenotypic diversity (disparity) pre- and post-WGD. The timing of a WGD event can be constrained relative to the timing of lineage divergence events before and after a WGD event, however, this approach is unnecessarily imprecise when applied to events that occur on long evolutionary branches, as here. Instead, molecular clock methods can be applied to directly date WGD events by exploiting the signal of paralogy in gene families where ohnologs have been retained[178,179]. Molecular clock analyses based on gene trees can be calibrated in the same way as species trees, using temporal constraints on the timing of speciation events within gene trees. This approach combines information from the fossil record with Bayesian MCMC methods to estimate probabilistically the age of a gene or genome duplication event. Existing applications have yielded precise estimates for the timing of WGD events in deep time[178,180,181].

WGD events in vertebrates have been inferred on important branches on the vertebrate tree with respect to the key evolutionary innovations including stem-vertebrates, stem-gnathostomes and stem-cyclostomes. Fossil evidence demonstrates that at the very least in the stem-gnathostomes phenotypic innovation played out over a protracted time[71,182]. If WGD events were causal to developmental and phenotypic innovation on these lineages, we should anticipate that

they occurred prior to the appearance of key phenotypes. Alternatively, if the relationship between WGD events, developmental and phenotypic innovation is complementary, WGD would occur after the appearance of these characteristics[182]. Here, we attempt to test these competing hypotheses by establishing the timing of WGD events relative to phenotypic innovation manifest as phenotypic disparity. Phenotypic disparity describes the variance in form between species and lineages[183]. Estimates of disparity can be obtained from living and fossil species and, in a phylogenetic framework, can be used to contrast patterns of phenotypic diversity before and after WGD events[180,184–186]. With that in mind, here we employ Bayesian divergence time estimation to place these genome duplication events temporally, relating this to the evolution of phenotypic disparity within the clade.

**6.2) Dating Whole Genome Duplications**

The age of the 1R duplication event is inferred to be 535.3-524.8 Ma (early Cambrian; Figure 2, Supplementary Figure 34). Across both subtrees, the age of crown vertebrates was estimated at 510.5-506.1 Ma (middle Cambrian), suggesting a lag of 14.3-29.2 Myr between the WGD event and the divergence of crown vertebrates. The age of the 2R event was estimated at 498.4-485.2 Ma (late Cambrian-earliest Ordovician) across both subtrees, with the age of the crown gnathostomes between 450.1-445.1 Ma (Katian, Late Ordovician; Figure 2, Supplementary Figure 34), suggesting a longer lag of between 35.1-53.3 Myr. The CR event is dated to 500.07-491.97 (late Cambrian), 23.4-36.5 Myr prior to the 468.5-463.5 Ma (Darriwilian, Middle Ordovician) divergence of crown-cyclostomes.

**6.3) Phenotypic disparity.**

The phylomorphospace exhibits clustering of clades along both axes, as anticipated, though with stem representatives often plotting intermediate of their respective crown-clades and their nearest living clades. For example, stem-cyclostomes occupy a position within the morphospace that is intermediate of invertebrate deuterostomes and crown-cyclostomes. Similarly, ostracoderms (stem-gnathostomes) are positioned intermediate of cyclostomes and jawed vertebrates, i.e. placoderms+crown gnathostomes (Figure 5a).

Generally, the occupation of morphospace exhibits a strong phylogenetic structure, with clades occupying distinct regions of morphospace. However, placoderms are nested entirely among the chondrichthyan and osteichthyan total groups and, similarly, thelodonts nest within pteraspidomorphs.

Each duplication coincides with an increase in overall disparity and the exploration of a novel region of morphospace (Figure 5b), but the majority of vertebrate disparity emerged subsequent to the 2R WGD event – between 88-97% of the morphospace encompassed by a vertebrate convex hull is attributable to descendants of the 2R event.

**6.4) Discussion.**

Here we have addressed a well-studied problem in phylogenetics, regarding the phylogenetic position of the 2R event in vertebrate evolution. Our high-throughput approach considers all gene families, to incorporate as much evidence as possible without focusing on selected individual gene families. We expect inferred gene duplication events from OrthoFinder to be accurate, because it has some *a priori* expectations of gene tree errors, which it corrects for while predicting duplications[187]. This avoids inferring very large numbers of losses for individual gene families,

because in these cases, a gene tree reconstruction error is likely to be a better explanation for the inferred tree. Further, we only report duplication events from OrthoFinder that have greater than 50% support. Here support means the number of species that we would expect to have both duplicates present as paralogs, without loss events. This means that gene loss is allowed, but duplication events followed by extensive loss would be ignored, because there is a distinct possibility that these patterns represent the consequence of a phylogenetic, gene-tree reconstruction, error. This would further reduce the effect of stochastic gene-tree reconstruction error, at the expense of some correctly inferred gene trees, but this strategy has the advantage of being conservative, only identifying a WGD when substantial, consistent data is available. As a measure to validate our approach we considered whether it robustly and correctly identified uncontroversial vertebrate WGD, i.e. the 1R and 3R (teleost) duplication events, which are here used as positive controls.

In this analysis, we do not distinguish between whole genome duplication and other forms of large-scale gene duplication, and a large-scale duplication does not necessarily correspond to a WGD event. Whole genome duplications appear to be exceptional events in animals, although WGDs have been identified in diverse lineages[188–190]. We are therefore cautious in making conclusions about the potential for WGDs to have happened along branches where they are not expected, based on our results only. However, the evidence we present strongly suggests the 2R duplication happened after the divergence of gnathostomes and cyclostomes. In addition, we identify a triplication event in cyclostomes. The three duplications we identify close to the vertebrate root (1R, 2R and CR) lead to potentially confusing gene-tree patterns, and we argue that these confusing patterns underpinned erroneous suggestions that 2R occurred in the stem vertebrate lineage, or not at all.

We present the first estimates for the timing of the three genome duplication events associated with early vertebrate evolution (Figure 2). Compared to previous estimates, our results represent a significant advancement in that (i) they are derived through integration of palaeontological and molecular evidence, and (ii) they simultaneously estimate the age of the WGD and speciation events. The 1R event occurred within 14.3-29.2 million years before the divergence of crown vertebrates. Species which underwent the 1R event but not the subsequent 2R event, i.e. cyclostomes, are not morphologically diverse even though they have experienced their own WGD events and their lineage is of equal longevity.

The timing of the 2R event indicates that it occurred very early within the gnathostome stem-lineage. This leaves a considerable time lag between the 2R WGD event and the divergence of crown gnathostomes. Such extensive lag periods have also been observed in salmonids, in both the seed plant and flowering plant WGD events, and marshalled in evidence against a causal relationship between WGD and macroevolutionary outcomes[178,179]. However, the 2R duplication event also appears to give rise to the largest increase in morphospace occupation in vertebrates, with the descendants of the event representing the vast majority of vertebrate disparity. Furthermore, the timing of the 2R event at the base of the gnathostome stem-lineage indicates that it occurred before the sequential addition of gnathostome characters revealed by fossil stem-gnathostomes[182,191]. Our lower estimate for the age of the 2R event is older than the earliest unequivocal ostracoderm fossils[191–193]. This indicates that all ostracoderms are descendants of the 2R event. Consequently, the emergence of gnathostome disparity was neither explosive nor delayed. Rather, phenotypic characters were acquired along the gnathostome stem during the protracted period between WGD and crown-gnathostome divergence. The timing of the 2R event, early within the gnathostome stem-lineage, also suggests that WGDs do not confer extinction

resistance in any material sense[194]. This observation is particularly significant since the link between WGD and extinction resistance was based in large part on the predicted relationship between the 2R event and the extensive gnathostome stem-lineage diversity[194].

While both crown-vertebrate lineages (cyclostome and gnathostome) that descended from the 1R event are of equal antiquity and underwent subsequent, independent, WGD events (2R, CR), there is a stark imbalance in both species diversity and phenotypic disparity. The CR and 2R events occurred early in the history of their respective lineages. However, unlike the 2R event, the CR event is not associated with a substantive increase in disparity and diversity. Thus, the evolutionary consequences of WGD are not equal, suggesting that 2R in itself is not sufficient to explain phenotypic innovation in early vertebrate evolution. This interpretation assumes that 2R is an autopolyploidy event and, yet, it has recently been reinterpreted as an allopolyploidy event [122,124,195]. This is significant since it impacts on our interpretation of the absolute timing of 2R, with the age estimate reflecting the divergence of the two lineages that later hybridised, not the allopolyploidy event itself[196] es. Thus, if 2R is an allopolyploidy event, it occurred after 498.4-485.2 Ma (late Cambrian), potentially post-dating the acquisition of gnathostome novelties that accrued among ostracoderms prior to the divergence of crown-gnathostomes (which definitively post-date 2R) at 450.1-445.1 Ma (late Ordovician). By the same token, the macroevolutionary consequences of allopolyploidy are expected to be more immediate than autopolyploidy, resulting in chromosomal rearrangements, changes in chromatin structure, DNA methylation, gene expression and the activation of transposable elements[197–199], extensive and immediate changes that promote species and ecological diversification[200,201] as well as evolutionary novelty[202–204]. This may go some way to explain why the evolutionary consequences of the 2R WGD are so much

greater, leading to the profound diversification of gnathostome bodyplans that have dominated vertebrate communities since the early Palaeozoic.

# BIBLIOGRAPHY


1. Sambrook, J. & Russell, D. W. *Molecular Cloning: A Laboratory Manual*. vol. 1 (Cold Spring Harbor Laboratory, 2001).
2. Magoč, T. & Salzberg, S. L. FLASH: fast length adjustment of short reads to improve genome assemblies. *Bioinformatics* **27**, 2957–2963 (2011).
3. Li, R. *et al.* The sequence and de novo assembly of the giant panda genome. *Nature* **463**, 311–7 (2010).
4. Marlétaz, F. *et al.* Amphioxus functional genomics and the origins of vertebrate gene regulation. *Nature* **564**, 64–70 (2018).
5. Nakai, Y. *et al.* Chromosome elimination in three Baltic, south Pacific and north-east Pacific hagfish species. *Chromosome Res* **3**, 321–30 (1995).
6. Kuraku, S. Insights into cyclostome phylogenomics: pre-2R or post-2R. *Zoolog. Sci.* **25**, 960–8 (2008).
7. Simpson, J. T. *et al.* ABySS: A parallel assembler for short read sequence data. *Genome Res.* **19**, 1117–1123 (2009).
8. Luo, R. *et al.* SOAPdenovo2: an empirically improved memory-efficient short-read de novo assembler. *GigaScience* **1**, 18 (2012).
9. Putnam, N. H. *et al.* Chromosome-scale shotgun assembly using an in vitro method for long-range linkage. *Genome Res.* **26**, 342–350 (2016).
10. Kohno, S., Kubota, S. & Nakai, Y. Chromatin Diminution and Chromosome Elimination in Hagfishes. in *The Biology of Hagfishes* 81–100 (Springer Netherlands, 1998). doi:10.1007/978-94-011-5834-3_6.
11. Nagao, K. *et al.* Novel selectively amplified DNA sequences in the germline genome of the Japanese hagfish, Eptatretus burgeri. *Sci. Rep.* **12**, 21373 (2022).
12. Walker, B. J. *et al.* Pilon: An Integrated Tool for Comprehensive Microbial Variant Detection and Genome Assembly Improvement. *PLOS ONE* **9**, e112963 (2014).
13. Langmead, B. & Salzberg, S. L. Fast gapped-read alignment with Bowtie 2. *Nat. Methods* **9**, 357–359 (2012).
14. Cunningham, F. *et al.* Ensembl 2019. *Nucleic Acids Res.* **47**, D745–D751 (2019).
15. Belaghzal, H., Dekker, J. & Gibcus, J. H. Hi-C 2.0: An optimized Hi-C procedure for high-resolution genome-wide mapping of chromosome conformation. *Methods* **123**, 56–65 (2017).
16. Wingett, S. W. *et al.* HiCUP: pipeline for mapping and processing Hi-C data.

*F1000Research* **4**, 1310 (2015).

17. Burton, J. N. *et al.* Chromosome-scale scaffolding of de novo genome assemblies based on chromatin interactions. *Nat. Biotechnol.* **31**, 1119–1125 (2013).
18. Nakai, Y., Kubota, S. & Kohno, S. Chromatin diminution and chromosome elimination in four Japanese hagfish species. *Cytogenet. Cell Genet.* **56**, 196–198 (1991).
19. Rao, S. S. P. *et al.* A 3D Map of the Human Genome at Kilobase Resolution Reveals Principles of Chromatin Looping. *Cell* **159**, 1665–1680 (2014).
20. Kronenberg, Z. N. *et al.* High-resolution comparative analysis of great ape genomes. *Science* **360**, eaar6343 (2018).
21. Sun, J. *et al.* Population Genomic Analysis and De Novo Assembly Reveal the Origin of Weedy Rice as an Evolutionary Game. *Mol. Plant* **12**, 632–647 (2019).
22. Li, H. & Durbin, R. Fast and accurate short read alignment with Burrows-Wheeler transform. *Bioinforma. Oxf. Engl.* **25**, 1754–60 (2009).
23. Quinlan, A. R. & Hall, I. M. BEDTools: a flexible suite of utilities for comparing genomic features. *Bioinformatics* **26**, 841–842 (2010).
24. Pascual-Anaya, J. *et al.* Hagfish and lamprey Hox genes reveal conservation of temporal colinearity in vertebrates. *Nat. Ecol. Evol.* **2**, 859–866 (2018).
25. Altschul, S. F., Gish, W., Miller, W., Myers, E. W. & Lipman, D. J. Basic local alignment search tool. *J. Mol. Biol.* **215**, 403–410 (1990).
26. Kozomara, A., Birgaoanu, M. & Griffiths-Jones, S. miRBase: from microRNA sequences to function. *Nucleic Acids Res.* **47**, D155–D162 (2019).
27. Gruber, A. R., Lorenz, R., Bernhart, S. H., Neuböck, R. & Hofacker, I. L. The Vienna RNA Websuite. *Nucleic Acids Res.* **36**, W70–W74 (2008).
28. Kalvari, I. *et al.* Rfam 13.0: shifting to a genome-centric resource for non-coding RNA families. *Nucleic Acids Res.* **46**, D335–D342 (2018).
29. Nawrocki, E. P. & Eddy, S. R. Infernal 1.1: 100-fold faster RNA homology searches. *Bioinformatics* **29**, 2933–2935 (2013).
30. Ondov, B. D. *et al.* Mash: fast genome and metagenome distance estimation using MinHash. *Genome Biol.* **17**, 132 (2016).
31. Harris, R. S. Improved pairwise alignment of genomic DNA. (The Pennsylvania State University, 2007).
32. Herrero, J. *et al.* Ensembl comparative genomics resources. *Database* **2016**, bav096 (2016).
33. Kent, W. J., Baertsch, R., Hinrichs, A., Miller, W. & Haussler, D. Evolution's cauldron: duplication, deletion, and rearrangement in the mouse and human genomes. *Proc. Natl.*

*Acad. Sci. U. S. A.* **100**, 11484 (2003).

34. Pignatelli, M. *et al.* ncRNA orthologies in the vertebrate lineage. *Database* **2016**, bav127 (2016).

35. Donoghue, P. C. J., Forey, P. L. & Aldridge, R. J. Conodont affinity and chordate phylogeny. *Biol. Rev.* **75**, 191–251 (2000).

36. Donoghue, P. C. J. & Smith, M. P. The anatomy of Turinia pagei (Powrie), and the phylogenetic status of the Thelodonti. *Trans. R. Soc. Edinb.-Earth Sci.* **92**, 15–37 (2001).

37. Gess, R. W., Coates, M. I. & Rubidge, B. S. A lamprey from the Devonian period of South Africa. *Nature* **443**, 981–984 (2006).

38. Khonsari, R. H., Li, B., Vernier, P., Northcutt, R. G. & Janvier, P. Agnathan brain anatomy and craniate phylogeny. *Acta Zool.* **90**, 52–68 (2009).

39. Løvtrup, S. *The phylogeny of the Vertebrata*. (Wiley, 1977).

40. Sansom, R. S., Freedman, K., Gabbott, S. E., Aldridge, R. J. & Purnell, M. A. Taphonomy and affinity of an enigmatic Silurian vertebrate, Jamoytius kerwoodi White. *Palaeontology* **53**, 1393–1409 (2010).

41. Turner, S. *et al.* False teeth: conodont-vertebrate phylogenetic relationships revisited. *Geodiversitas* **32**, 545–594 (2010).

42. Heimberg, A. M., Cowper-Sal·lari, R., Sémon, M., Donoghue, P. C. J. & Peterson, K. J. microRNAs reveal the interrelationships of hagfish, lampreys, and gnathostomes and the nature of the ancestral vertebrate. *Proc. Natl. Acad. Sci.* **107**, 19379–19383 (2010).

43. Gabbott, S. E. *et al.* Pigmented anatomy in Carboniferous cyclostomes and the evolution of the vertebrate eye. *Proc. R. Soc. B Biol. Sci.* **283**, 20161151 (2016).

44. Miyashita, T. *et al.* Hagfish from the Cretaceous Tethys Sea and a reconciliation of the morphological–molecular conflict in early vertebrate phylogeny. *Proc. Natl. Acad. Sci.* **116**, 2146–2151 (2019).

45. Blair, J. E. & Hedges, S. B. Molecular phylogeny and divergence times of deuterostome animals. *Mol. Biol. Evol.* **22**, 2275–2284 (2005).

46. Delsuc, F., Tsagkogeorga, G., Lartillot, N. & Philippe, H. Additional molecular support for the new chordate phylogeny. *Genesis* **46**, 592–604 (2008).

47. dos Reis, M. *et al.* Uncertainty in the timing of origin of animals and the limits of precision in molecular timescales. *Curr. Biol.* **25**, 2939–2950 (2015).

48. Lartillot, N. & Philippe, H. Improvement of molecular phylogenetic inference and the phylogeny of Bilateria. *Philos. Trans. R. Soc. B Biol. Sci.* **363**, 1463–1472 (2008).

49. Near, T. J. Conflict and resolution between phylogenies inferred from molecular and

phenotypic data sets for hagfish, lampreys, and gnathostomes. *J. Exp. Zoolog. B Mol. Dev. Evol.* **312B**, 749–761 (2009).

50. Stock, D. W. & Whitt, G. S. Evidence from 18S ribosomal RNA sequences that lampreys and hagfishes form a natural group. *Science* **257**, 787–789 (1992).

51. Shimodaira, H. An Approximately Unbiased Test of Phylogenetic Tree Selection. *Syst. Biol.* **51**, 492–508 (2002).

52. Kishino, H. & Hasegawa, M. Evaluation of the maximum likelihood estimate of the evolutionary tree topologies from DNA sequence data, and the branching order in Hominoidea. *J. Mol. Evol.* **29**, 170–179 (1989).

53. Shimodaira, H. & Hasegawa, M. Multiple comparisons of log-likelihoods with applications to phylogenetic inference. *Mol. Biol. Evol.* **16**, 1114–1116 (1999).

54. Matthews, J. J. *et al.* A chronostratigraphic framework for the rise of the Ediacaran macrobiota: New constraints from Mistaken Point Ecological Reserve, Newfoundland. *GSA Bull.* (2020) doi:10.1130/b35646.1.

55. Liu, A. G., McIlroy, D., Matthews, J. J. & Brasier, M. D. A new assemblage of juvenile Ediacaran fronds from the Drook Formation, Newfoundland. *J. Geol. Soc.* **169**, 395–403 (2012).

56. Dunn, F. S. *et al.* The developmental biology of Charnia and the eumetazoan affinity of the Ediacaran rangeomorphs. *Sci. Adv.* **7**, eabe0291 (2021).

57. Yang, C. *et al.* The tempo of Ediacaran evolution. *Sci. Adv.* **7**, eabi9643 (2021).

58. Dunn, F. S. *et al.* A crown-group cnidarian from the Ediacaran of Charnwood Forest, UK. *Nat. Ecol. Evol.* (2022) doi:10.1038/s41559-022-01807-x.

59. Wilby, P. R., Carney, J. N. & Howe, M. P. A. A rich Ediacaran assemblage from eastern Avalonia: Evidence of early widespread diversity in the deep ocean. *Geology* **39**, 655–658 (2011).

60. Yin, Z. *et al.* Diverse and complex developmental mechanisms of early Ediacaran embryo-like fossils from the Weng'an Biota, southwest China. *Philos. Trans. R. Soc. B Biol. Sci.* **377**, (2022).

61. Benton, M. J. *et al.* Constraints on the timescale of animal evolutionary history. *Palaeontol. Electron.* **18**, 1–106 (2015).

62. Steiner, M., Li, G., Qian, Y., Zhu, M. & Erdtmann, B.-D. Neoproterozoic to Early Cambrian small shelly fossil assemblages and a revised biostratigraphic correlation of the Yangtze Platform (China). *Palaeogeogr. Palaeoclimatol. Palaeoecol.* **254**, 67–99 (2007).

63. Runnegar, B. Muscle scars, shell form and torsion in Cambrian and Ordovician univalved molluscs. *Lethaia* **14**, 311–322 (1981).

64. Wolfe, J. M., Daley, A. C., Legg, D. A. & Edgecombe, G. D. Fossil calibrations for the arthropod Tree of Life. *Earth-Sci. Rev.* **160**, 43–110 (2016).

65. Narbonne, G. M. & Hofmann, H. J. Ediacaran Biota of the Wernecke Mountains, Yukon, Canada. *Palaeontology* **30**, 647–676 (1987).

66. Shu, D.-G., Conway Morris, S., Zhang, Z.-F. & Han, J. The earliest history of the deuterostomes: the importance of the Chengjiang Fossil-Lagerstätte. *Proc. R. Soc. B Biol. Sci.* **277**, 165–174 (2010).

67. Yang, C., Li, X.-H., Zhu, M., Condon, D. J. & Chen, J. Geochronological constraint on the Cambrian Chengjiang biota, South China. *J. Geol. Soc.* (2018) doi:10.1144/jgs2017-103.

68. Clausen, S. & Smith, A. B. Stem structure and evolution in the earliest pelmatozoan echinoderms. *Palaeontology* **82**, 737–748 (2008).

69. Swalla, B. J. & Smith, A. B. Deciphering deuterostome phylogeny: molecular, morphological and palaeontological perspectives. *Philos. Trans. R. Soc. B Biol. Sci.* **363**, 1557–1568 (2008).

70. Dong, X. & Zhang, H. Middle Cambrian through lowermost Ordovician conodonts from Hunan, South China. *J. Paleontol.* **91**, 1–89 (2017).

71. Donoghue, P. C. J. & Keating, J. N. Early vertebrate evolution. *Palaeontology* **57**, 879–893 (2014).

72. Murdock, D. J. E. *et al.* The origin of conodonts and of vertebrate mineralized skeletons. *Nature* **502**, 546–549 (2013).

73. Zhu, M. *et al.* The oldest articulated osteichthyan reveals mosaic gnathostome characters. *Nature* **458**, 469–474 (2009).

74. Evans, S. E. & King, M. E. A new specimens of Protosaurus (Reptilia: Diapsida) from the Marl Slate (Late Permian) of Britain. *Proc. Yorks. Geol. Soc.* **49**, 229–234 (1993).

75. Joyce, W. G. Phylogenetic relationships of Mesozoic turtles. *Bull. Peabody Mus. Nat. Hist.* **48**, 3–102 (2007).

76. Joyce, W. G. & Sterli, J. Congruence, non-homology, and the phylogeny of basal turtles. *Acta Zool.* no-no (2010) doi:10.1111/j.1463-6395.2010.00491.x.

77. Cúneo, R. *et al.* High-precision U–Pb geochronology and a new chronostratigraphy for the Cañadón Asfalto Basin, Chubut, central Patagonia: Implications for terrestrial faunal and floral evolution in Jurassic. *Gondwana Res.* **24**, 1267–1275 (2013).

78. Rougier, G. W., Martinelli, A. G., Forasiepi, A. M. & Novacek, M. J. New Jurassic mammals from Patagonia, Argentina: a reappraisal of australosphenidan morphology and interrelationships. *Am. Mus. Novit.* **2007**, 1–54 (2007).

79. Alvarez-Carretero, S. *et al.* A species-level timeline of mammal evolution integrating

phylogenomic data. *Nature* **602**, 263–267 (2022).

80. Luo, Z.-X. *et al.* Evolutionary development in basal mammaliaforms as revealed by a docodontan. *Science* **347**, 760–764 (2015).

81. Luo, Z. X., Ji, Q., Wible, J. R. & Yuan, C. X. An early Cretaceous tribosphenic mammal and metatherian evolution. *Science* **302**, 1934–1940 (2003).

82. Yuan, C.-X., Ji, Q., Meng, Q.-J., Tabrum, A. R. & Luo, Z.-X. Earliest evolution of multituberculate mammals revealed by a new Jurassic fossil. *Science* **341**, 779–783 (2013).

83. Bi, S., Wang, Y., Guan, J., Sheng, X. & Meng, J. Three new Jurassic euharamiyidan species reinforce early divergence of mammals. *Nature* **514**, 579–84 (2014).

84. Meng, J., Hu, Y., Li, C. & Wang, Y. The mammal fauna in the Early Cretaceous Jehol Biota: implications for diversity and biology of Mesozoic mammals. *Geol. J.* **41**, 439–463 (2006).

85. Ogg, J. G. & Hinnov, L. A. Jurassic. in *The geologic time scale 2012* (eds. Gradstein, F. M., Ogg, J. G., Schmitz, M. & Ogg, G.) 731–791 (Elsevier, 2012).

86. Woodburne, M. O., Rich, T. H. & Springer, M. S. The evolution of tribospheny and the antiquity of mammalian clades. *Mol. Phylogenet. Evol.* **28**, 360–385 (2003).

87. Fox, R. C., Scott, C. S. & Rankin, B. D. New early carnivoran specimens from the Puercan (earliest Paleocene) of Saskatchewan, Canada. *J. Paleontol.* **84**, 1035–1039 (2010).

88. Fox, R. C. & Youzwyshyn, G. P. New primitive carnivorans (Mammalia) from the Paleocene of Western Canada, and their bearing on relationships of the order. *J. Vertebr. Paleontol.* **14**, 382–404 (2008).

89. Clemens, W. A. & Wilson, G. P. Early Torrejonian mammalian local fauna from northeastern Montana, U.S.A. *Mus. North. Ariz. Bull.* **65**, 111–158 (2009).

90. Gheerbrant, E. Paleocene emergence of elephant relatives and the rapid radiation of African ungulates. *Proc. Natl. Acad. Sci.* **106**, 10717–10721 (2009).

91. Beck, R. M. An 'ameridelphian' marsupial from the early Eocene of Australia supports a complex model of Southern Hemisphere marsupial biogeography. *Naturwissenschaften* **99**, 715–729 (2012).

92. Beck, R. M., Godthelp, H., Weisbecker, V., Archer, M. & Hand, S. J. Australia's oldest marsupial fossils and their biogeographical implications. *PLoS ONE* **3:e1858**, (2008).

93. Cannatella, D. Xenopus in space and time: fossils, node calibrations, tip-dating, and paleobiogeography. *Cytogenet Genome Res* **145**, 283–301 (2015).

94. Báez, A. M. Anurans from the Early Cretaceous Lagerstätte of Las Hoyas, Spain: new evidence on the Mesozoic diversification of the crownclade Anura. *Cretac. Res.* **41**, 90–106 (2013).

95. Henrici, A. C. A new pipoid anuran from the Late Jurassic Morrison Formation at Dinosaur National Monument, Utah. *J. Vertebr. Paleontol.* **18**, 321–332 (1998).

96. Kowallis, B. J., Christiansen, E. H., Deino, A. L., Peterson, F. & Turner, C. E. The age of the Morrison Formation. *Mod. Geol.* **22**, 235–260 (1998).

97. Piveteau, J. Paléontologie de Madagascar. XXI. Les poissons du Trias inferieur. Contribution à l'étude des actinoptérygiens. *Ann. Paléontol.* **23**, 81–180 (1935).

98. Cunningham, J. A., Vargas, K., Yin, Z., Bengtson, S. & Donoghue, P. C. J. The Weng'an Biota (Doushantuo Formation): an Ediacaran window on soft-bodied and multicellular microorganisms. *J. Geol. Soc.* **174**, 793–802 (2017).

99. Zhou, C. *et al.* A new SIMS zircon U–Pb date from the Ediacaran Doushantuo Formation: age constraint on the Weng'an biota. *Geol. Mag.* **154**, 1193–1201 (2017).

100. Guijarro-Clarke, C., Holland, P. W. H. & Paps, J. Widespread patterns of gene loss in the evolution of the animal kingdom. *Nat. Ecol. Evol.* **20**, (2020).

101. Paps, J. & Holland, P. W. H. Reconstruction of the ancestral metazoan genome reveals an increase in genomic novelty. *Nat. Commun.* **9**, 1730 (2018).

102. Boehm, T. *et al.* Evolution of Alternative Adaptive Immune Systems in Vertebrates. *Annu. Rev. Immunol.* **36**, 19–42 (2018).

103. Das, S. *et al.* Genomic donor cassette sharing during VLRA and VLRC assembly in jawless vertebrates. *Proc. Natl. Acad. Sci. U. S. A.* (2014) doi:10.1073/pnas.1415580111.

104. Lamb, T. D. Evolution of phototransduction, vertebrate photoreceptors and retina. *Prog. Retin. Eye Res.* **36**, 52–119 (2013).

105. Lamb, T. D. *et al.* Evolution of Vertebrate Phototransduction: Cascade Activation. *Mol. Biol. Evol.* **33**, 2064–2087 (2016).

106. Pisani, D., Mohun, S. M., Harris, S. R., McInerney, J. O. & Wilkinson, M. Molecular evidence for dim-light vision in the last common ancestor of the vertebrates. *Curr. Biol.* **16**, R318–R319 (2006).

107. Bailey, M. J. & Cassone, V. M. Opsin Photoisomerases in the Chick Retina and Pineal Gland: Characterization, Localization, and Circadian Regulation. *Invest. Ophthalmol. Vis. Sci.* **45**, 769–775 (2004).

108. Sun, H., Gilbert, D. J., Copeland, N. G., Jenkins, N. A. & Nathans, J. Peropsin, a novel visual pigment-like protein located in the apical microvilli of the retinal pigment epithelium. *Proc. Natl. Acad. Sci.* **94**, 9893–9898 (1997).

109. Cook, J. D. *et al.* Peropsin modulates transit of vitamin A from retina to retinal pigment epithelium. *J. Biol. Chem.* **292**, 21407–21416 (2017).

110. Emerling, C. A. & Springer, M. S. Eyes underground: Regression of visual protein

networks in subterranean mammals. *Mol. Phylogenet. Evol.* **78**, 260–270 (2014).

111. Ooka-Souda, S., Kabasawa, H. & Kinoshita, S. Circadian rhythms in locomotor activity in hagfish, Eptatretus burgeri, and the effect of reversal of light-dark cycle. *Zoolog. Sci.* **2**, 749–754 (1985).

112. Ooka-Souda, S. & Kabasawa, H. The part of the brain which is necessary for the normal swimming behaviour of the hagfish. *Zoolog. Sci.* **7**, 1022- (1990).

113. Ooka-Souda, S., Kadota, T. & Kabasawa, H. The preoptic nucleus: the probable location of the circadian pacemaker of the hagfish, Eptatretus burgeri. *Neurosci. Lett.* **164**, 33–36 (1993).

114. Ooka-Souda, S., Kadota, T., Kabasawa, H. & Takeuchi, H. A possible retinal information route to the circadian pacemaker through pretectal areas in the hagfish, Eptatretus burgeri. *Neurosci. Lett.* **192**, 201–204 (1995).

115. Collin, S. & Lamb, T. D. Photoreception in hagfishes: Insights into the evolution of vision. in *Hagfish Biology* (eds. Edwards, S. L. & Goss, G. G.) 129–148 (CRC Press, 2015). doi:10.1201/b18935-6.

116. Sun, L. *et al.* Distribution of Mammalian-Like Melanopsin in Cyclostome Retinas Exhibiting a Different Extent of Visual Functions. *PLOS ONE* **9**, e108209 (2014).

117. Ooka-Souda, S., Kabasawa, H. & Kinoshita, S. Circadian rhythms in locomotor activity of the hagfish, Eptatretus burgeri (II). The effect of brain ablation. *Zoolog. Sci.* **5**, 431–435 (1988).

118. Morita, Y., Tabata, M., Uchida, K. & Samejima, M. Pineal-dependent locomotor activity of lamprey, Lampetra japonica, measured in relation to LD cycle and circadian rhythmicity. *J. Comp. Physiol. A* **171**, 555–562 (1992).

119. Zhao, H. *et al.* Modulation of DNA Repair Systems in Blind Cavefish during Evolution in Constant Darkness. *Curr. Biol.* **28**, 3229-3243.e4 (2018).

120. Huang, S. *et al.* Decelerated genome evolution in modern vertebrates revealed by analysis of multiple lancelet genomes. *Nat. Commun.* **5**, 5896 (2014).

121. Sacerdot, C., Louis, A., Bon, C., Berthelot, C. & Roest Crollius, H. Chromosome evolution at the origin of the ancestral vertebrate genome. *Genome Biol.* **19**, 166 (2018).

122. Simakov, O. *et al.* Deeply conserved synteny resolves early events in vertebrate evolution. *Nat. Ecol. Evol.* **4**, 820–830 (2020).

123. Simakov, O. *et al.* Deeply conserved synteny and the evolution of metazoan chromosomes. *Sci. Adv.* **8**, eabi5884 (2022).

124. Nakatani, Y. *et al.* Reconstruction of proto-vertebrate, proto-cyclostome and proto-gnathostome genomes provides new insights into early vertebrate evolution. *Nat. Commun.*

**12**, 4489 (2021).

125. Löytynoja, A. & Goldman, N. Phylogeny-Aware Gap Placement Prevents Errors in Sequence Alignment and Evolutionary Analysis. *Science* **320**, 1632–1635 (2008).

126. Salzberg, S. L. Next-generation genome annotation: we still struggle to get it right. *Genome Biol* **20**, 92 (2019).

127. Kumar, S., Stecher, G. & Tamura, K. MEGA7: Molecular Evolutionary Genetics Analysis Version 7.0 for Bigger Datasets. *Mol Biol Evol* **33**, 1870–4 (2016).

128. Kent, W. J. BLAT—The BLAST-Like Alignment Tool. *Genome Res.* **12**, 656–664 (2002).

129. Kent, W. J. *et al.* The human genome browser at UCSC. *Genome Res* **12**, 996–1006 (2002).

130. Talavera, G. & Castresana, J. Improvement of phylogenies after removing divergent and ambiguously aligned blocks from protein sequence alignments. *Syst Biol* **56**, 564–77 (2007).

131. Tan, G. *et al.* Current Methods for Automated Filtering of Multiple Sequence Alignments Frequently Worsen Single-Gene Phylogenetic Inference. *Syst Biol* **64**, 778–91 (2015).

132. Kalyaanamoorthy, S., Minh, B. Q., Wong, T. K. F., von Haeseler, A. & Jermiin, L. S. ModelFinder: fast model selection for accurate phylogenetic estimates. *Nat. Methods* **14**, 587–589 (2017).

133. Chernomor, O., von Haeseler, A. & Minh, B. Q. Terrace Aware Data Structure for Phylogenomic Inference from Supermatrices. *Syst Biol* **65**, 997–1008 (2016).

134. Kozlov, A. M., Darriba, D., Flouri, T., Morel, B. & Stamatakis, A. RAxML-NG: a fast, scalable and user-friendly tool for maximum likelihood phylogenetic inference. *Bioinformatics* **35**, 4453–4455 (2019).

135. Nguyen, L. T., Schmidt, H. A., von Haeseler, A. & Minh, B. Q. IQ-TREE: a fast and effective stochastic algorithm for estimating maximum-likelihood phylogenies. *Mol. Biol. Evol.* **32**, 268–74 (2015).

136. Lemoine, F. *et al.* Renewing Felsenstein's phylogenetic bootstrap in the era of big data. *Nature* **556**, 452–456 (2018).

137. Zhang, C., Rabiee, M., Sayyari, E. & Mirarab, S. ASTRAL-III: polynomial time species tree reconstruction from partially resolved gene trees. *BMC Bioinformatics* **19**, 153 (2018).

138. Garsmeur, O. *et al.* Two Evolutionarily Distinct Classes of Paleopolyploidy. *Mol. Biol. Evol.* **31**, 448–454 (2014).

139. Schnable, J. C., Springer, N. M. & Freeling, M. Differentiation of the maize subgenomes by genome dominance and both ancient and ongoing gene loss. *Proc. Natl. Acad. Sci.* **108**, 4069–4074 (2011).

140. Session, A. M. *et al.* Genome evolution in the allotetraploid frog Xenopus laevis. *Nature* **538**, 336–343 (2016).

141. Zhou, X., Shen, X. X., Hittinger, C. T. & Rokas, A. Evaluating Fast Maximum Likelihood-Based Phylogenetic Programs Using Empirical Phylogenomic Data Sets. *Mol Biol Evol* **35**, 486–503 (2018).

142. Huerta-Cepas, J., Serra, F. & Bork, P. ETE 3: Reconstruction, Analysis, and Visualization of Phylogenomic Data. *Mol Biol Evol* **33**, 1635–8 (2016).

143. Smith, J. J. *et al.* The sea lamprey germline genome provides insights into programmed genome rearrangement and vertebrate evolution. *Nat. Genet.* **50**, 270–277 (2018).

144. Timoshevskaya, N. *et al.* An improved germline genome assembly for the sea lamprey Petromyzon marinus illuminates the evolution of germline-specific chromosomes. *Cell Rep.* **42**, 112263 (2023).

145. Escriva, H., Manzon, L., Youson, J. & Laudet, V. Analysis of Lamprey and Hagfish Genes Reveals a Complex History of Gene Duplications During Early Vertebrate Evolution. *Mol. Biol. Evol.* **19**, 1440–1450 (2002).

146. Holland, P. W. H., Garcia-Fernandez, J., Williams, N. A. & Sidow, A. Gene duplications and the origins of vertebrate development. *Development* **120**, 125–133 (1994).

147. Brunet, F. G. *et al.* Gene loss and evolutionary rates following whole-genome duplication in teleost fishes. *Mol Biol Evol* **23**, 1808–16 (2006).

148. Lien, S. *et al.* The Atlantic salmon genome provides insights into rediploidization. *Nature* **533**, 200–205 (2016).

149. Woods, I. G. *et al.* The zebrafish gene map defines ancestral vertebrate chromosomes. *Genome Res* **15**, 1307–14 (2005).

150. Zhou, Y. *et al.* Metascape provides a biologist-oriented resource for the analysis of systems-level datasets. *Nat Commun* **10**, 1523 (2019).

151. Yu, G. *et al.* GOSemSim: an R package for measuring semantic similarity among GO terms and gene products. *Bioinformatics* **26**, 976–8 (2010).

152. Qu, Y. *et al.* The evolution of ancestral and species-specific adaptations in snowfinches at the Qinghai–Tibet Plateau. *Proc. Natl. Acad. Sci.* **118**, e2012398118 (2021).

153. Mi, H. *et al.* PANTHER version 16: a revised family classification, tree-based classification tool, enhancer regions and extensive API. *Nucleic Acids Res* **49**, D394–D403 (2021).

154. Brawand, D. *et al.* The evolution of gene expression levels in mammalian organs. *Nature* **478**, 343–8 (2011).

155. Li, B. & Dewey, C. N. RSEM: accurate transcript quantification from RNA-Seq data with or without a reference genome. *BMC Bioinformatics* **12**, 323 (2011).

156. Dobin, A. *et al.* STAR: ultrafast universal RNA-seq aligner. *Bioinformatics* **29**, 15–21 (2013).

157. Yanai, I. *et al.* Genome-wide midrange transcription profiles reveal expression level relationships in human tissue specification. *Bioinformatics* **21**, 650–659 (2005).

158. Ota, K. G. & Kuratani, S. The History of Scientific Endeavors Towards Understanding Hagfish Embryology. *Zoolog. Sci.* **23**, 403–418 (2006).

159. Oisi, Y., Ota, K. G., Kuraku, S., Fujimoto, S. & Kuratani, S. Craniofacial development of hagfishes and the evolution of vertebrates. *Nature* **493**, 175–180 (2013).

160. Acemel, R. D. *et al.* A single three-dimensional chromatin compartment in amphioxus indicates a stepwise evolution of vertebrate Hox bimodal regulation. *Nat. Genet.* **48**, 336–341 (2016).

161. Buenrostro, J. D., Giresi, P. G., Zaba, L. C., Chang, H. Y. & Greenleaf, W. J. Transposition of native chromatin for fast and sensitive epigenomic profiling of open chromatin, DNA-binding proteins and nucleosome position. *Nat. Methods* **10**, 1213–8 (2013).

162. Uesaka, M., Kuratani, S., Takeda, H. & Irie, N. Recapitulation-like developmental transitions of chromatin accessibility in vertebrates. *Zool. Lett.* **5**, 33 (2019).

163. Gaspar, J. M. NGmerge: merging paired-end reads via novel empirically-derived models of sequencing errors. *BMC Bioinformatics* **19**, 536 (2018).

164. Howe, K. *et al.* The zebrafish reference genome sequence and its relationship to the human genome. *Nature* (2013) doi:10.1038/nature12111.

165. Kasahara, M. *et al.* The medaka draft genome and insights into vertebrate genome evolution. *Nature* **447**, 714–719 (2007).

166. Chinwalla, A. T. *et al.* Initial sequencing and comparative analysis of the mouse genome. *Nature* **420**, 520–562 (2002).

167. Howe, K. L. *et al.* Ensembl 2021. *Nucleic Acids Res.* **49**, D884–D891 (2021).

168. Li, H. *et al.* The Sequence Alignment/Map format and SAMtools. *Bioinformatics* **25**, 2078–2079 (2009).

169. Zhang, Y. *et al.* Model-based analysis of ChIP-Seq (MACS). *Genome Biol.* **9**, R137 (2008).

170. Li, Q., Brown, J. B., Huang, H. & Bickel, P. J. Measuring reproducibility of high-throughput experiments. *Ann. Appl. Stat.* **5**, (2011).

171. Landt, S. G. *et al.* ChIP-seq guidelines and practices of the ENCODE and modENCODE consortia. *Genome Res.* **22**, 1813–1831 (2012).

172. McLean, C. Y. *et al.* GREAT improves functional interpretation of cis-regulatory regions. *Nat. Biotechnol.* **28**, 495–501 (2010).

173. Aburomia, R., Khaner, O. & Sidow, A. Functional evolution in the ancestral lineage of vertebrates or when genomic complexity was wagging its morphological tail. *J. Struct. Funct. Genomics* **3**, 45–52 (2003).

174. Shimeld, S. M. & Holland, P. W. H. Vertebrate innovations. *Proc. Natl. Acad. Sci. U. S. A.* **97**, 4449–52 (2000).

175. Stellwag, E. J. Are genome evolution, organism complexity and species diversity linked? *Integr. Comp. Biol.* **44**, 358–365 (2004).

176. Wagner, G. P., Amemiya, C. & Ruddle, F. Hox cluster duplications and the opportunity for evolutionary novelties. *Proc. Natl. Acad. Sci. USA* **100**, 146043–14606 (2003).

177. Ohno, S. *Evolution by gene duplication*. (Springer-Verlag, 1970).

178. Clark, J. W. & Donoghue, P. C. J. Constraining the timing of whole genome duplication in plant evolutionary history. *Proc. R. Soc. B Biol. Sci.* **284**, (2017).

179. Macqueen, D. J. & Johnston, I. A. A well-constrained estimate for the timing of the salmonid whole genome duplication reveals major decoupling from species diversification. *Proc Biol Sci* **281**, 20132881 (2014).

180. Clark, J. W., Puttick, M. N. & Donoghue, P. C. J. Origin of horsetails and the role of whole-genome duplication in plant macroevolution. *Proc Biol Sci* **286**, 20191662 (2019).

181. Clark, J. W. & Donoghue, P. C. J. Constraining whole-genome duplication events in geological time. *Methods Mol. Biol.* **2545**, 139–154 (2023).

182. Donoghue, P. & Purnell, M. Genome duplication, extinction and vertebrate evolution. *Trends Ecol. Evol.* **20**, 312–319 (2005).

183. Foote, M. Morphological disparity in Ordovician-Devonian crinoids and the early saturation of morphological space. *Palaeobiology* **20**, 320–344 (1994).

184. Braasch, I. *et al.* The spotted gar genome illuminates vertebrate evolution and facilitates human-teleost comparisons. *Nat. Genet.* **48**, 427–437 (2016).

185. Clark, J. W. & Donoghue, P. C. J. Whole-Genome Duplication and Plant Macroevolution. *Trends Plant Sci.* **23**, 933–945 (2018).

186. Clarke, J. T., Lloyd, G. T. & Friedman, M. Little evidence for enhanced phenotypic evolution in early teleosts relative to their living fossil sister group. *Proc Natl Acad Sci U A* **113**, 11531–11536 (2016).

187. Emms, D. M. & Kelly, S. OrthoFinder: phylogenetic orthology inference for comparative genomics. *Genome Biol.* **20**, 238 (2019).

188. Li, Z. *et al.* Multiple large-scale gene and genome duplications during the evolution of hexapods. *Proc Natl Acad Sci U A* (2018) doi:10.1073/pnas.1710791115.

189. Nossa, C. W. *et al.* Joint assembly and genetic mapping of the Atlantic horseshoe crab genome reveals ancient whole genome duplication. *GigaScience* **3**, 9 (2014).

190. Schwager, E. E. *et al.* The house spider genome reveals an ancient whole-genome duplication during arachnid evolution. *BMC Biol.* **15**, 62 (2017).

191. Keating, J. N. & Donoghue, P. C. J. Histology and affinity of anaspids, and the early evolution of the vertebrate dermal skeleton. *Proc. R. Soc. B Biol. Sci.* **283**, 20152917 (2016).

192. Donoghue, P. C. J., Smith, M. P. & Sansom, I. J. The origin and evolution of chordates: molecular clocks and the fossil record. in *Telling the evolutionary time: molecular clocks and the fossil record* (eds. Donoghue, P. C. J. & Smith, M. P.) 190–223 (Taylor & Francis, 2003).

193. Smith, M. P., Donoghue, P. C. J. & Sansom, I. J. The spatial and temporal diversification of Early Palaeozoic vertebrates. in *Palaeobiogeography and biodiversity change: the Ordovician and Mesozoic-Cenozoic radiations. Geological Society Special Publication No. 194* (eds. Crame, J. A. & Owen, A. W.) 69–83 (The Geological Society, 2002).

194. Crow, K. D. & Wagner, G. P. What is the role of genome duplication in the evolution of complexity and diversity? *Mol. Biol. Evol.* **23**, 887–892 (2006).

195. Peterson, K. J. *et al.* MicroRNAs as Indicators into the Causes and Consequences of Whole-Genome Duplication Events. *Mol. Biol. Evol.* **39**, msab344 (2022).

196. Doyle, J. J. & Egan, A. N. Dating the origins of polyploidy events. *New Phytol.* **186**, 73–85 (2010).

197. Kon, T. *et al.* The Genetic Basis of Morphological Diversity in Domesticated Goldfish. *Curr. Biol.* **30**, 2260-2274.e6 (2020).

198. Qiu, T., Liu, Z. & Liu, B. The effects of hybridization and genome doubling in plant evolution via allopolyploidy. *Mol. Biol. Rep.* **47**, 5549–5558 (2020).

199. Soltis, P. S. & Soltis, D. E. Ancient WGD events as drivers of key innovations in angiosperms. *Curr. Opin. Plant Biol.* **30**, 159–165 (2016).

200. Barker, M. S., Arrigo, N., Baniaga, A. E., Li, Z. & Levin, D. A. On the relative abundance of autopolyploids and allopolyploids. *New Phytol.* **210**, 391–398 (2016).

201. Estep, M. C. *et al.* Allopolyploidy, diversification, and the Miocene grassland expansion. *Proc. Natl. Acad. Sci.* **111**, 15149–15154 (2014).

202. Alix, K., Gérard, P. R., Schwarzacher, T. & Heslop-Harrison, J. S. (Pat). Polyploidy and interspecific hybridization: partners for adaptation, speciation and evolution in plants. *Ann. Bot.* **120**, 183–194 (2017).

203. Steensels, J., Gallone, B. & Verstrepen, K. J. Interspecific hybridization as a driver of

fungal evolution and adaptation. *Nat. Rev. Microbiol.* **19**, 485–500 (2021).

204. Taylor, S. A. & Larson, E. L. Insights from genomes into the evolutionary importance and prevalence of hybridization in nature. *Nat. Ecol. Evol.* **3**, 170–177 (2019).

205. Mehta, T. K. *et al.* Evidence for at least six Hox clusters in the Japanese lamprey (Lethenteron japonicum). *Proc. Natl. Acad. Sci.* **110**, 16044–16049 (2013).

206. Pascual-Anaya, J. & Böhmer, C. Evolution of Cyclostome Hox Clusters. in *Hox Modules in Evolution and Development* 121–139 (CRC Press, 2023).

207. Landt, S. G. *et al.* ChIP-seq guidelines and practices of the ENCODE and modENCODE consortia. *Genome Res.* **22**, 1813–1831 (2012).

**SUPPLEMENTARY FILES (https://figshare.com/projects/Hagfish_Genome_Project/163186)**

**Supplementary File 1 -** Hi-C maps of all cases of misassembly in version 3.2 before manual correction (related to Supplementary Figure 2).

**Supplementary File 2 -** Multifasta file with all hagfish vision-related genes.

**Supplementary File 3 -** Excel file containing Datasets 1-3 with relational information about orthologs of different species.

- Dataset 1: Between chicken and spotted gar, between chicken and elephant shark, between spotted gar and elephant shark.
- Dataset 2: Between hagfish and lamprey
- Dataset 3: Between vertebrates (chicken, spotted gar, elephant shark, hagfish and sea lamprey) and invertebrates (sea cucumber and Belcher's lancelet)

**Supplementary File 4 -** Multifasta file with 1,957 genes used in phylogenetic analyses supporting 1R/2R (132 in sea cucumber, 132 in Belcher's lancelet, 24 in Florida lancelet, 363 in hagfish, 703 in sea lamprey, 94 in elephant shark, 316 in chicken, 323 in spotted gar). Gene alignments were visually inspected and manually curated if necessary as described in Supplementary Information. Related to Figure 4a.

**Supplementary File 5 -** All 194 gene trees describing the phylogenetic position of elephant shark *C. milii* genes in chromosomal trees. Related to Figure 4a.

**Supplementary File 6 -** All 190 gene trees describing the phylogenetic position of inshore hagfish *E. burgeri* genes in chromosomal trees. Related to Figure 4a.

**Supplementary File 7 -** All 174 gene trees describing the phylogenetic position of the sea lamprey *P. marinus* genes in chromosomal trees. Related to Figure 4a.

**Supplementary File 8 -** All 116 gene trees describing the phylogenetic position of both inshore hagfish and sea lamprey genes in chromosomal trees. Related to Figure 4a.

**Supplementary File 9 -** Overlapping ratios between hagfish chromosomes (left panels) and sea lamprey scaffolds (right panels) deriving from the same AC. Related to Extended Data Figure 7a,b. Mutually (best-to-best) paralogous chromosomes are listed at the bottom of each panel.

**Supplementary File 10 -** Clustering analysis of duplicate retention profiles of orthologous chromosomes in chicken and spotted gar. Related to Extended Data Figure 7c.

**Supplementary File 11 -** Clustering analysis of duplicate retention profiles of putative orthologous chromosomes of the inshore hagfish and sea lamprey. Related to Figure 4g.

**Supplementary File 12 -** R script used for transcriptomics data normalization. Related to Figures 5f,g, and Extended Data Figure 8g-j.

**Supplementary File 13 -** Compressed folder containing the alignment used in the phylogenomics tree and the resulting tree.

**Supplementary File 14 -** Compressed folder containing the alignments, control files and tree used for dating species divergence (see Methods).

**Supplementary File 15 -** Orthogroups obtained by OrthoFinder.

**Supplementary File 16 -** Multiple sequence alignment and MrBayes options and parameter values of Agap genes, in Nexus format. Related to Extended Data Figure 4d.

**Supplementary File 17 -** Multiple sequence alignment and MrBayes options and parameter values of Gbx genes, in Nexus format. Related to Extended Data Figure 4a.

**Supplementary File 18 -** Multiple sequence alignment and MrBayes options and parameter values of Cbx genes, in Nexus format. Related to Extended Data Figure 4b.

**Supplementary File 19 -** Multiple sequence alignment and MrBayes options and parameter values of Hnrnpa genes, in Nexus format. Related to Extended Data Figure 4c.

**Supplementary File 20 -** Compressed folder containing alignments, MCMCtree control files and calibrations used for dating genome duplications in vertebrates.

**Supplementary File 21 -** Character matrix and descriptions (Vertebrate_disparity_matrix.nex) and tree with estimated ancestral character states (Disparity.tre) used for the phenotypic disparity analyses. Related to Figure 5g,h.

## SUPPLEMENTARY TABLES

**Supplementary Table 1 -** Sources of genomic and annotation data used.

**Supplementary Table 2 -** Sequencing libraries and basic statistics of clean sequencing data, after filtering, and processing steps they were used for.

**Supplementary Table 3 -** Statistics of reads before and after error correction of data from short-insert libraries.

**Supplementary Table 4 -** Stats of data used for genome size estimation based on 17-mer distribution.

**Supplementary Table 5 -** Dovetail Genomics's HiRise performance and library stats.

**Supplementary Table 6 -** Basic statistics of different genome assembly versions.

**Supplementary Table 7 -** Misassembled scaffolds in the hagfish Dovetail assembly (v3.2). 280 misassembled scaffolds involving 364 misjoining boundaries are listed here.

**Supplementary Table 8 -** After correction, hagfish scaffolds were regrouped as 19 clusters based on HiC contact information. From top to bottom, the rank of scaffolds denotes their relative orders in one cluster (pseudo-chromosome). Each scaffold is either in sense (orientation = 0) or antisense (orientation = 1) orientation in one cluster.

**Supplementary Table 9 -** All hagfish clusters have similar read coverage from DNA-seq reads.

**Supplementary Table 10 -** 41 hagfish genes were found at misassembly boundaries and removed from further analyses.

**Supplementary Table 11 -** Transcript structures identified from short read RNA-seq data. The table shows the number of unique transcript structures identified in each tissue prior to downstream filtering and QC.

**Supplementary Table 12 -**Overall GC-content percentages at genomic and coding sequence (CDS) levels of selected chordates.

**Supplementary Table 13 -** LastZ parameters used to align hagfish to lamprey, zebrafish, and human.

**Supplementary Table 14 -** Comparison of the coverage of the hagfish and lamprey genesets (coding and non-coding) by the gene-trees analysis. Ensembl v93 annotations were used.

**Supplementary Table 15 -** Number of orthologs, sequence identity, and number of high-confidence calls for hagfish orthologous relationships against lamprey, zebrafish, and human.

**Supplementary Table 16 -** Number of orthologs, sequence identity, and number of high-confidence calls for lamprey orthologous relationships against zebrafish and human.

**Supplementary Table 17 -** Number of genes that are structurally different from their orthologues in other species.

**Supplementary Table 18 -** A size summary of coding exons from 11 species.

**Supplementary Table 19 -** A size summary of introns from 11 species.

**Supplementary Table 20 -** A size summary of coding transcript from 11 species.

**Supplementary Table 21 -** A size summary of genes from 11 species.

**Supplementary Table 22 -** A summary of intergenic regions from 11 species.

**Supplementary Table 23 -** GO enrichment analysis of gene families present in the reconstructed ancestral vertebrate genome.

**Supplementary Table 24 -** Number of Homology groups (HG) gained at different notes (Novel) and their Panther GO enrichment analysis.

**Supplementary Table 25 -** Panther GO enrichment analysis of vertebrate Novel Core HGs.

**Supplementary Table 26 -** Panther GO enrichment analysis of gnathostome Novel Core HGs.

**Supplementary Table 27 -** Immune-related genes in the current version of hagfish genome.

**Supplementary Table 28 -** List of circadian clock and clock-controlled genes found in the genome of Eptatretus burgeri and their Ensembl IDs.

**Supplementary Table 29 -** Annotation information of Hox genes and their linked non-Hox genes in the genome of *E. burgeri*.

**Supplementary Table 30 -** Nomenclature update of Hox genes from Pascual-Anaya et al., 2018 (REF. 19) and this study after orthology assignment to lamprey Hox clusters.

**Supplementary Table 31 -** Numbers of best-to-best genes between back-mutated gar chromosomes and back-mutated chicken chromosomes.

**Supplementary Table 32 -** Homology between sea cucumber chromosomes, ancestral chromosomes (ACs), chicken chromosomes and gar chromosomes.

**Supplementary Table 33 -** Numbers of orthologs (reciprocal best blast hits) between Belcher's lancelet scaffolds and sea cucumber linkage groups.

**Supplementary Table 34 -** 17 ACs reconstructed on the basis of amphioxus genes.

**Supplementary Table 35 -** Numbers of best-to-best genes between linkage groups of the sea cucumber genome and choromosomes of the Florida lancelet.

**Supplementary Table 36 -** Numbers of best-to-best genes between ACs and Florida lancelet chromosomes.

**Supplementary Table 37 -** The correspondence between 17 ACs, 17 tetrads, 17 CLGs and 18 PVCs.

**Supplementary Table 38 -** Numbers of best-to-best genes between ACs and vertebrate ancestral chromosomes from different studies.

**Supplementary Table 39 -** Homologous gene sets underlying chromosomal phylogenetic analyses of chicken and gar.

**Supplementary Table 41 -** Comparison of post-1R chromosomal fusion events identified in this study with those identified in three previous studies.

**Supplementary Table 40 -** Frequency distribution of "pairwise post-1R fusion" based on random sampling.

**Supplementary Table 42 -** Homologous gene sets used to infer the position of cyclostome genes in single gene phylogenies.

**Supplementary Table 43 -** The overlapping ratio of two parts of a chromosome in chicken, spotted gar, hagfish, or sea lamprey genome after artificial splitting.

**Supplementary Table 44 -** Overlapping ratio of orthologous chromosome pairs of chicken and gar.

**Supplementary Table 45 -** Overlapping ratio of orthologous chromosome pairs in hagfish and lamprey.

**Supplementary Table 46 -** The overlapping ratio of paralogous chromosomes in chicken or spotted gar genome that correspond to one same AC.

**Supplementary Table 47 -** The overlapping ratio of putative paralogous chromosomes in hagfish or sea lamprey genome that correspond to one same AC.

**Supplementary Table 48 -** List of 2,137 ohnologs in the hagfish genome used in the regulatory evolution analysis.

**Supplementary Table 49 -** List of the 3,594 ohnologs in the chicken genome used in the regulatory evolution analysis.

**Supplementary Table 50 -** Merging Sea lamprey scaffolds.

**Supplementary Table 51 -** Sources of ATAC-seq data amphioxus, hagfish, zebrafish, medaka, chicken and mouse used for regulatory genome profilings.

**Supplementary Table 52 -** Number of ACRs in promoters.

**Supplementary Table 53 -** Statistics for Figure 5a.

**Supplementary Table 54 -** Statistics for Figure 5b (ACRs in promoters/proximal regions).

**Supplementary Table 55 -** Statistics for Figure 5b (ACRs in distal regions).

**Supplementary Table 56 -** Statistics for Figure 5c.

**Supplementary Table 57 -** Statistics for Figure 5d.

**Supplementary Table 58 -** Statistics for Extended Data Figure 8d.

**Supplementary Table 59 -** Statistics for Extended Data Figure 8e.

**Supplementary Table 60 -** Statistics information for Extended Data Figure 8g.

**Supplementary Table 61** - Statistics for Extended Data Figure 8h.

**Supplementary Table 62 -** Statistics for Extended Data Figure 8i.

## SUPPLEMENTARY FIGURES

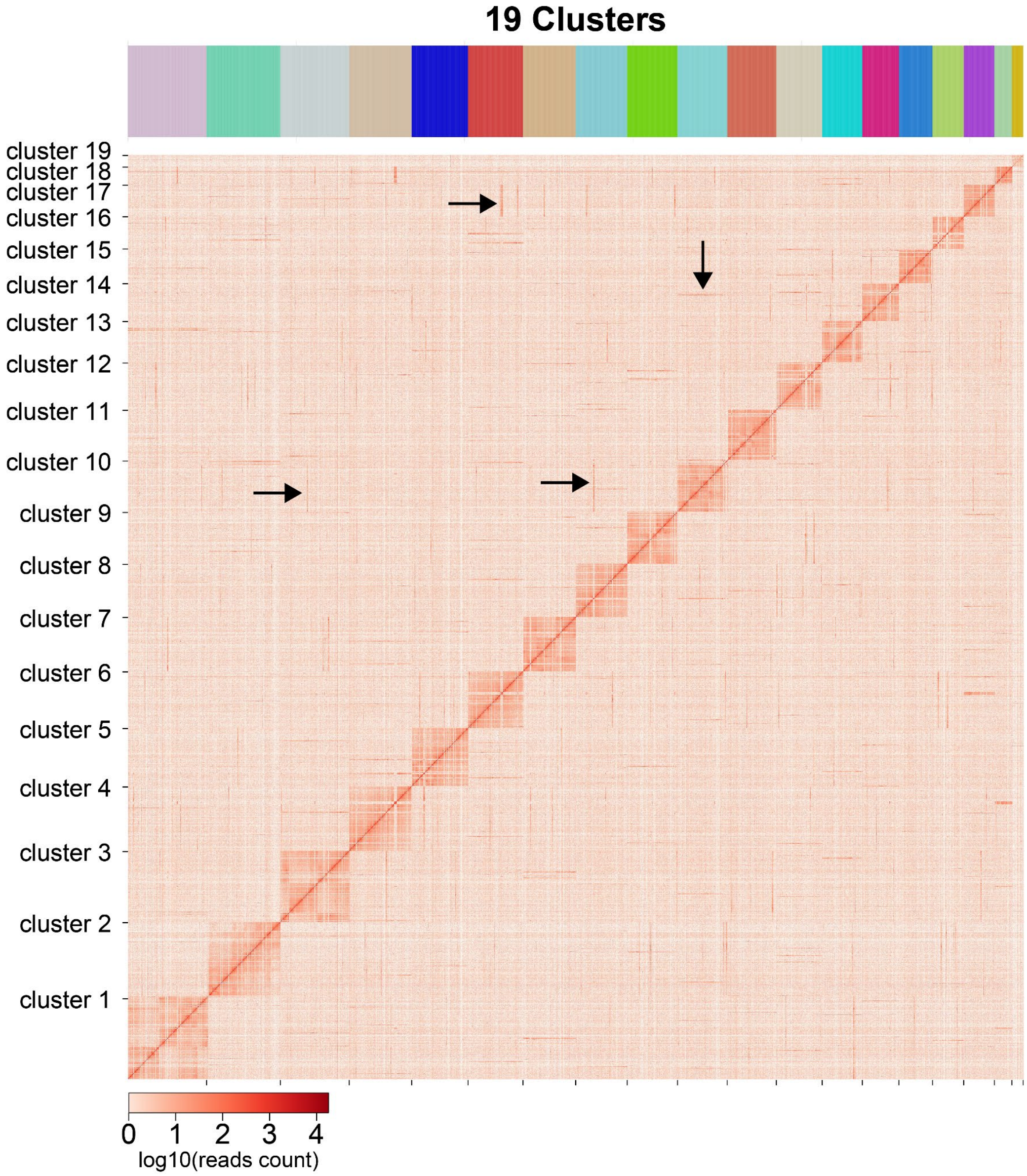


**Supplementary Figure 1. Preliminary 19-cluster Hi-C heatmap of the chromosome-scale genome assembly of *E. burgeri*.** Numerous off-diagonal long stretches of Hi-C contacts are noticeable, and a few examples are marked with black arrows. They may represent misassembled scaffolds.

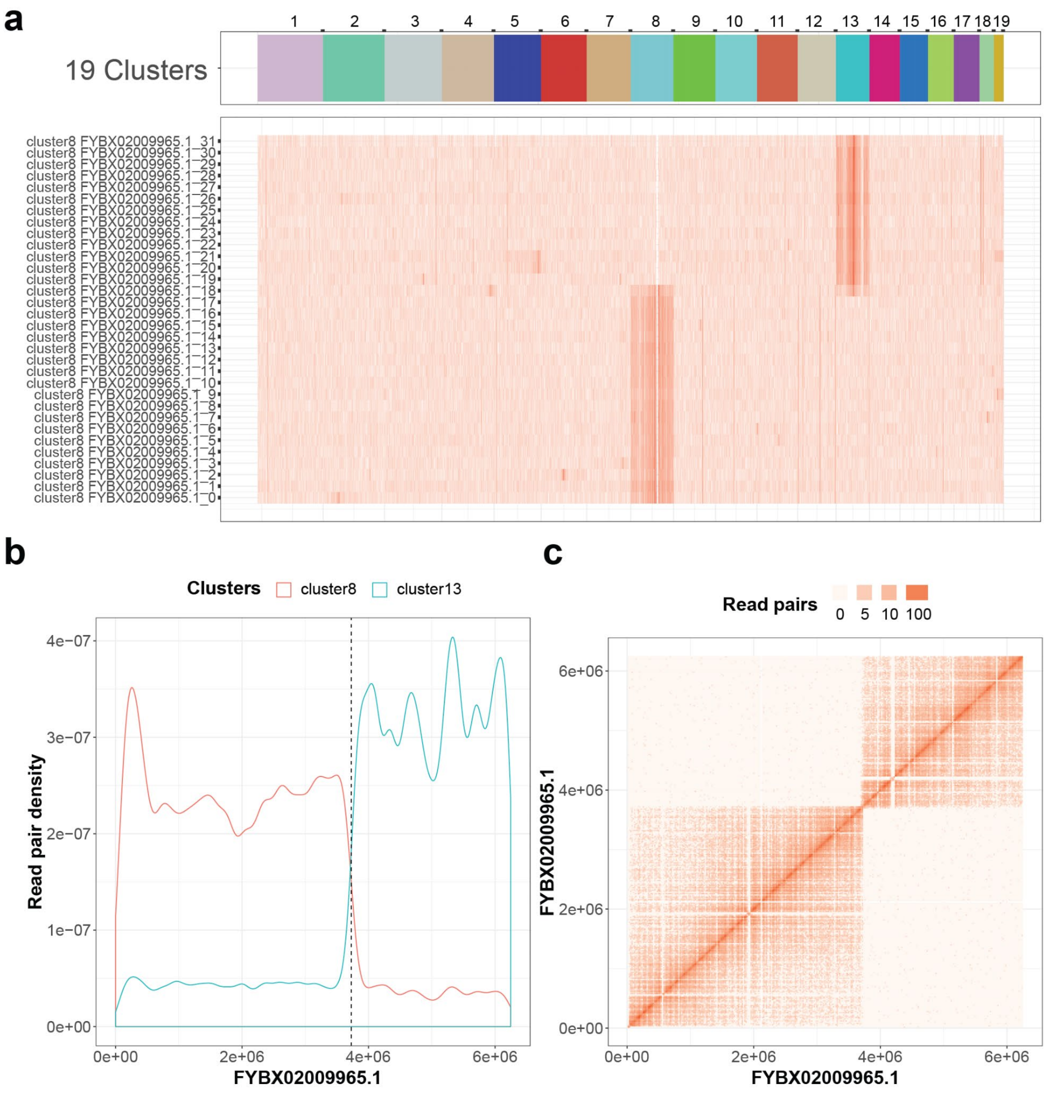


**Supplementary Figure 2. Example of a misassembly in the genome assembly version 3.2 detected with three types of Hi-C contact signals. a,** A scaffold named FYBX02009965.1 (harboring 32 200-kb segments, 0~31) is grouped into cluster8 but has abundant Hi-C contacts with cluster13 in its last thirteen segments, 19~31. **b,** The distribution of Hi-C contacts in FYBX02009965.1 relative to cluster8 or cluster13 could be divided into two partitions where the first 3.8 Mb (mega base) mainly interacts with cluster8 and the remaining 2.6 Mb mainly interacts with cluster 13. A dashed line denotes the boundary. **c,** Within-scaffold Hi-C contacts of

FYBX02009965.1 clearly shows two separate blocks. The consistency across all three panels in terms of partition boundary suggests that FYBX02009965.1 represents a misassembly of cluster8 (3.8 Mb) and cluster13 (2.6 Mb) derived segments.

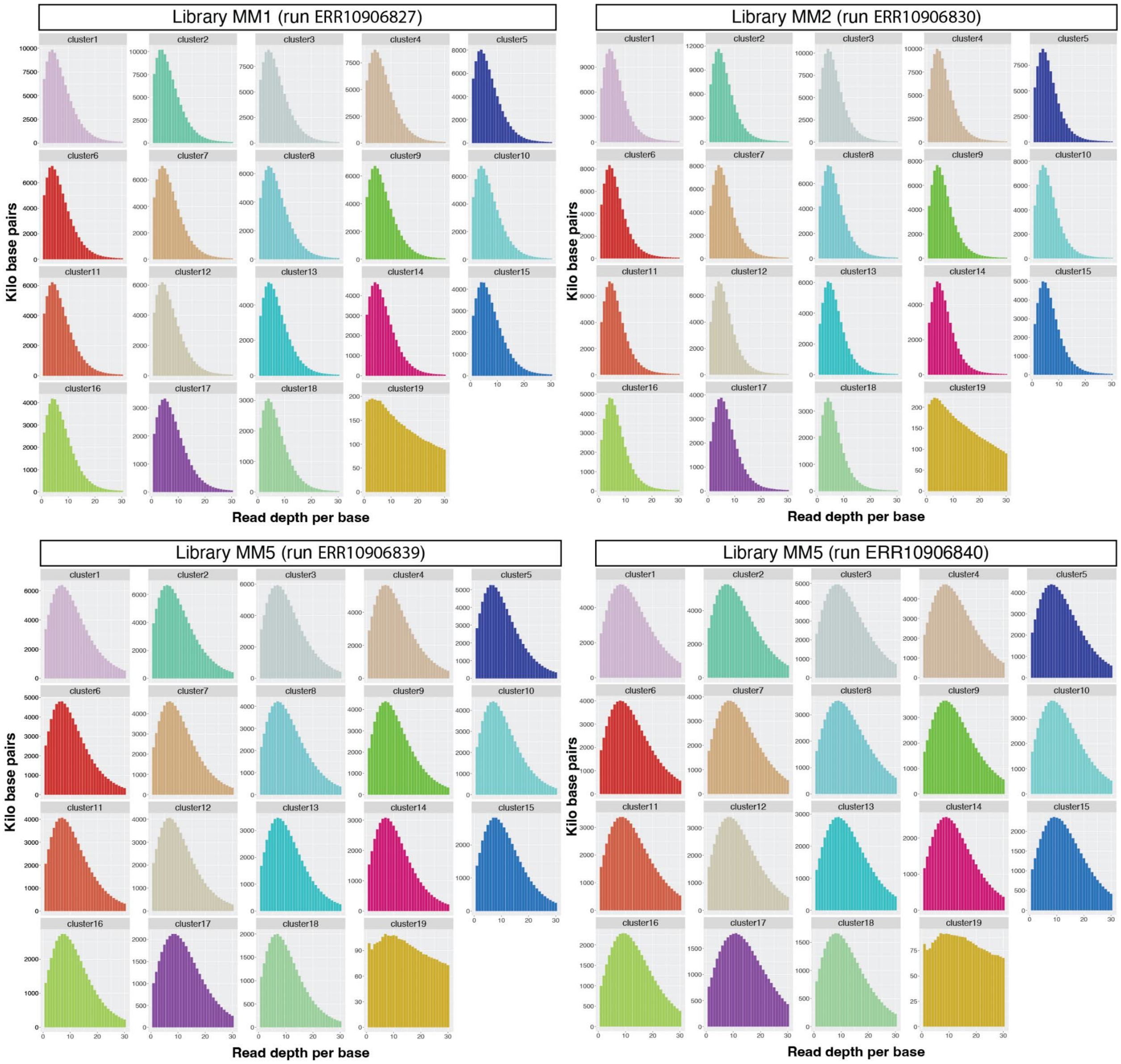


**Supplementary Figure 3. DNA sequencing read depth distribution across all 19 contact clusters of the E. burgeri genome Hi-C assembly.** Read depth per kilobases calculated using 4 randomly chosen Illumina lanes are plotted. For simplicity, read depths higher than 30 are not shown. Peak values per each run and cluster can be found in Supplementary Table 8.

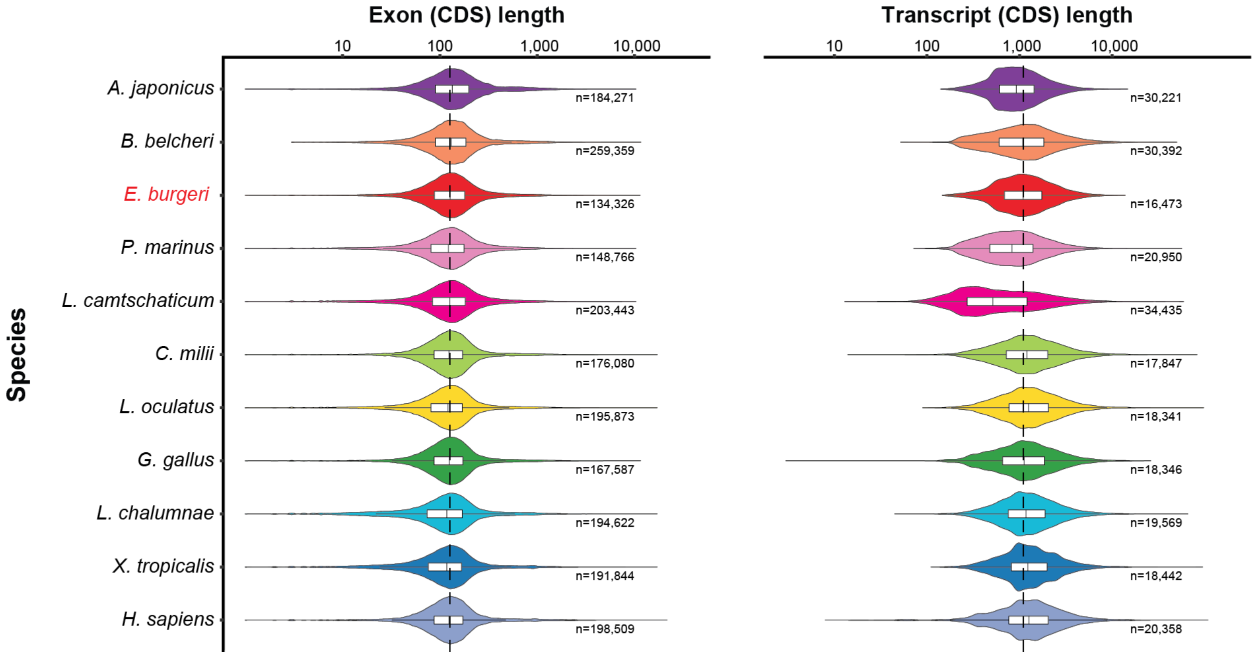


**Supplementary Figure 4. Exon and transcript lengths of vertebrate genes.** Violin plots of size distribution of exon and transcript lengths over a logarithmic scale of all annotated genes of the hagfish (*E. burgeri*), two lamprey species (sea lamprey, *P. marinus*; Arctic lamprey, *L. camtschaticum*), six gnathostome vertebrates (human, *H. sapiens*; frog, *X. tropicalis*; coelacanth, *L. chalumnae*; chicken, *G. gallus*; spotted gar, *L. oculatus*; and the elephant shark, *C. milii*) and two invertebrate deuterostomes (sea cucumber, *A. japonicus*; amphioxus, *B. belcheri*). For each genomic feature and each species, a white boxes indicates the IQR (interquartile range) length with first and third quartiles and the median indicated (centre line). Whiskers extend to the last point no further than 1.5 times the interquartile range from the first and third quartiles. Dashed vertical line indicates median size of *E. burgeri* features.

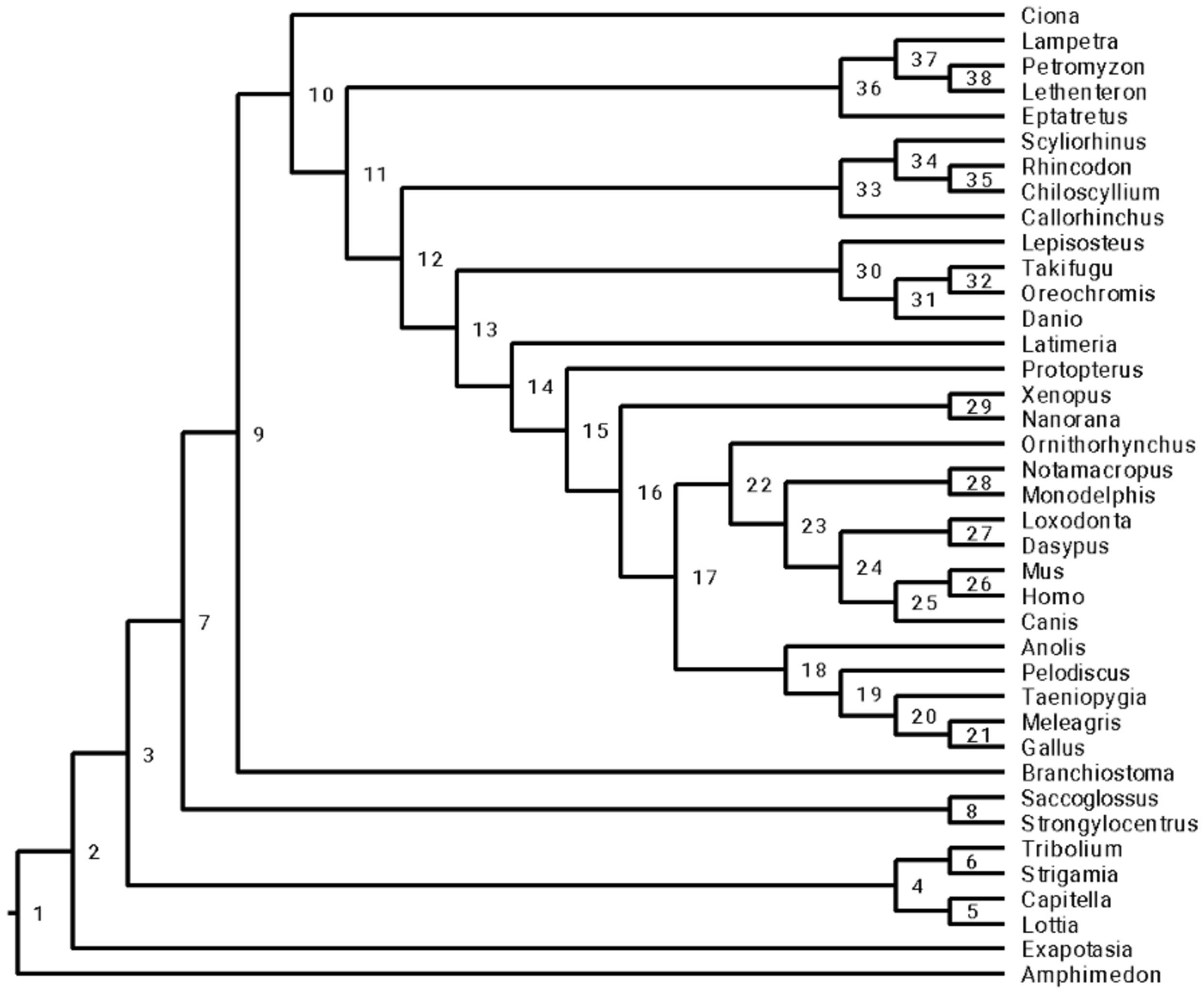


**Supplementary Figure 5. Node calibrations.** Node labels are used to indicate the node being calibrated (see section 2.2).

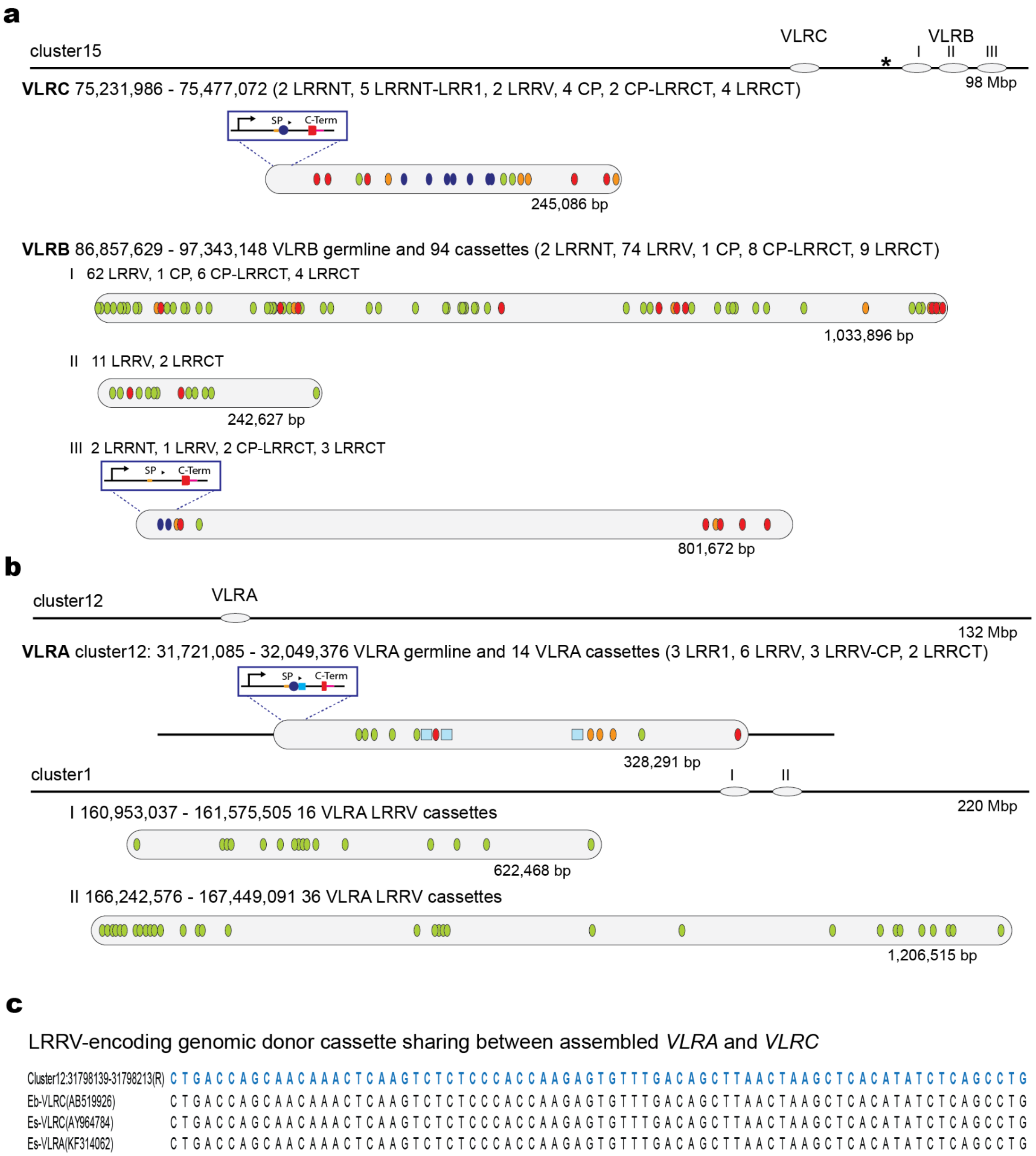


**Supplementary Figure 6. *VLR* loci organisation in the hagfish. a**, The genomic organisation of *E. burgeri VLRB* and *VLRC*. The germline *VLR* genes are located near clusters of donor cassettes. A map of assembly cluster15 is shown at the top. *VLRB* donor cassettes are encoded in three subclusters designated I, II, III and shown as light blue oval. The *VLRC* and *VLRB* germline genes

are located approximately 21 Mbp apart with 12.5 Mbp separating their associated cassette clusters. Details of the cassette organisation within clusters are shown in the larger grey ovals. Cluster sizes are given under the grey oval diagrams. Cassette icons are spaced proportionally to positions in the assembly sequence but are not drawn to scale. *VLR* germline gene structures are shown in boxes above the detailed cassette cluster representations. Cassette-types are illustrated with colored icons: dark blue ovals, *LRRNT* or *LRRNT-LRR1*; green ovals, *LRRV*; orange ovals, connecting peptide (*CP*); red ovals, *LRRCT*. For germline gene diagrams: *SP*, signal peptide; *C-*Term, invariant C-terminal region; dark blue circle, germline LRRNT; light blue square, LRR1. Four isolated *VLRB* donor cassettes located upstream of *VLRB* subcluster I are indicated by an asterisk at the top. **b**, *VLRA* is encoded on assembly cluster12 with two subclusters of *VLRA*-like *LRRV* cassettes located on assembly cluster1. Colors and icons are as in (A) with the addition of light blue squares representing individual *VLRA LRR1* cassettes. **c**, Examples of genomic donor cassette sharing between assembled *VLRA* and *VLRC* in hagfish. The genomic donor cassette sequence from the *E. burgeri* genome is shown in blue. The position of this cassette in the genome and the accession number of the mature *VLRA* and *VLRC* sequences are indicated. NB: Since only mature *VLRC* sequence (previously identified as *VLRA*) from *E. burgeri* is available in NCBI, we included *VLRA* (the third VLR) and *VLRC* from *Eptatretus stoutii*.

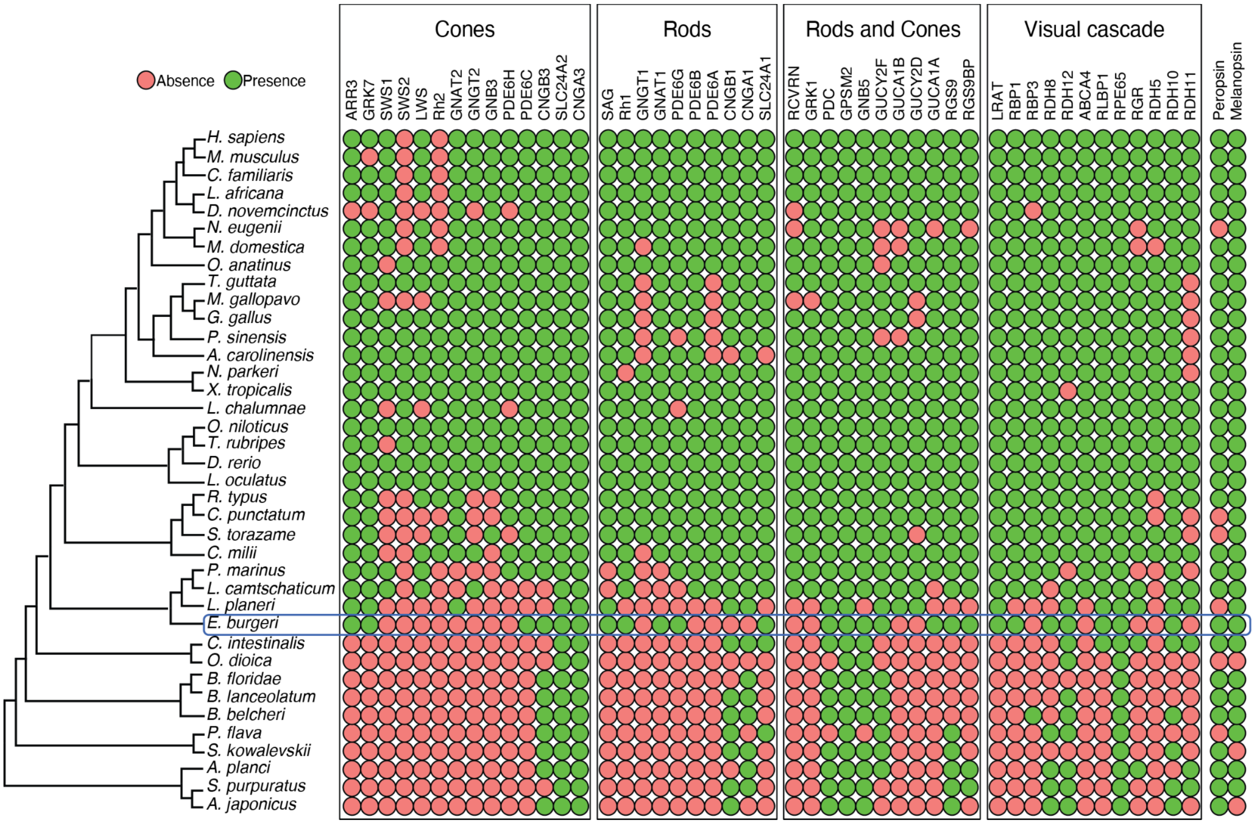


**Supplementary Figure 7. Evolution of visual genes deuterostomes.** Phylogeny of deuterostomes (left) with the presence (green) and absence (red) of vision-associated genes. The hagfish genome possesses three opsin genes: Melanopsin (Opn4), Peropsin (RRH) and Rhodopsin 1 (RH1 or RHO), but have secondarily lost most cone-specific genes, as well as several rod-specific genes. Species and the taxa they belong to can be found in Supplementary Table 1. A fasta file with *E. burgeri* gene sequences is provided in Supplementary File 2.

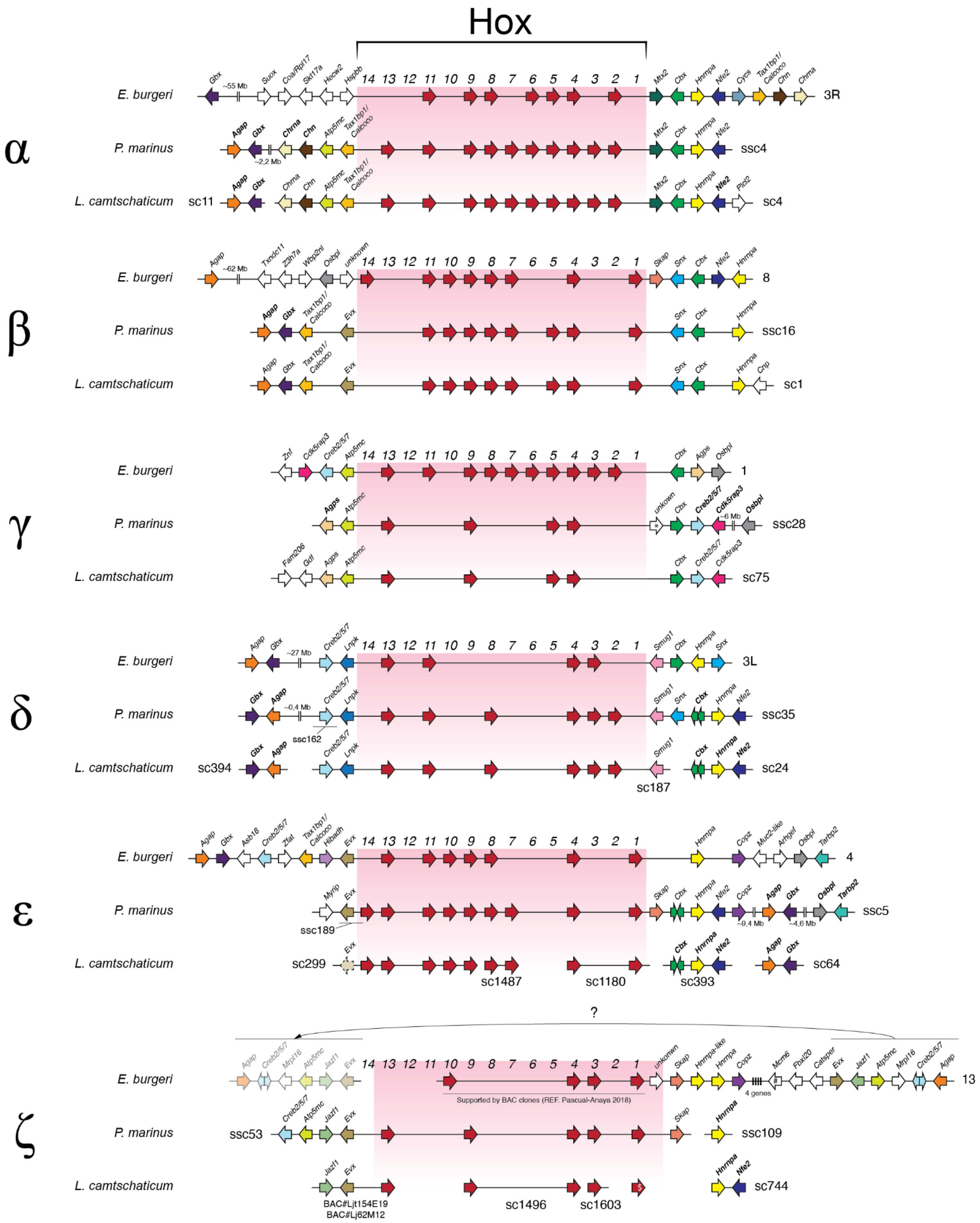

Hox
α
E. burgeri
Gbx ~55 Mb Suox Coa/Rpl17 Slc17a Hecw2 Hspbb
14 13 12 11 10 9 8 7 6 5 4 3 2 1
Mtx2 Cbx Hnrnpa Nfe2 Cyrs Tax1bp1/Calcoco Chn Chrna 3R
P. marinus
Agap Gbx ~2,2 Mb Chrna Chn Atp5mc Tax1bp1/Calcoco
Mtx2 Cbx Hnrnpa Nfe2 ssc4
L. camtschaticum
sc11 Agap Gbx Chrna Chn Atp5mc Tax1bp1/Calcoco
Mtx2 Cbx Hnrnpa Nfe2 Picl2 sc4
β
E. burgeri
Agap ~62 Mb Txndc11 Zc3h7a Wbp2nl Osbpl unknown
14 13 12 11 10 9 8 7 6 5 4 3 2 1
Skap Snx Cbx Nfe2 Hnrnpa 8
P. marinus
Agap Gbx Tax1bp1/Calcoco Evx
Snx Cbx Hnrnpa ssc16
L. camtschaticum
Agap Gbx Tax1bp1/Calcoco Evx
Snx Cbx Hnrnpa Cnp sc1
γ
E. burgeri
Znf Cdk5rap3 Creb2/5/7 Atp5mc
14 13 12 11 10 9 8 7 6 5 4 3 2 1
Cbx Agps Osbpl 1
P. marinus
Agps Atp5mc
unkown Cbx Creb2/5/7 Cdk5rap3 ~6 Mb Osbpl ssc28
L. camtschaticum
Fam206 Gdf Agps Atp5mc
Cbx Creb2/5/7 Cdk5rap3 sc75
δ
E. burgeri
Agap Gbx ~27 Mb Creb2/5/7 Lnpk
14 13 12 11 10 9 8 7 6 5 4 3 2 1
Smug1 Cbx Hnrnpa Snx 3L
P. marinus
Gbx Agap ~0,4 Mb Creb2/5/7 Lnpk ssc162
Smug1 Snx Cbx Hnrnpa Nfe2 ssc35
L. camtschaticum
sc394 Gbx Agap Creb2/5/7 Lnpk
Smug1 sc187 Cbx Hnrnpa Nfe2 sc24
ε
E. burgeri
Agap Gbx Asb18 Creb2/5/7 Zfat Tax1bp1/Calcoco Hibadh Evx
14 13 12 11 10 9 8 7 6 5 4 3 2 1
Hnrnpa Copz Muc2-like Arhgef Osbpl Tarbp2 4
P. marinus
Myrip Evx ssc189
Skap Cbx Hnrnpa Nfe2 Copz ~9,4 Mb Agap Gbx ~4,6 Mb Osbpl Tarbp2 ssc5
L. camtschaticum
sc299 Evx sc1487 sc1180
Cbx Hnrnpa Nfe2 sc393 Agap Gbx sc64
?
ζ
E. burgeri
Agap Creb2/5/7 Mrpl16 Atp5mc Jazf1 Evx
14 13 12 11 10 9 8 7 6 5 4 3 2 1
Supported by BAC clones (REF. Pascual-Anaya 2018)
unknown Skap Hnrnpa-like Hnrnpa Copz Mcm6 4 genes Fbxl20 Catsper Evx Jazf1 Atp5mc Mrpl16 Creb2/5/7 Agap 13
P. marinus
ssc53 Creb2/5/7 Atp5mc Jazf1 Evx
Skap Hnrnpa ssc109
L. camtschaticum
Jazf1 Evx BAC#Ljt154E19 BAC#Lj62M12
sc1496 sc1603
Hnrnpa Nfe2 sc744

**Supplementary Figure 8. Orthology relationships of cyclostome Hox clusters.** Orthologous *Hox* clusters of the inshore hagfish and two lamprey species (sea lamprey, *P. marinus*[143], and Arctic lamprey, *L. camstchaticum*[205]) are shown by cluster orthology. Hagfish *Hox* clusters have been assigned to each orthology group based on (i) microsynteny conservation of both Hox gene composition of each cluster and syntenic non-Hox genes, (ii) phylogenetic analyses of candidate genes (*Gbx*, *Agap*, *Hnrnpa* and *Cbx*; Extended Data Figure 4a-d), and (iii) clustering analysis of retention profiles (Extended Data Figure 4e). (i) and (ii) are sufficient to assign orthology to clusters α, γ, δ, and ζ. Assignment to Hox clusters β and ε is resolved after (iii). All genes are represented by color coded arrows according to their homology, with Hox genes in red, and whose direction indicates its sense of transcription. Separate scaffolds harbouring *Hnrnpa*, *Agap*, *Gbx* or *Cbx* genes are assigned to a particular clusters based on phylogenetic analyses. The block encompassing genes from Evx-ζ to Agap-ζ downstream to its syntenic Hox cluster might be the result of missassembly. Syntenic non-Hox genes with bold fonts are found in this study. The assembly scaffold or chromosome where they are located in their respective assemblies are indicated to the right of each cluster (ssc, superscaffold of the sea lamprey 2018 assembly; sc, scaffold of the 2013 Arctic lamprey assembly). #, Mcmc6 gene is also present in amphioxus, implying its possible ancestral link to a Hox cluster in chordates; *, PMZ_0048273 in REF. 143; $, Described in REF. 24.

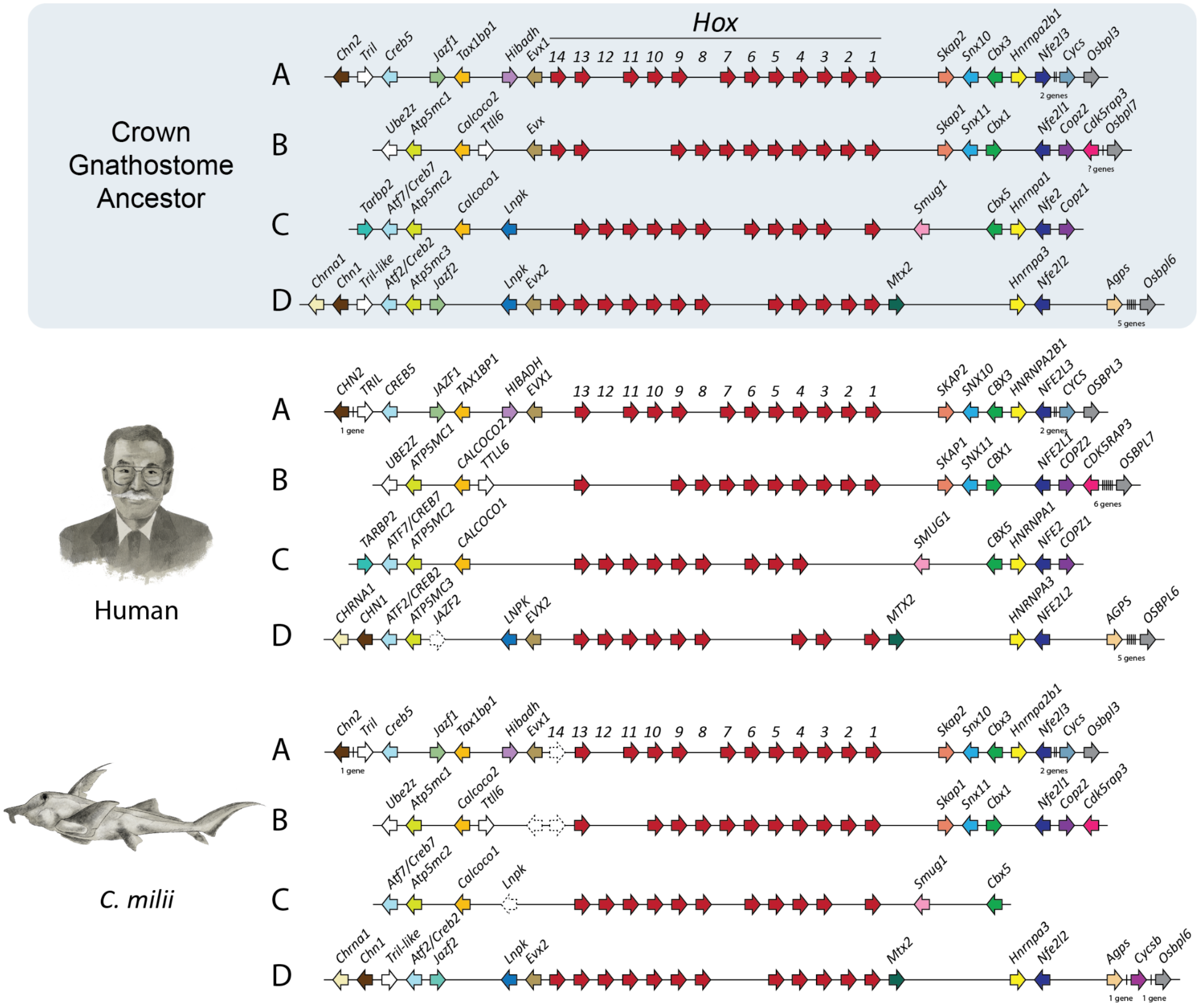


**Supplementary Figure 9. Gnathostome Hox clusters.** Schematic representations of Hox clusters and syntenic genes of the elephant shark (*C. milii*; bottom), human (middle), and a reconstruction of the complement of the last common ancestor of gnathostomes (top). Genes are represented by coloured-coded arrows, whose direction marks the sense of transcription: Hox genes in red, non-Hox genes coloured by homology. Information about syntenic genes was based on Acemel et al.[160] and manual inspection at Ensembl genome browser. Human and elephant shark illustrations kindly provided by Tamara de Dios Fernández, human illustration reproduced with permission from REF. 206.

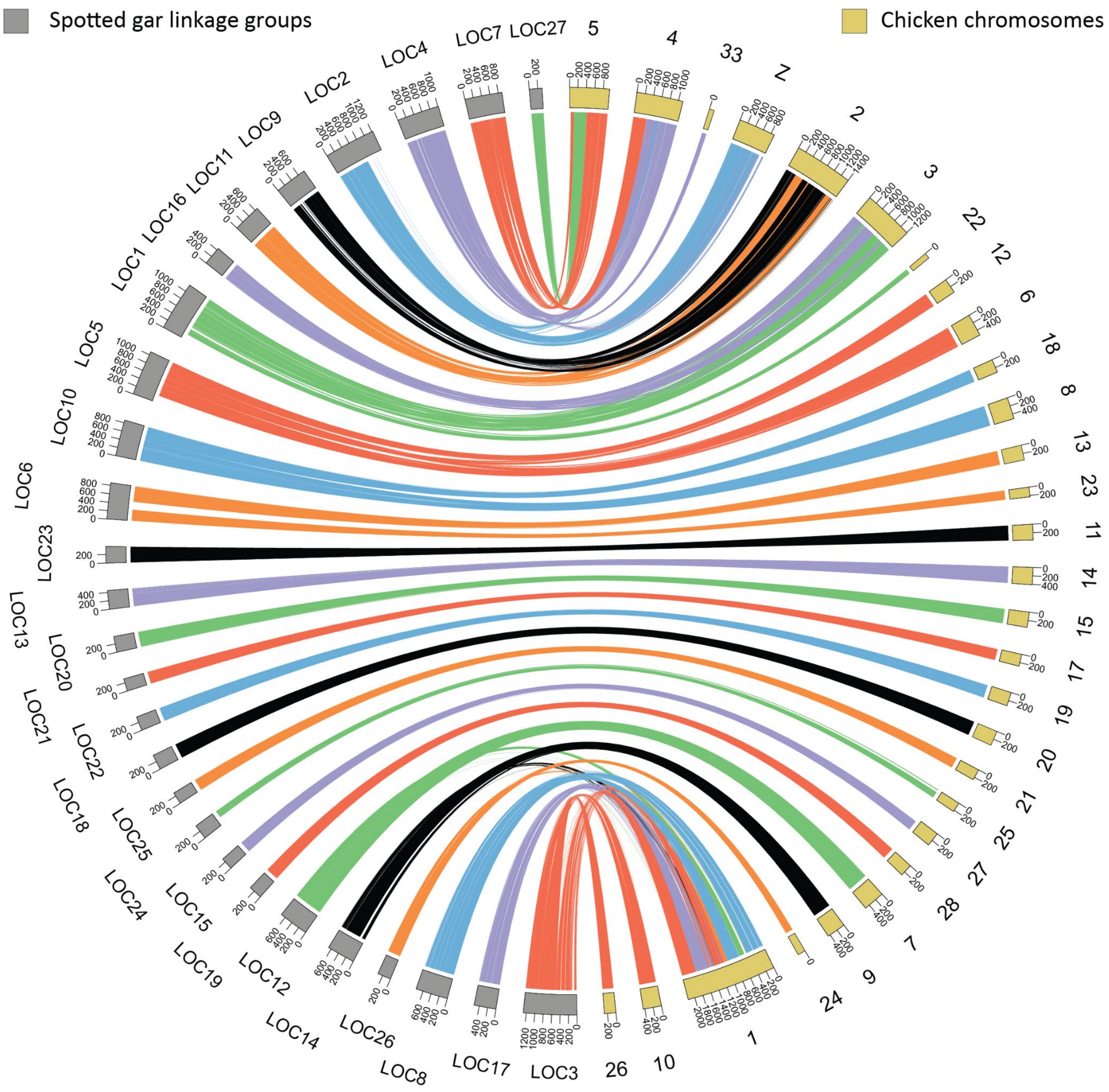


**Supplementary Figure 10. Chromosomal correspondence between chicken and gar.** A circos plot is used to show macrosynteny conservation. Each chromosome is scaled to the number of genes it contains. Chromosomes are arranged in a way to better visualise the correspondence between species. Each line represents one pair of homologous genes between chromosomes, which is colour-coded according to different spotted gar chromosomes and from a set of six colors (red/purple/blue/black/orage/green).

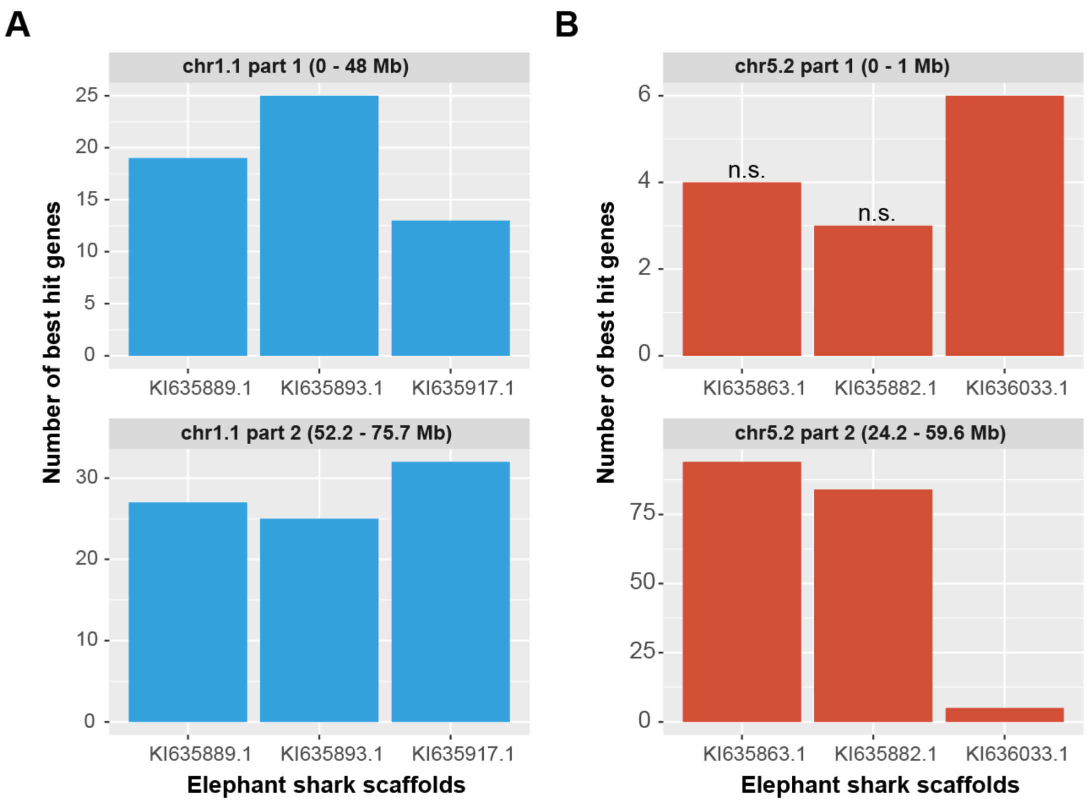


**Supplementary Figure 11. Two chicken chromosomal sections, chr1.1 or chr5.2, are homologous to the same set of shark scaffolds.** All correspondence relationships of chr1.1 (**a**) and chr5.2 (**b**) are statistically significant except that the homology of chr5.2 part 1 with elephant shark KI635863.1 or KI35882.1 is not (marked by 'n.s.'). We used 'homologous gene pair number > 5 and FDR q value < 0.05' as the cutoff to define homologous relationships. Since this segment does not harbour many genes (79 for chr5.2 part 1), only 4 and 3 homologous genes were identified in KI635863.1 and KI35882.1, respectively. We thus considered them as non-significant homologous relationships.

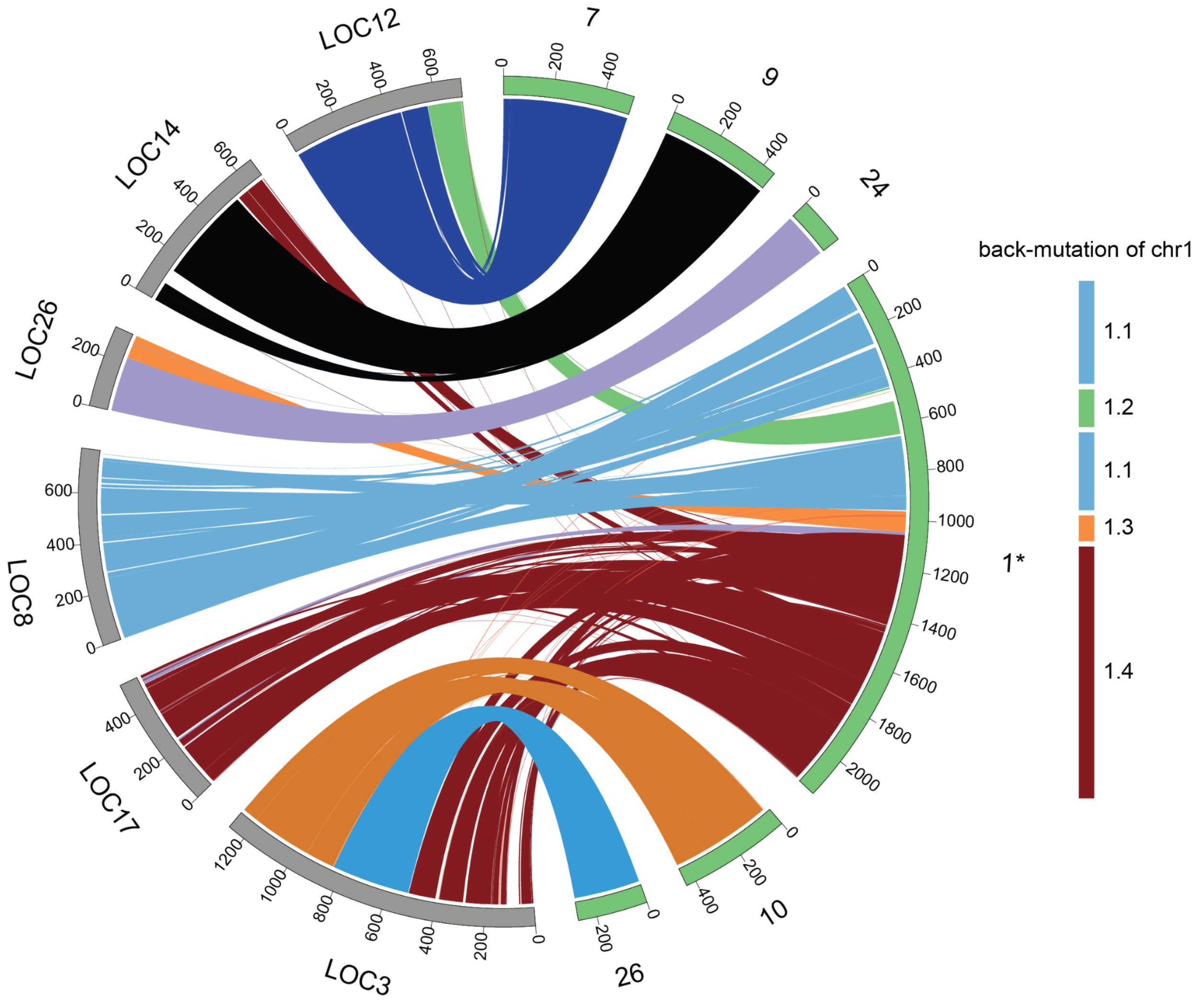


**Supplementary Figure 12. Chicken chr1 is homologous to multiple gar chromosomes.** A circos plot is used to show the macrosynteny. Each chromosome is scaled to the number of genes encoded by this chromosome and is arranged in a way to better visualize the correspondence of chromosomes. Each line represents one pair of homologous genes between chromosomes in different species, which is color-coded according to chicken and gar chromosomes (green for chicken and grey for gar). Chicken chr1 corresponds to multiple spotted gar chromosomes (e.g., LOC12), which in turn correspond to additional chicken chromosomes (e.g., chr7). Such a complex relationship suggests chicken- or gar-specific chromosomal fusions or fissions. After

polarization with the outgroup, i.e., elephant shark scaffold, we inferred that chicken chr1 has been subject to four fusions and thus manually split it back as chr1.1, 1.2, 1.3 and 1.4 to approximate gnathostome ancestral chromosomes (right panel). "*" marks the chromosome subject to back-mutation.

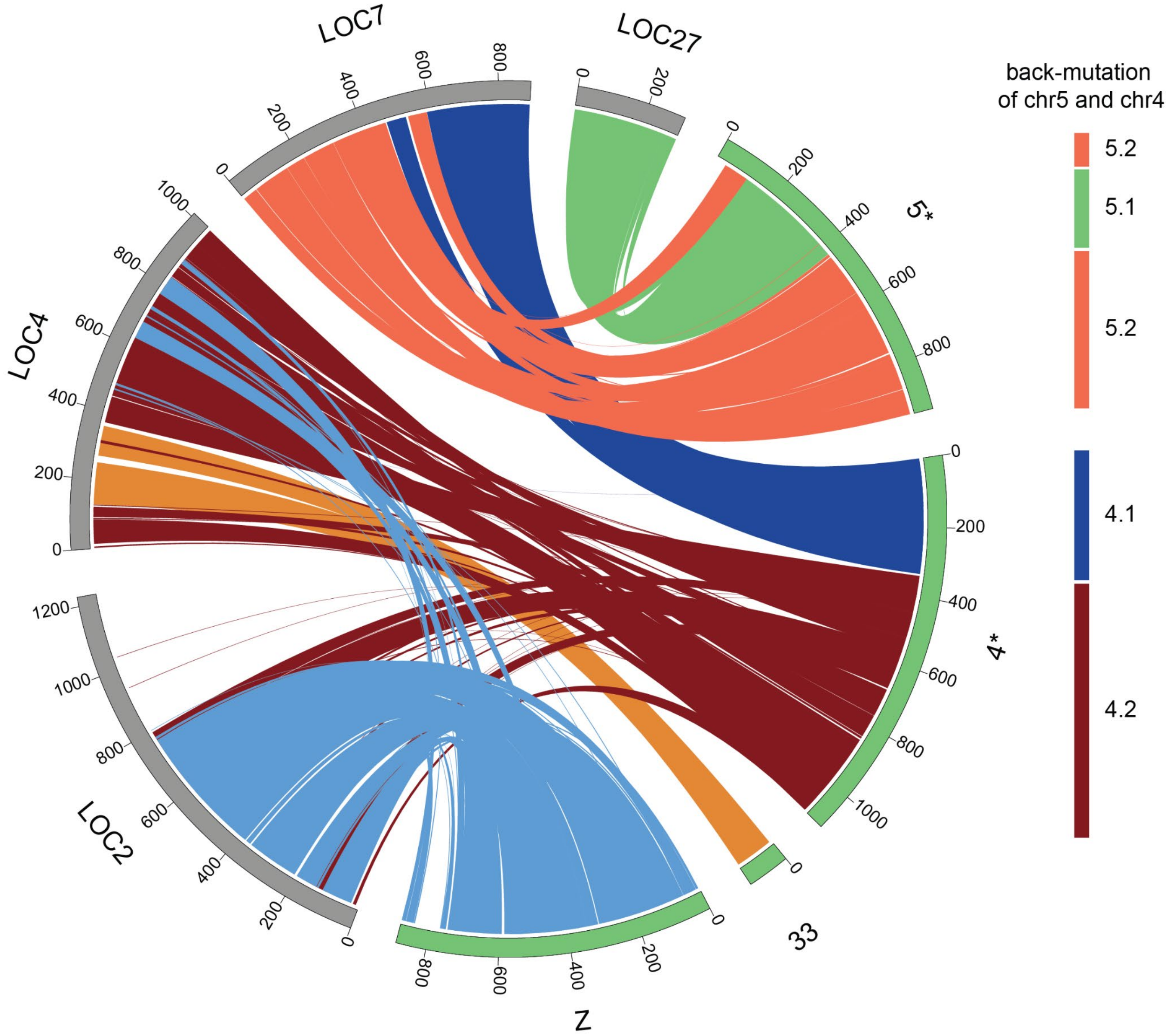


**Supplementary Figure 13. Both chicken chr4 and chr5 are homologous to two gar chromosomes, respectively.** The figure convention follows Supplementary Figure 12. Note that gar LOC2 and LOC4 are co-homologous with chicken chrZ and chr4.2.

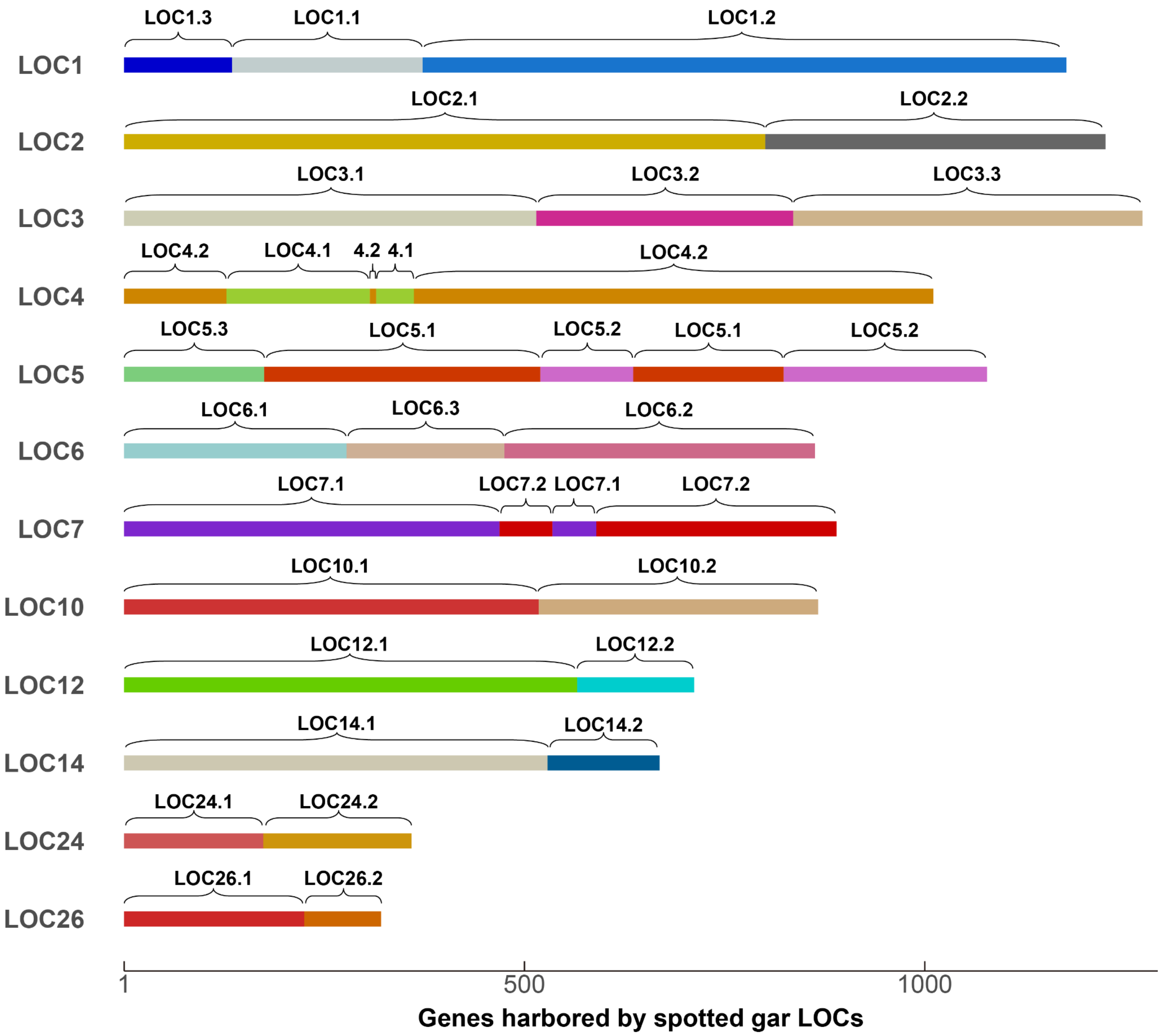


**Supplementary Figure 14. A schematic view of back-mutations in the gar genome.** Only spotted gar LOCs with lineage-specific fusions are shown. The length of each LOC is proportional to the number of genes it contains.

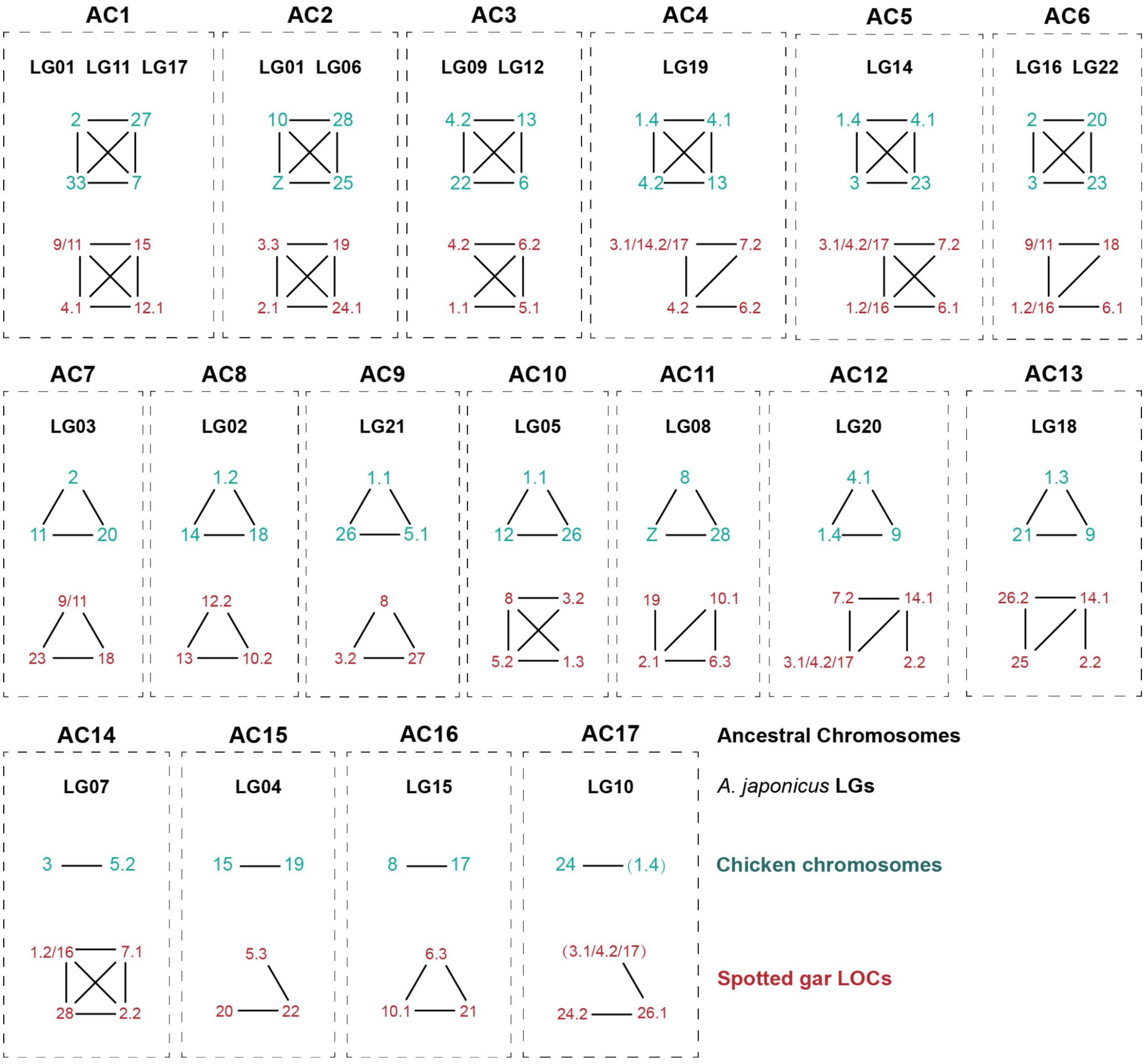


**Supplementary Figure 15. The homology between sea cucumber LGs and chromosomes of chicken and gar.** By examining whether a pair of chromosomes share a significant number of homologous genes, we divided chicken and gar chromosomes homologous to the same sea cucumber LGs in 17 groups, where chromosomes connected with lines are paralogous between each other. For sea cucumber LG10, chicken chr1.4 and spotted gar LOC3.1LOC14.2LOC17 are marked with parentheses because these two chromosomes are not significantly homologous to sea cucumber LG10. However, chicken chr24 is homologous to chr1.4 and spotted gar LOC26.1 is

homologous to LG3.1LG14.2LG17, while chr24, LOC24.2 and LOC26.1 are homologous to sea cucumber LG10. So chr1.4 and LOC3.1LOC14.2LOC17 appears to correspond to sea cucumber LG10 and the insignificance of correspondence can be due to the fact that chicken chr1.4 and gar LOC3.1LOC14.2LOC17 already correspond to sea cucumber LG14, LG19 and LG20. Actually, after excluding chicken or spotted gar genes mapping to sea cucumber LG14, LG19 and LG20, the relationship between chicken chr1.4 or spotted gar LOC3.1LOC14.2LOC17, and sea cucumber LG10 becomes significant or marginally significant ($q$ = 0.03, 0.06).

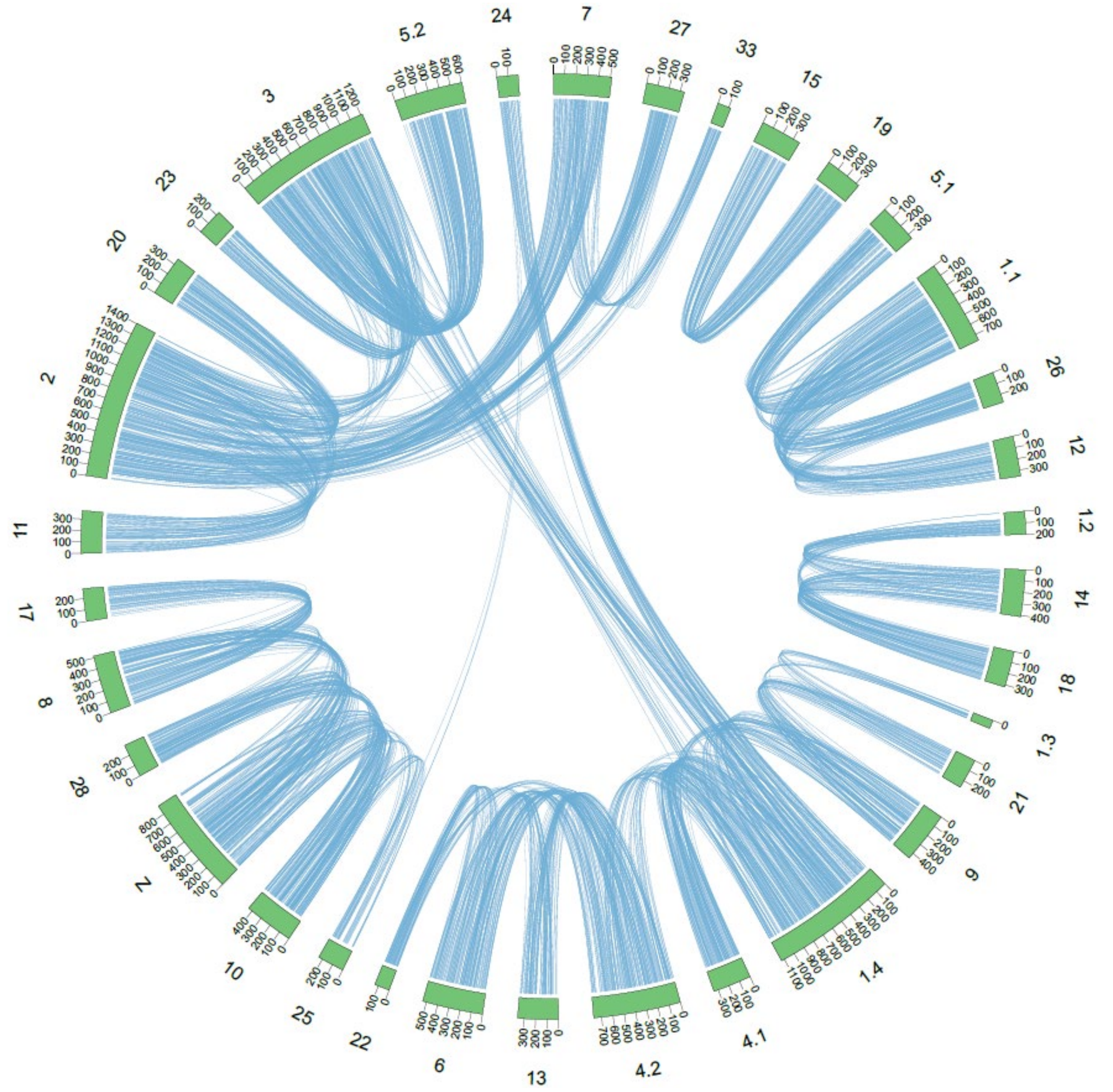


**Supplementary Figure 16. Extensive homology among chicken chromosomes.** To remove within-chromosome small scale duplications, only homologous gene pairs from homologous chromosome pairs are shown. This circos plot view supports the occurrence of at least one round of whole genome duplication.

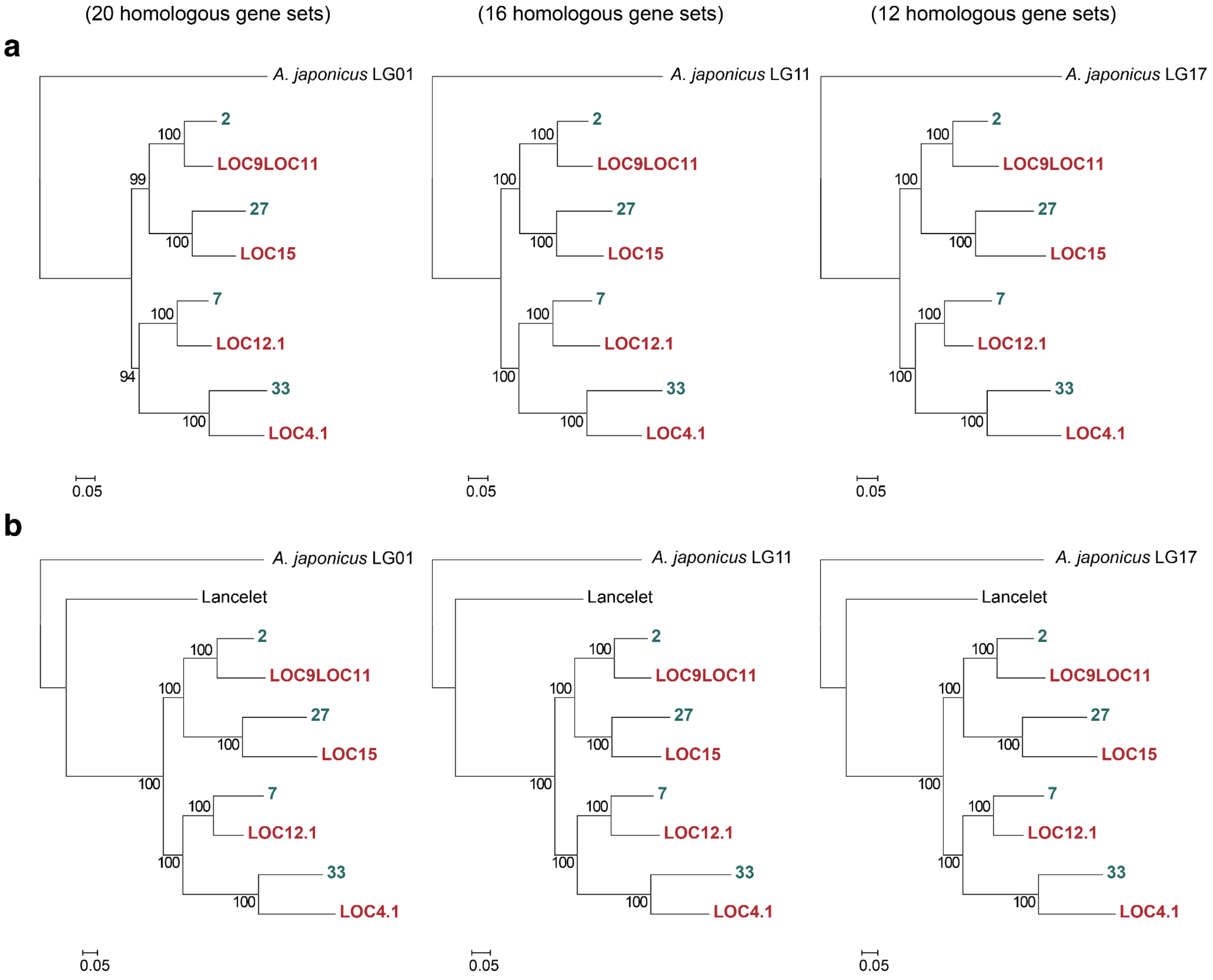


**Supplementary Figure 17. The phylogeny of chicken chromosomes chr2, 7, 27, and 33. a,** Chromosomal level trees with sea cucumber, *A. japonicus*, linkage groups LG01, LG11 and LG17 as outgroups. Due to gene loss after WGDs, only a small proportion of genes retains four duplicates in both chicken and spotted gar. This is why we only have 12 to 20 homologous gene sets applicable for each chromosomal level tree reconstruction. **b**, Same as **a** except that lancelet (*Branchiostoma belcheri*) homologous genes are added into each homologous gene set. Cyan and red denote chicken chromosomes; linkage groups from the spotted gar genome are denoted as LOCs. Bootstrap scores are shown along the internal branches and the protein divergence is measured with the unit as 0.05 (5%). With sea cucumber LG01, LG11 or LG17 as outgroups, the

same tree topologies were generated. The consensus tree of chicken chromosomes corresponds to (((chr2, chr27), (chr7, chr33)), LG01/LG11/LG17).

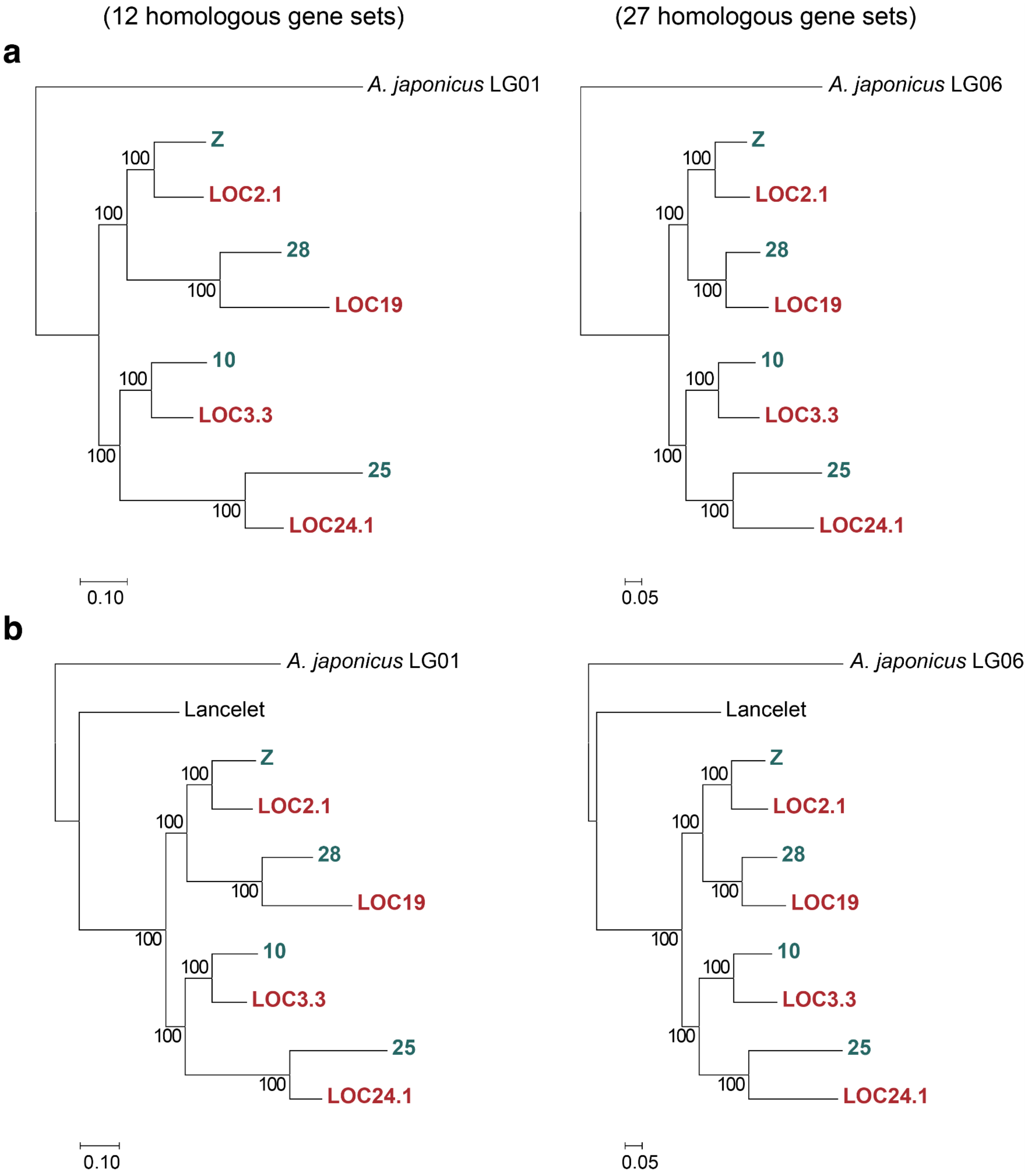


**Supplementary Figure 18. The phylogeny of chicken chromosomes chrZ, 28, 10, and 25.** This figure follows the same convention as Supplementary Figure 17. The consensus tree of chicken chromosomes appears to be (((chrZ, chr28), (chr10, chr25)), LG01/LG06).

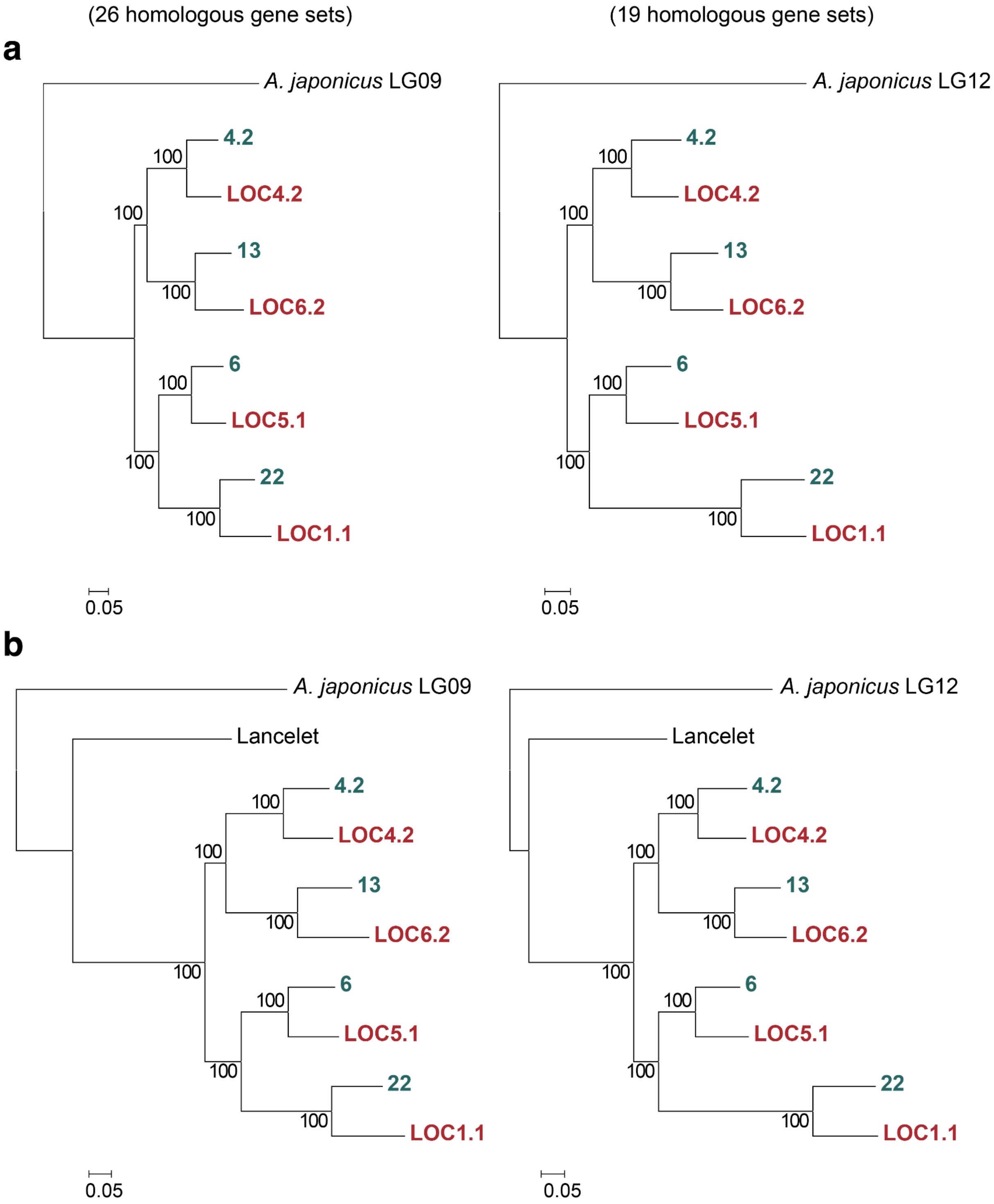


**Supplementary Figure 19. The phylogeny of chicken chromosomes chr4.2, 13, 6, and 22.** This figure follows the same convention as Supplementary Figure 17. The consensus tree of chicken chromosomes appears to be (((chr4.2, chr13), (chr6, chr22)), LG09/LG12).

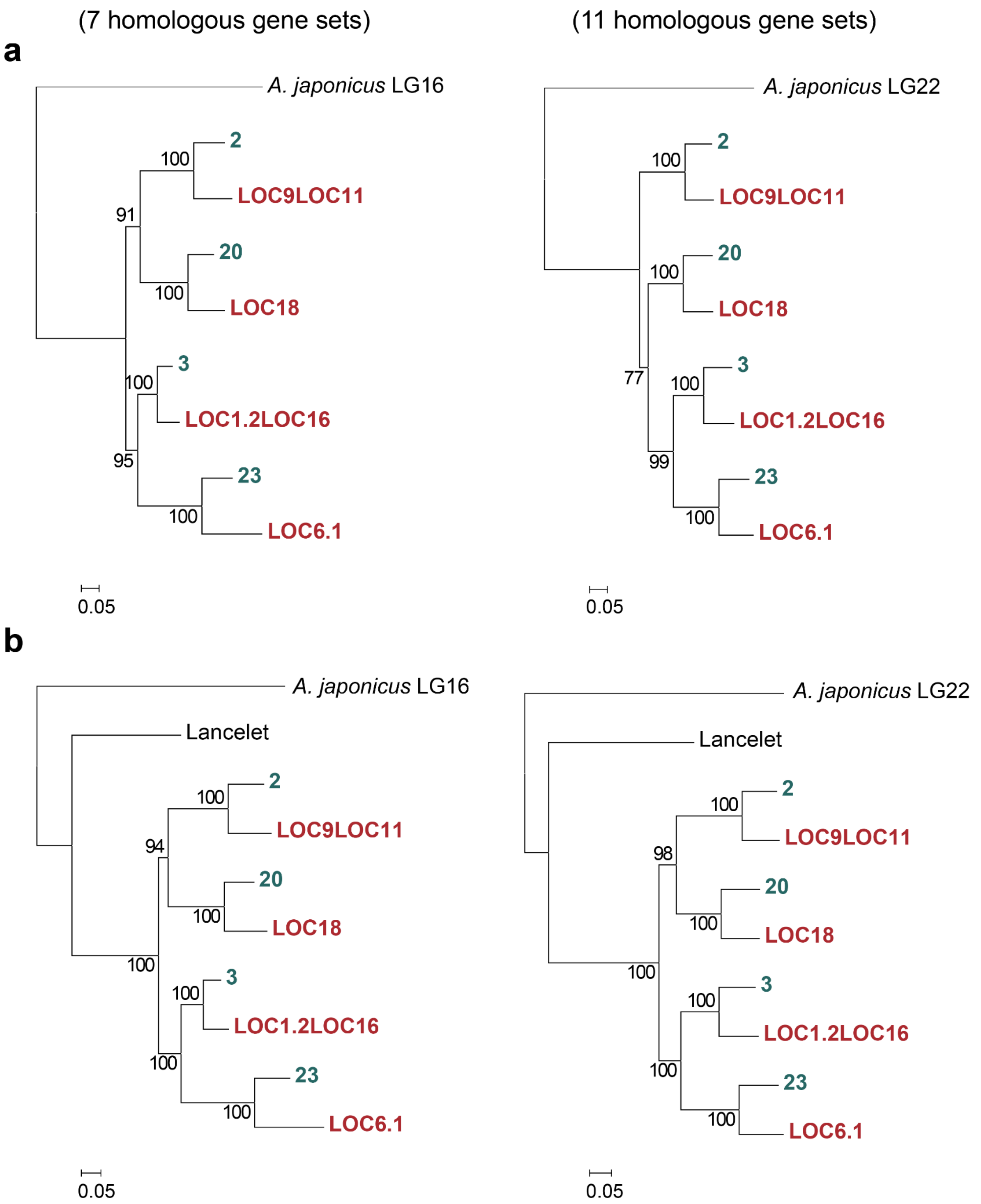


**Supplementary Figure 20. The phylogeny of chicken chromosomes chr2, 20, 3, and 23.** This figure follows the same convention as Supplementary Figure 17. The consensus tree of chicken chromosomes appears to be (((chr2, chr20), (chr3, chr23)), LG16/LG22).

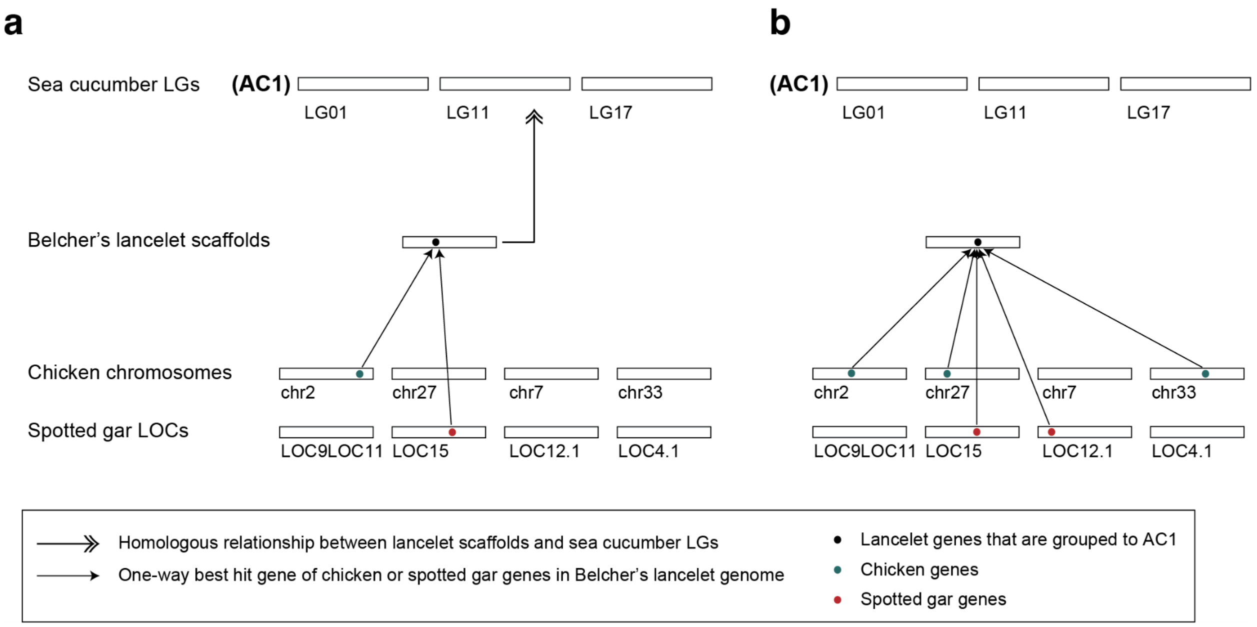


**Supplementary Figure 21. Two complementary strategies used to reconstruct the gene content of ACs based on amphioxus genes.** We took AC1 as an example, which corresponds to sea cucumber LG01/LG11/LG17; chicken chr2, 7, 27, and 33; and spotted gar LOC9LOC11, LOC15, LOC12.1, and LOC4.1 (Supplementary Table 9). **a,** For a lancelet scaffold that is homologous to sea cucumber LG01, LG11 or LG17, if at least one chicken gene from chr2, 7, 27, or 33 and at least one gene from spotted gar LOC9LOC11, LOC15, LOC12.1 or LOC4.1 are co-homologous with one gene encoded by this lancelet scaffold, we grouped the latter gene to AC1. **b,** If at least five genes from five different chromosome of chicken chr2, 7, 27, 33 or spotted gar LOC9LOC11, LOC15, LOC12.1, LOC4.1 have one lancelet gene homolog, we mapped the later gene to AC1 without consideration of the homology of the corresponding lancelet scaffold and sea cucumber LGs. For the second strategy, the reason that we require five genes is to ensure that this small gene family would have been generated by 2R WGDs in the ancestor of chicken and spotted gar.

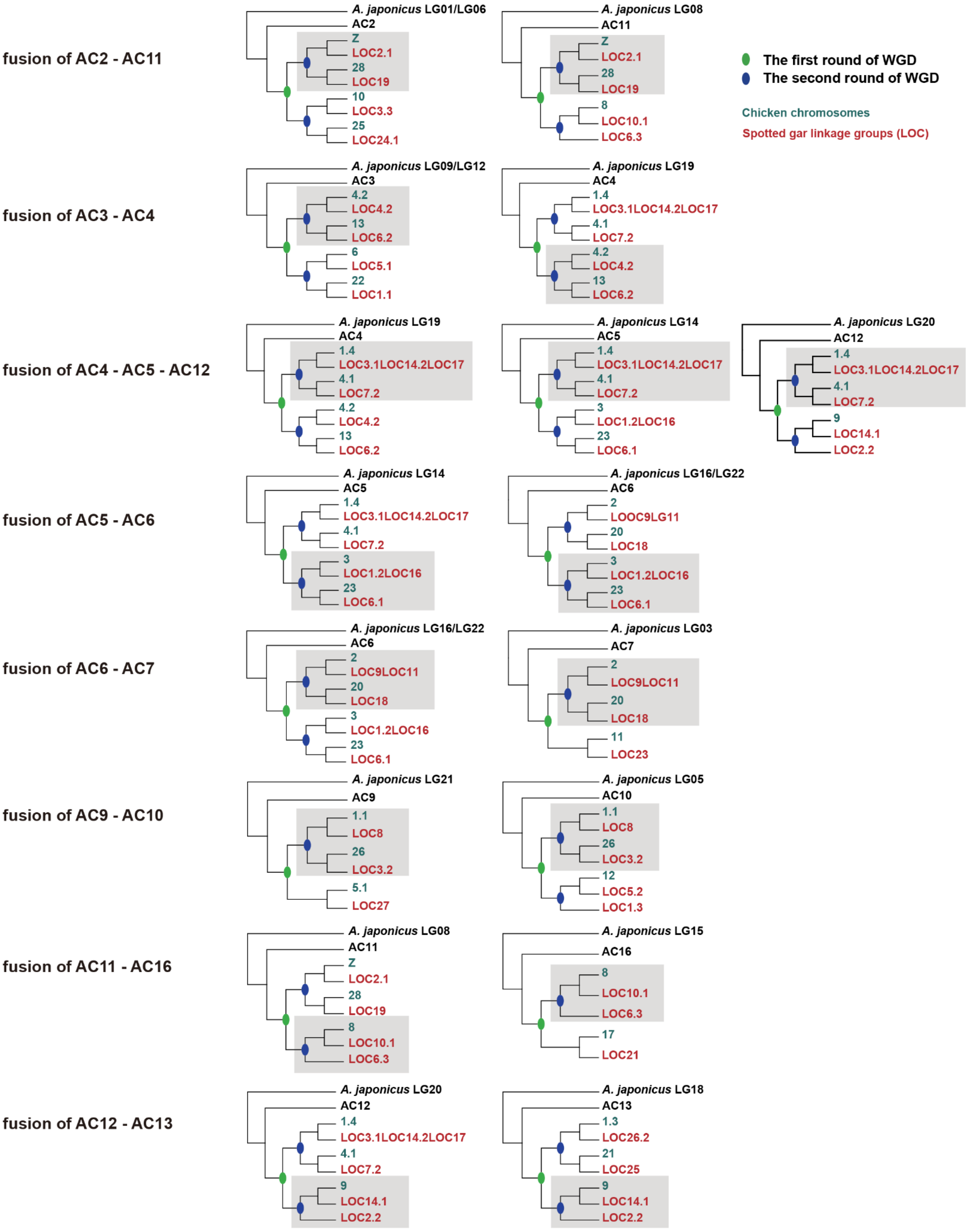
fusion of AC2 - AC11
A. japonicus LG01/LG06
AC2
Z
LOC2.1
28
LOC19
10
LOC3.3
25
LOC24.1
A. japonicus LG08
AC11
Z
LOC2.1
28
LOC19
8
LOC10.1
LOC6.3
The first round of WGD
The second round of WGD
Chicken chromosomes
Spotted gar linkage groups (LOC)
fusion of AC3 - AC4
A. japonicus LG09/LG12
AC3
4.2
LOC4.2
13
LOC6.2
6
LOC5.1
22
LOC1.1
A. japonicus LG19
AC4
1.4
LOC3.1LOC14.2LOC17
4.1
LOC7.2
4.2
LOC4.2
13
LOC6.2
fusion of AC4 - AC5 - AC12
A. japonicus LG19
AC4
1.4
LOC3.1LOC14.2LOC17
4.1
LOC7.2
4.2
LOC4.2
13
LOC6.2
A. japonicus LG14
AC5
1.4
LOC3.1LOC14.2LOC17
4.1
LOC7.2
3
LOC1.2LOC16
23
LOC6.1
A. japonicus LG20
AC12
1.4
LOC3.1LOC14.2LOC17
4.1
LOC7.2
9
LOC14.1
LOC2.2
fusion of AC5 - AC6
A. japonicus LG14
AC5
1.4
LOC3.1LOC14.2LOC17
4.1
LOC7.2
3
LOC1.2LOC16
23
LOC6.1
A. japonicus LG16/LG22
AC6
2
LOOC9LG11
20
LOC18
3
LOC1.2LOC16
23
LOC6.1
fusion of AC6 - AC7
A. japonicus LG16/LG22
AC6
2
LOC9LOC11
20
LOC18
3
LOC1.2LOC16
23
LOC6.1
A. japonicus LG03
AC7
2
LOC9LOC11
20
LOC18
11
LOC23
fusion of AC9 - AC10
A. japonicus LG21
AC9
1.1
LOC8
26
LOC3.2
5.1
LOC27
A. japonicus LG05
AC10
1.1
LOC8
26
LOC3.2
12
LOC5.2
LOC1.3
fusion of AC11 - AC16
A. japonicus LG08
AC11
Z
LOC2.1
28
LOC19
8
LOC10.1
LOC6.3
A. japonicus LG15
AC16
8
LOC10.1
LOC6.3
17
LOC21
fusion of AC12 - AC13
A. japonicus LG20
AC12
1.4
LOC3.1LOC14.2LOC17
4.1
LOC7.2
9
LOC14.1
LOC2.2
A. japonicus LG18
AC13
1.3
LOC26.2
21
LOC25
9
LOC14.1
LOC2.2

**Supplementary Figure 22. The eight post-1R fusions proposed based on chromosomal phylogenetic trees.** The products of the fused chromosomes after the second round of gnathostome WGD are marked in grey blocks.

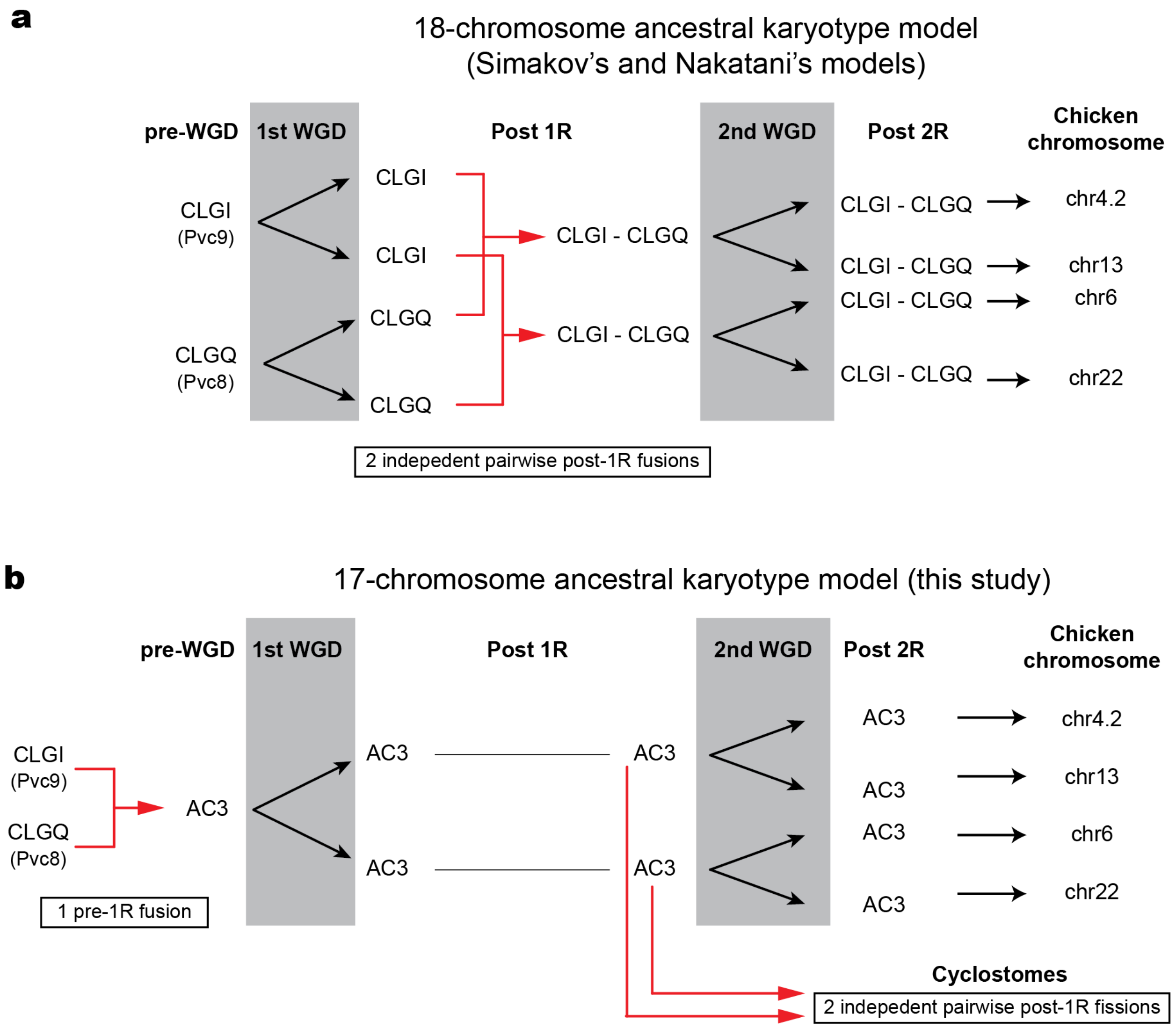


**Supplementary Figure 23. Two hypothetical hypotheses explaining the origin of chicken chr4.2, 13, 6 and 22. a,** Two pairwise post-1R fusion events involving two pairs of the same ohnologous chromosomes are hypothesised in the 18-CLG and 18-PVC models[123,124]. **b,** A pre-1R fusion event is hypothesised in our 17-AC model, where AC3 is equivalent to a fusion event of CLGI and CLGQ of Simakov's 17-CLG model[122] (same as Pvc9 and Pvc8, respectively, in the 18-PVC model[124]). Two pairwise post-1R fissions are inferred in a cyclostome stem in this model.

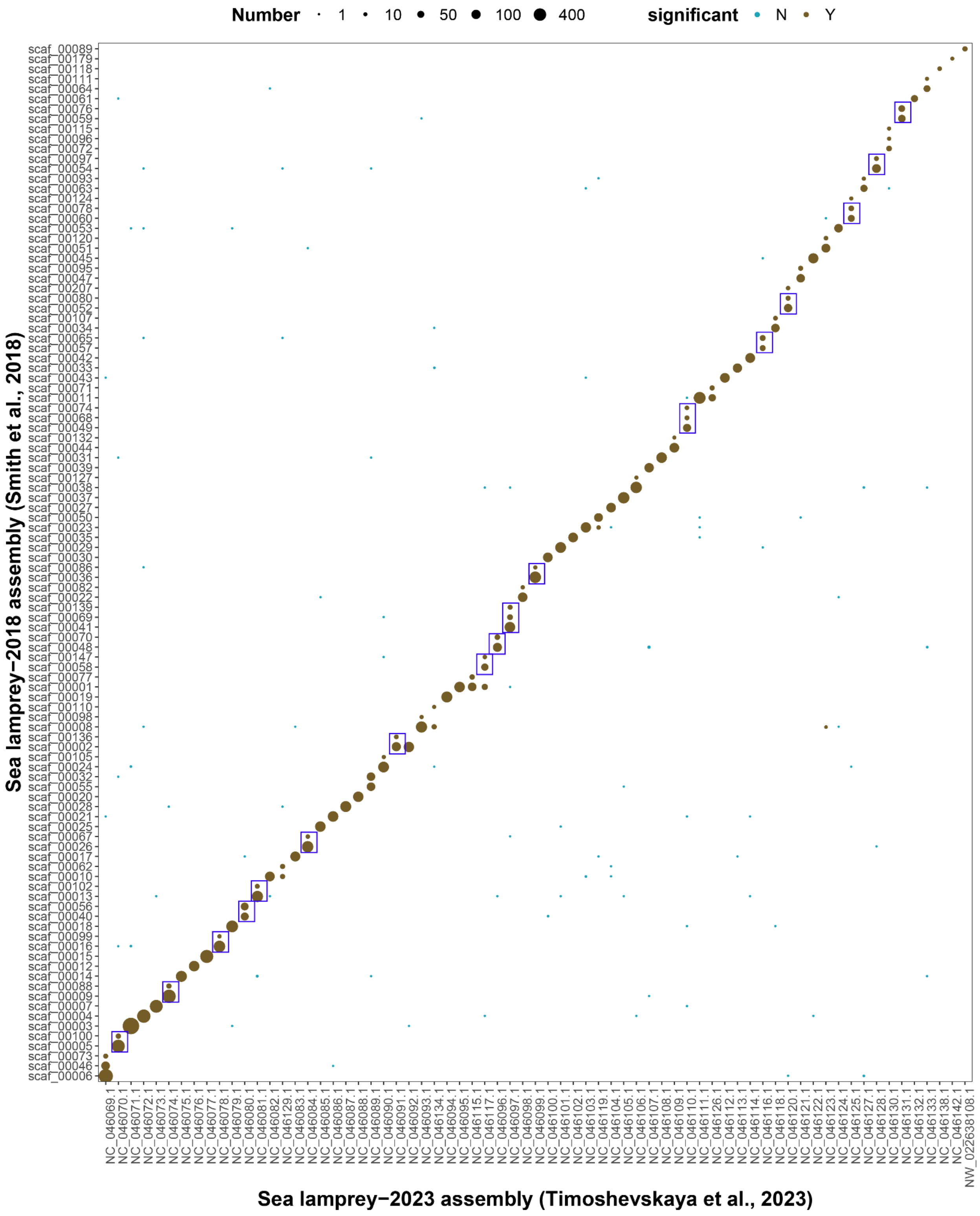

Number
1
10
50
100
400
significant
N
Y
Sea lamprey−2018 assembly (Smith et al., 2018)
scaf_00089
scaf_00179
scaf_00118
scaf_00111
scaf_00064
scaf_00061
scaf_00076
scaf_00059
scaf_00115
scaf_00096
scaf_00072
scaf_00097
scaf_00054
scaf_00093
scaf_00063
scaf_00124
scaf_00078
scaf_00060
scaf_00053
scaf_00120
scaf_00051
scaf_00045
scaf_00095
scaf_00047
scaf_00207
scaf_00080
scaf_00052
scaf_00107
scaf_00034
scaf_00065
scaf_00057
scaf_00042
scaf_00033
scaf_00043
scaf_00071
scaf_00011
scaf_00074
scaf_00068
scaf_00049
scaf_00132
scaf_00044
scaf_00031
scaf_00039
scaf_00127
scaf_00038
scaf_00037
scaf_00027
scaf_00050
scaf_00023
scaf_00035
scaf_00029
scaf_00030
scaf_00086
scaf_00036
scaf_00082
scaf_00022
scaf_00139
scaf_00069
scaf_00041
scaf_00070
scaf_00048
scaf_00147
scaf_00058
scaf_00077
scaf_00001
scaf_00019
scaf_00110
scaf_00098
scaf_00008
scaf_00136
scaf_00002
scaf_00105
scaf_00024
scaf_00032
scaf_00055
scaf_00020
scaf_00028
scaf_00021
scaf_00025
scaf_00067
scaf_00026
scaf_00017
scaf_00062
scaf_00010
scaf_00102
scaf_00013
scaf_00056
scaf_00040
scaf_00018
scaf_00099
scaf_00016
scaf_00015
scaf_00012
scaf_00014
scaf_00088
scaf_00009
scaf_00007
scaf_00004
scaf_00003
scaf_00100
scaf_00005
scaf_00073
scaf_00046
scaf_00006
NC_046069.1
NC_046070.1
NC_046071.1
NC_046072.1
NC_046073.1
NC_046074.1
NC_046075.1
NC_046076.1
NC_046077.1
NC_046078.1
NC_046079.1
NC_046080.1
NC_046081.1
NC_046082.1
NC_046129.1
NC_046083.1
NC_046084.1
NC_046085.1
NC_046086.1
NC_046087.1
NC_046088.1
NC_046089.1
NC_046090.1
NC_046091.1
NC_046092.1
NC_046093.1
NC_046134.1
NC_046094.1
NC_046095.1
NC_046115.1
NC_046117.1
NC_046096.1
NC_046097.1
NC_046098.1
NC_046099.1
NC_046100.1
NC_046101.1
NC_046102.1
NC_046103.1
NC_046119.1
NC_046104.1
NC_046105.1
NC_046106.1
NC_046107.1
NC_046108.1
NC_046109.1
NC_046110.1
NC_046111.1
NC_046126.1
NC_046112.1
NC_046113.1
NC_046114.1
NC_046116.1
NC_046118.1
NC_046120.1
NC_046121.1
NC_046122.1
NC_046123.1
NC_046124.1
NC_046125.1
NC_046127.1
NC_046128.1
NC_046130.1
NC_046131.1
NC_046132.1
NC_046133.1
NC_046138.1
NC_046142.1
NW_022638108.1
Sea lamprey−2023 assembly (Timoshevskaya et al., 2023)

**Supplementary Figure 24. 17 manually fused lamprey scaffolds are validated in the updated lamprey assembly.** This figure summarises the homology mapping between two releases of the sea lamprey genome[143,144]. We previously detected 17 cases of scaffold pairs or trios in the 2018 release when comparing with a meiotic map of the Pacific lamprey *Entosphenus tridentatus*, also provided by Smit et al.[143], each case of which should be derived from the same chromosome. These 17 cases are validated in the 2020 release. Notably, a dozen pairs or trios of scaffolds should be assembled together too, for example, scaf_00073, scaf_00046 and scaf_00006 represents fragments derived from NC_046069.1 (the left bottom corner of the figure).

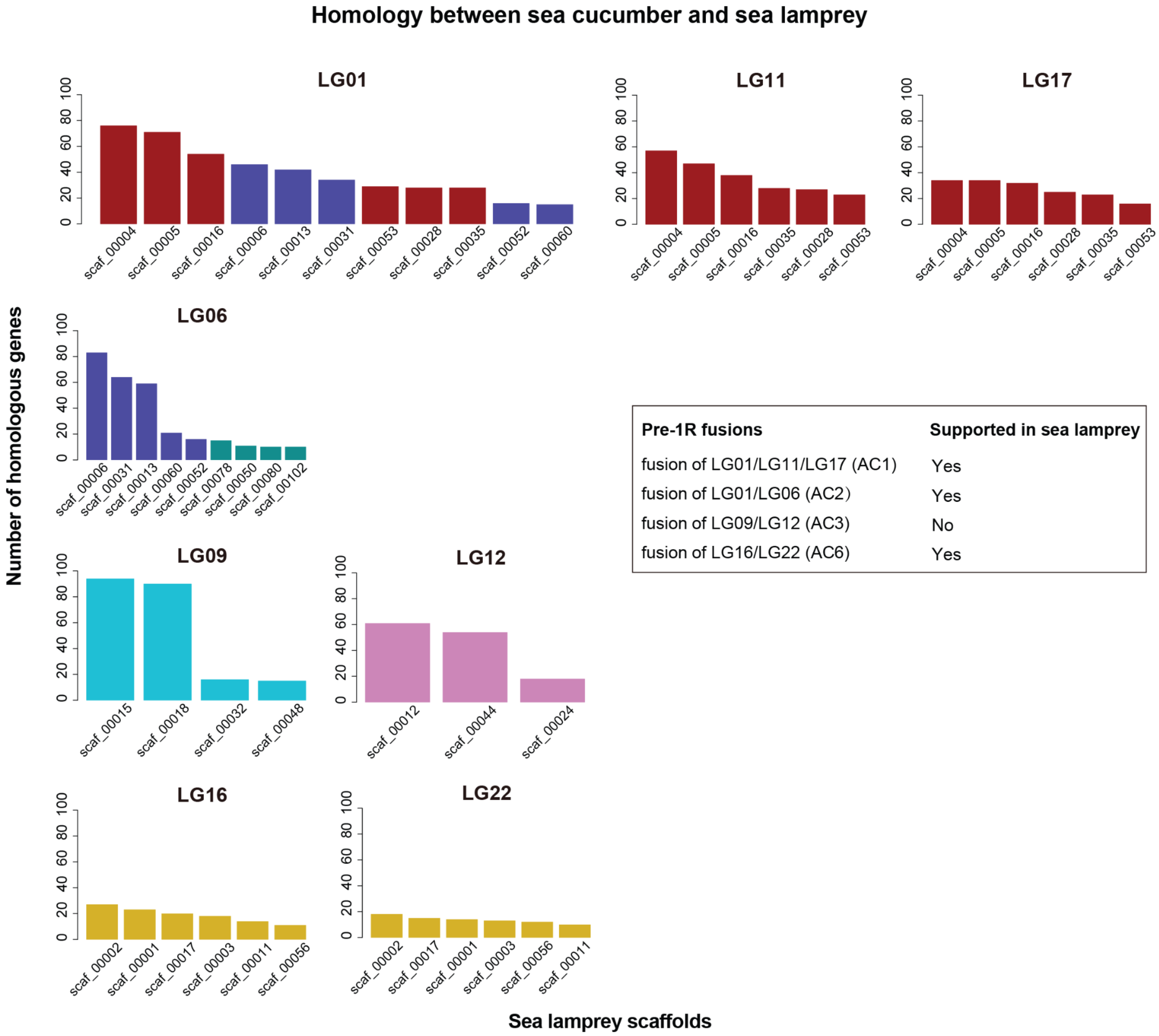


**Supplementary Figure 25. The sea lamprey genome bears signatures of three out of four vertebrate pre-1R fusion events.** Four pre-1R fusions of ancestral chromosomal regions, corresponding to LG01/LG11/LG17, LG01/LG06, LG09/LG12, and LG16/LG22 of the sea cucumber genome, were proposed based on the analyses of sea cucumber, lancelet, chicken and gar genomes. Herein, sea lamprey scaffolds homologous to sea cucumber LG01, LG06, LG11, LG17, LG09, LG12, LG16 and LG12 are shown. They correspond to AC1 (LG01/LG11/LG17), AC2 (LG01/LG06), AC3 (LG09/LG12), and AC6 (LG16/LG22) respectively. Sea lamprey supra-

scaffolds supporting fusions (LG01/LG11/LG17, LG01/LG06 and LG16/LG22) are marked in the same colour.

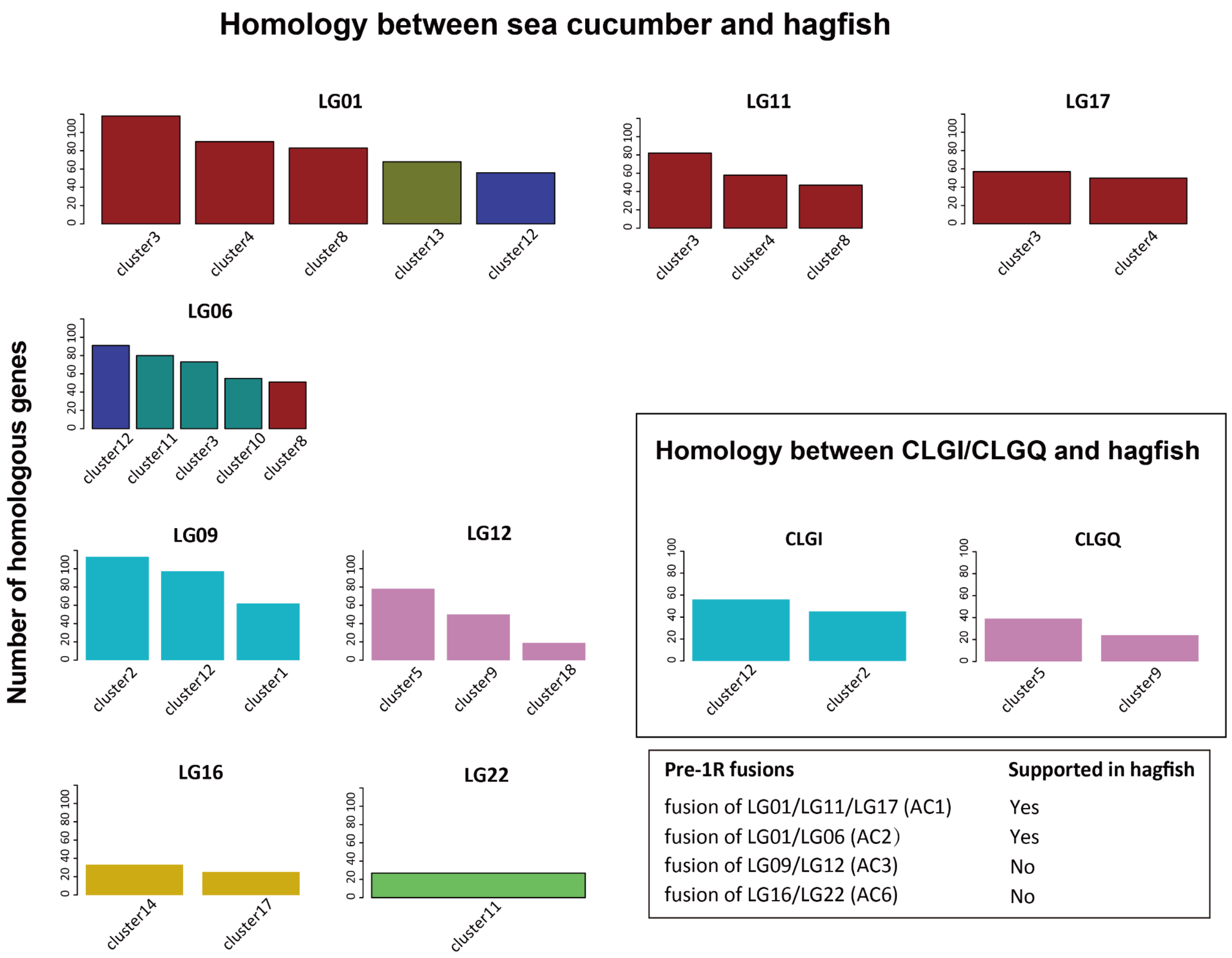


| Pre-1R fusions | Supported in hagfish |
|---|---|
| fusion of LG01/LG11/LG17 (AC1) | Yes |
| fusion of LG01/LG06 (AC2) | Yes |
| fusion of LG09/LG12 (AC3) | No |
| fusion of LG16/LG22 (AC6) | No |

**Supplementary Figure 26. The hagfish genome bears signatures of two out of four vertebrate pre-1R fusion events.** Four pre-1R fusions of ancestral chromosomal regions, corresponding to LG01/LG11/LG17, LG01/LG06, LG09/LG12, and LG16/LG22 of the sea cucumber genome, were proposed based on the analyses of sea cucumber, lancelet, chicken and gar genomes. Herein, hagfish Hi-C clusters (pseudo-chromosomes) homologous to sea cucumber's LG01, LG06, LG11, LG17, LG09, LG12, LG16 and LG12 are shown. They correspond to AC1 (LG01/LG11/LG17), AC2 (LG01/LG06), AC3 (LG09/LG12), and AC6 (LG16/LG22) respectively. Hagfish Hi-C clusters supporting fusions (LG01/LG11/LG17 and LG01/LG06) are marked in the same colour.

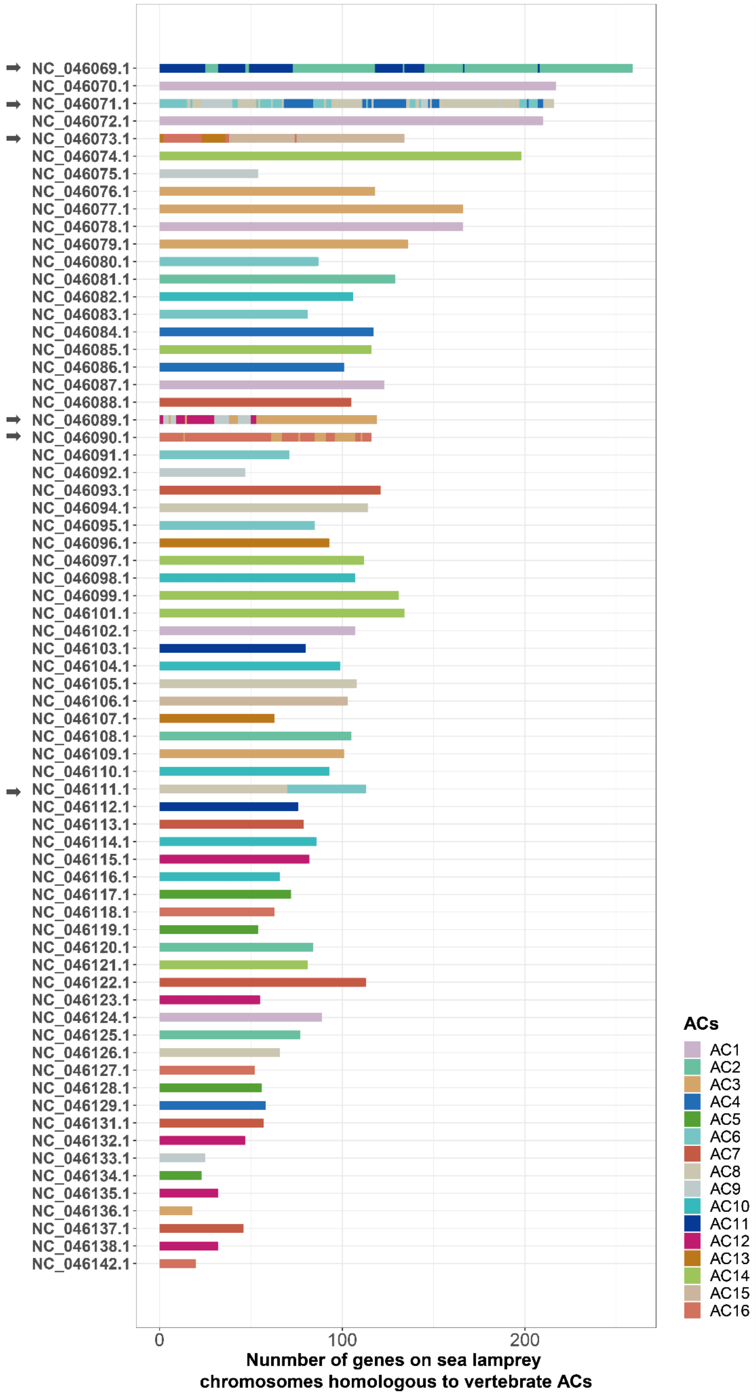


**Supplementary Figure 27. Contributions of vertebrate ACs to the chromosome-level assembly of the sea lamprey genome.** Sea lamprey chromosomes (from the latest chromosome-level[144]) are generally homologous to one single AC except six chromosomes, which are marked with an arrow.

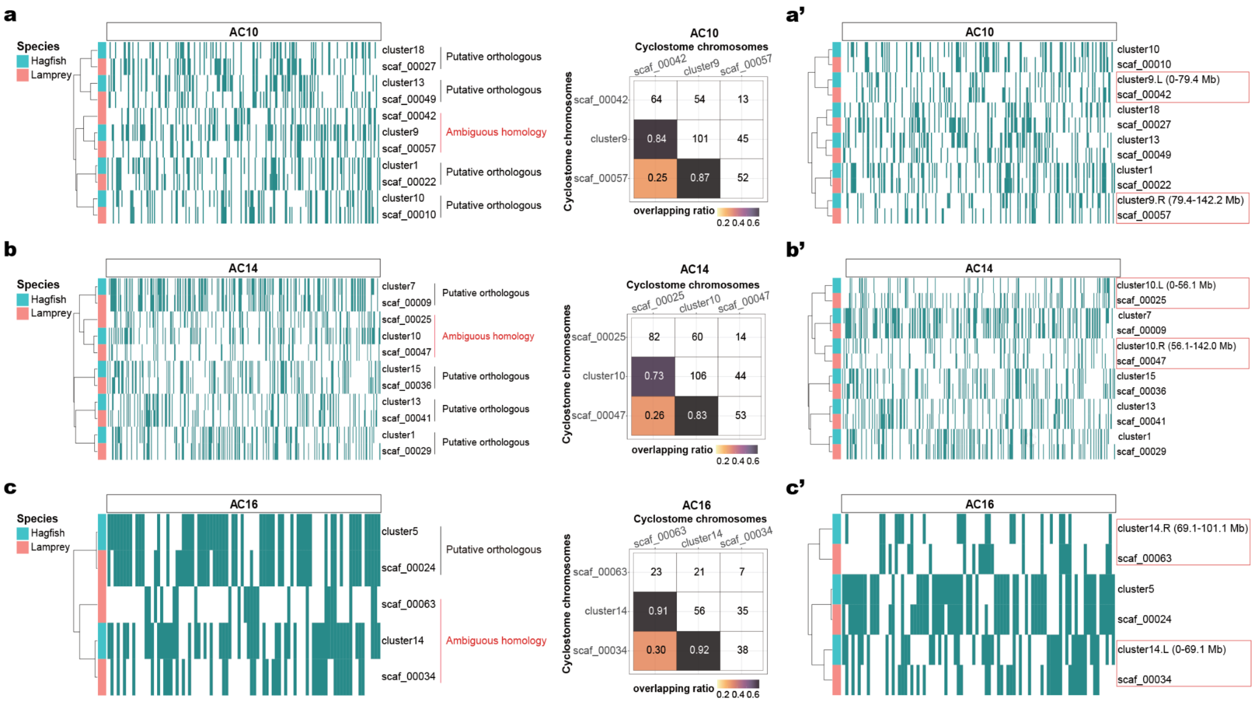


**Supplementary Figure 28. Three cases of hagfish-specific fusion of paralogous chromosomes were identified and back-mutated. a,** The left panel shows that hagfish Hi-C cluster 9 is most similar to sea lamprey scaffolds 42 and 57 in terms of AC10 derived homologs, while the right panel shows that the two lamprey scaffolds are likely paralogous suggested by their decent overlapping ratio. **a',** After manually splitting of hagfish Hi-C cluster 9 into two parts (split at 79.4 Mb) into cluster9.L and cluster9.R, these cluster well separately with sea lamprey scaffolds 42 and 57, respectively, indicating that hagfish cluster9 is the result of a fusion of two paralogous chromosomes. **b, b', c** and **c'** show two further examples with chromosomes deriving from AC10 and AC16. Another example is that of *Hox*-containing hagfish Hi-C cluster 3 (see Extended Data Figure 4e).

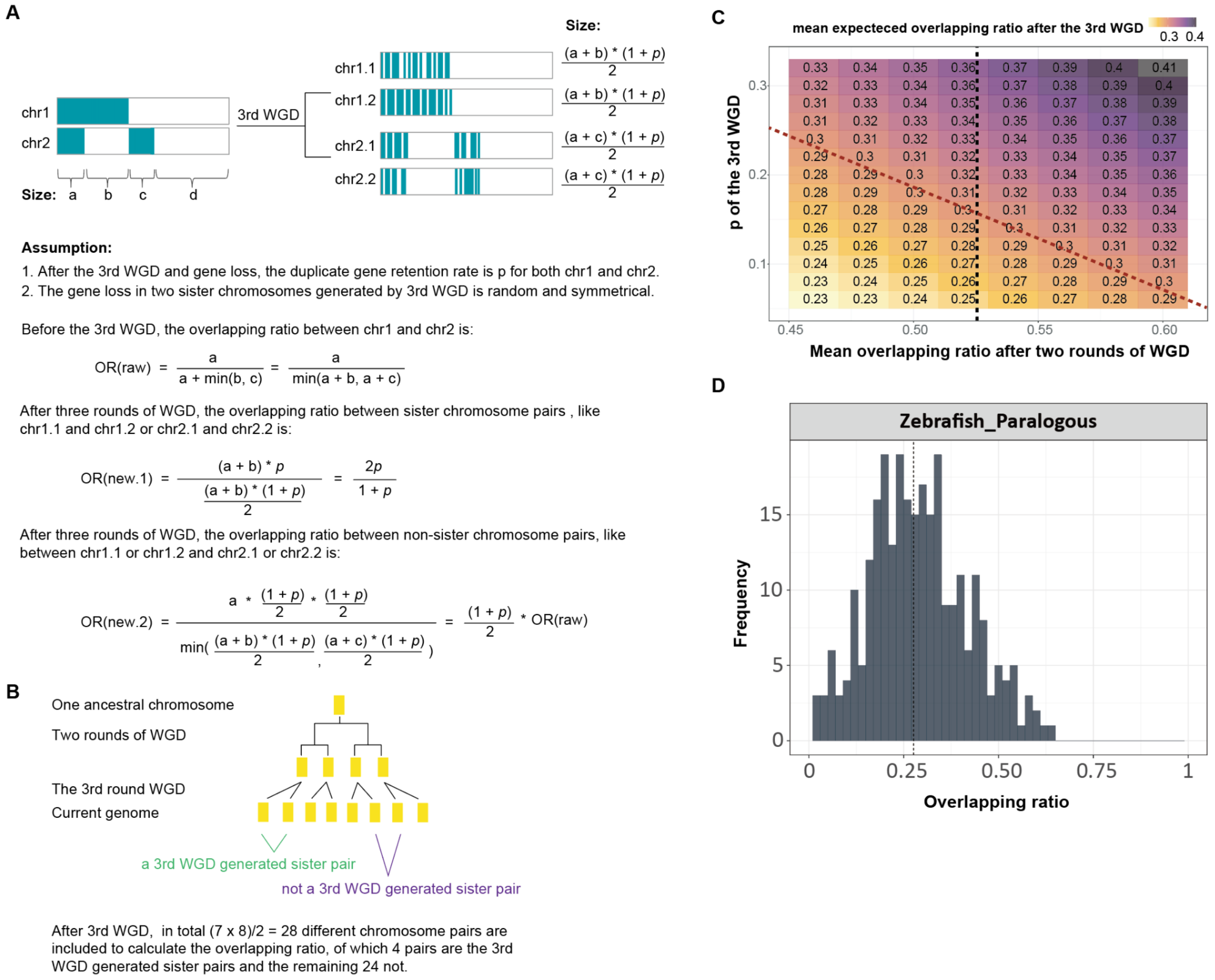


**Supplementary Figure 29. More rounds of WGDs cause the continuous decrease of overlapping ratio. a,** With one more round of WGD, the overlapping ratio drops with a rate (1+p)/2, where p is equal to the percentage of retained duplicates. **b,** A schema on how a third round of WGD changes the number of sister chromosomes and thus the calculation of average overlapping ratio between paralogous chromosomes. **c,** The mean overlapping ratio after the third round of WGD is calculated based on a different combination of parameters including the duplicate retention rate (p) and mean overlapping ratio after two rounds of WGD. A black dashed line indicates the observed median overlapping ratio (0.52) of paralogous chromosomes in

gnathostomes shaped by two rounds of WGD, while a red dashed line indicates the observed median overlapping ratio (0.30) in cyclostomes. **d,** Distribution of *OR* values for putative paralogous chromosomes in the zebrafish *Danio rerio* genome. A dashed line marks a median value = 0.28

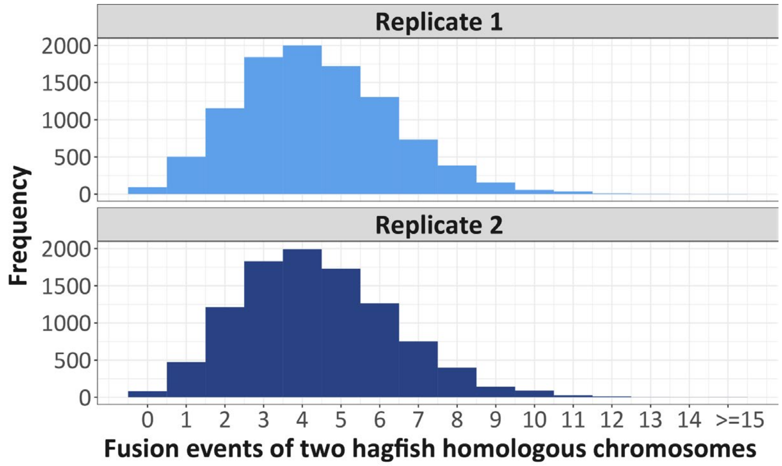


**Supplementary Figure 30.** Frequency distribution of fusion events of two hagfish paralogous chromosomes. We randomly fused hagfish chromosomes and counted how many times that paralogous chromosomes were fused.

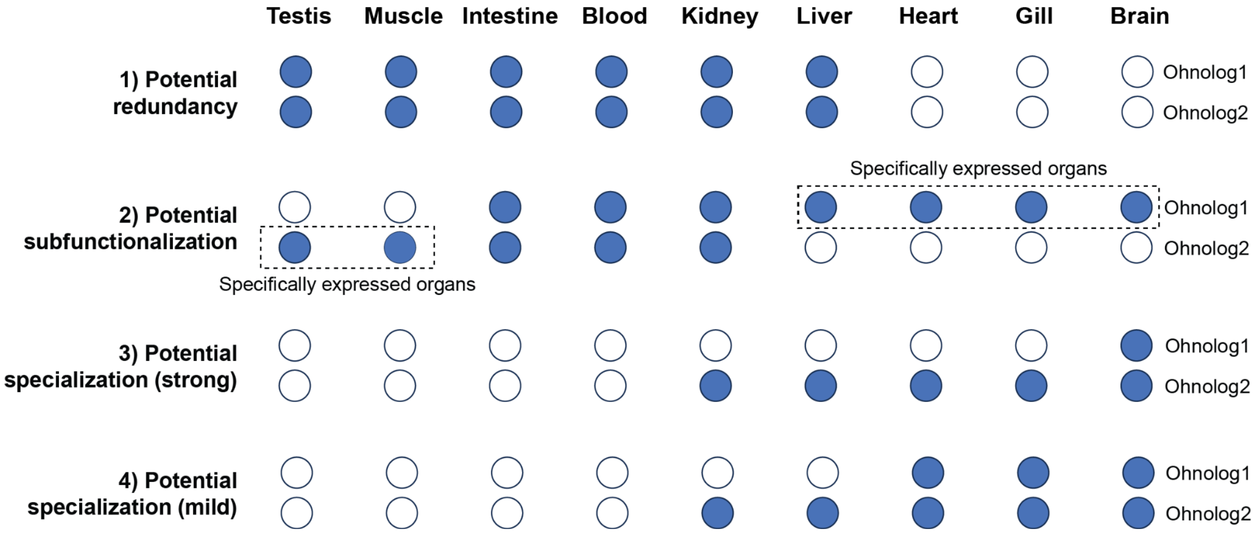


**Supplementary Figure 31. Possible fates of ohonologs after WGD according to their expression domains.** The filled and blank circles represent on and off expressional states, respectively, in each tissue. Ohnologous genes with the same expressional tissues are classified as potentially redundant (1); pairs of ohnologs in which each of them is specifically expressed in a subset of tissues are considered as potentially subfunctionalized (2); and ohnolog families in which one of the ohnologs is expressed in a subset of the expressional domains of the other corresponding ohnolog, is considered potentially specialized (3) and (4). We consider it a 'potential strong specialization' (3) case when the ohnolog with the narrower expression pattern is transcribed in $<$ 40% of domains than the ohnolog with the broader expression pattern; we consider it a 'potential mild specialization' (4) case when the ohnolog with the narrower expression pattern is transcribed in $\geq$ 40% of domains than the ohnolog with the broader expression pattern.

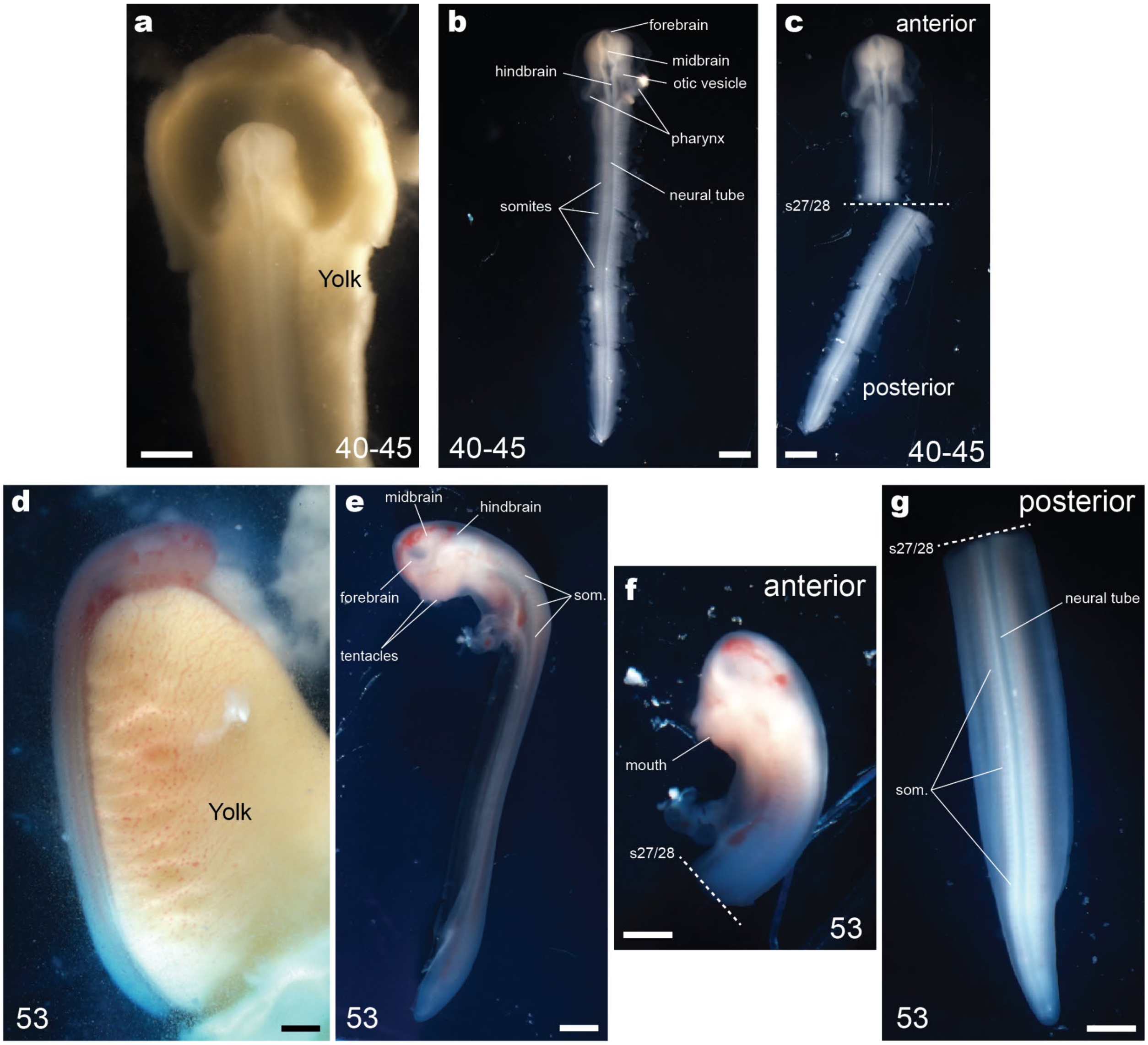


**Supplementary Figure 32. Hagfish embryos used for ATAC-seq. a-c,** Embryo of the inshore hagfish *E. burgeri* at stage Dean ~40-45 extracted from the eggshell and assayed for ATAC-seq. Yolk was removed almost completely in (**a**), and completely in (**b**,**c**). Key anatomical features are labeled. The embryo was divided into anterior and posterior halves (**c**) at the level between somites 27 and 28 (dashed line) and each was assayed separately. Only one embryo was assayed due to the rarity of embryological material of hagfish species. **d-g**, Embryo at stage Dean 53. As with before, we divided the embryo into anterior (**f**) and posterior (**g**) halves at an equivalent A-P level (somites

27 and 28, dashed lines). Only one embryo was assayed due to the rarity of embryological material of hagfish species. Staging of both embryos was performed according to a previous study[159]. Scale bars, 1 mm.

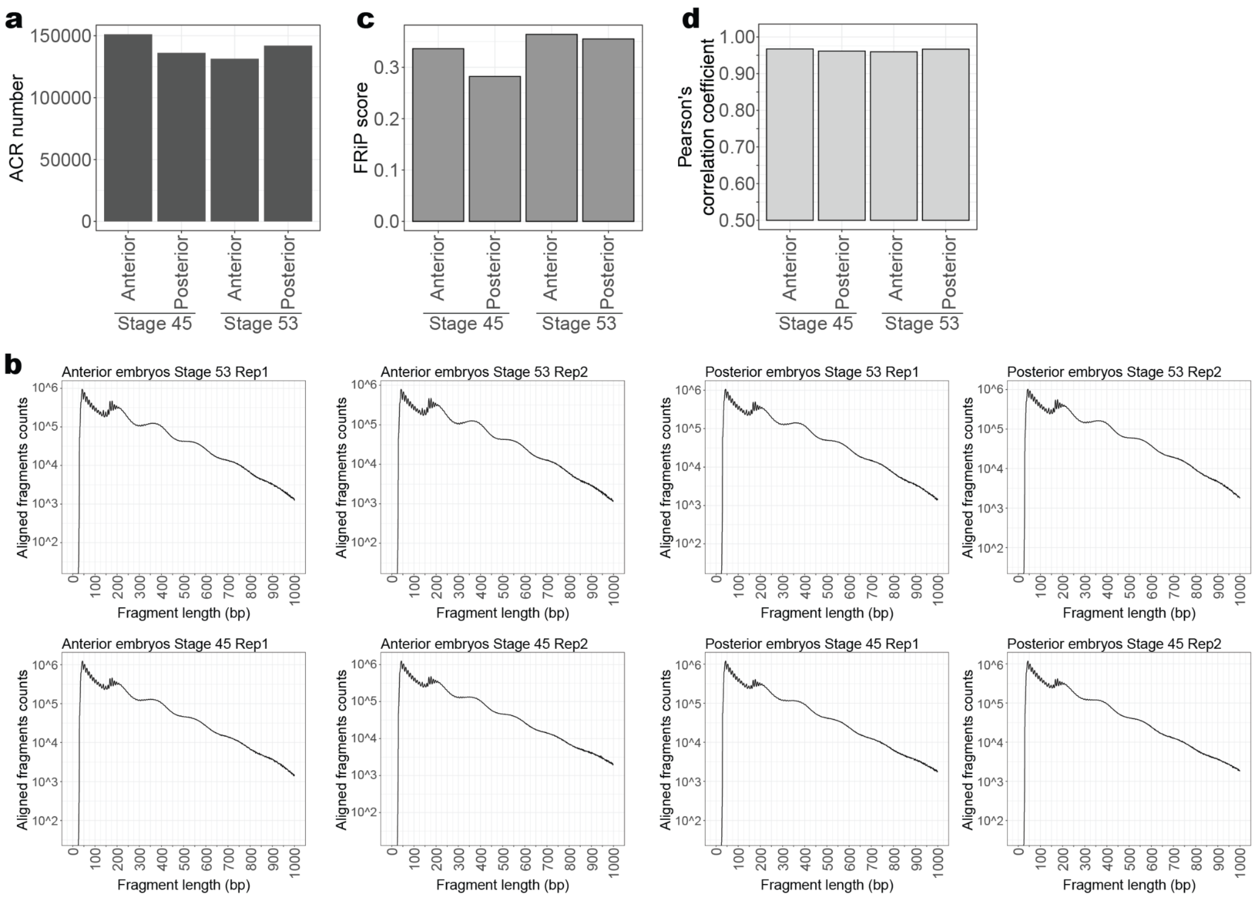


**Supplementary Figure 33. Validation of ATAC-seq data of hagfish embryos. a,** Number of identified ACRs in each sample. **b,** Distributions of fragment size of properly paired aligned reads in each replicate data. **c,** A fraction of all aligned reads in the called ACRs (FRiP score[207]). **d,** The Pearson correlation coefficients of ATAC-seq signal intensities (log10-RPM; reads per million mapped reads) of ACRs between the technical replicates at different samples.

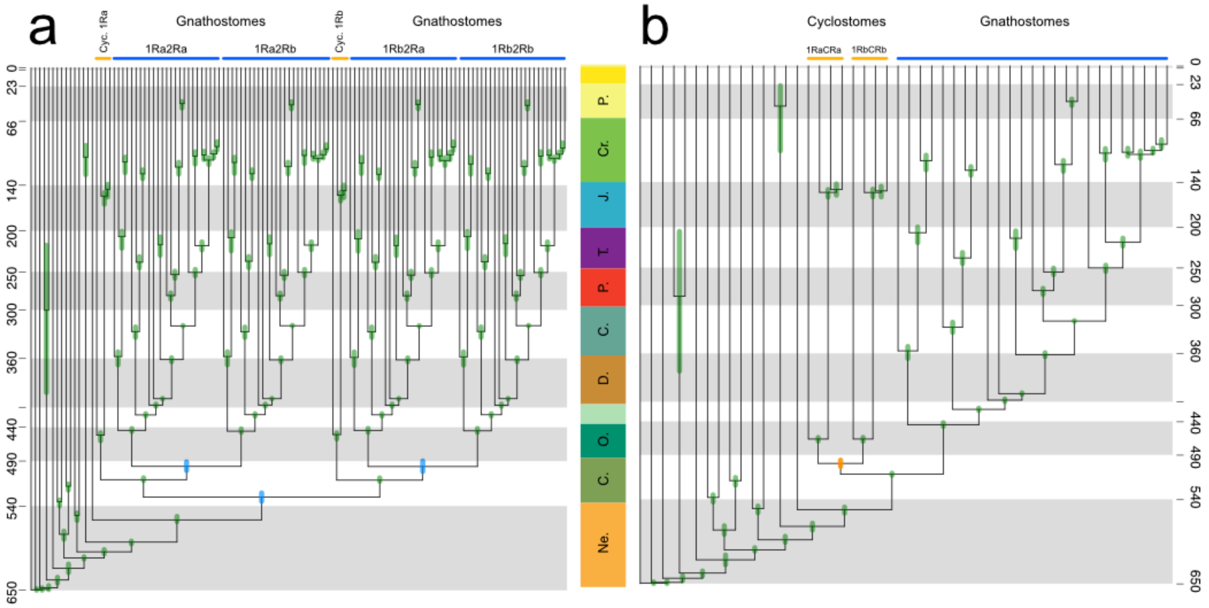


**Supplementary Figure 34. Dating Whole Genome Duplication events. a**, Dating using a multigene alignment focussed on the 1R and 2R events (blue node bars). **b**, Dating using a multigene alignment focussed on the CR event (orange node bar). The ontology used for the paralogy groups reflects the duplication history of the genes, e.g. 1Ra2Rb represents paralogy group a from 1R and paralogy group b (of 1Ra) from 2R. 1R and 2R are interpreted as autopolyploidy and allopolyploidy events, respectively, and so while the 1R estimate is for the WGD event itself, the 2R estimate is a maximum constraint on WGD timing.
