# Supplementary material for: Hagfish genome elucidates vertebrate whole-genome duplication events and their evolutionary consequences
Source: Nat Ecol Evol. 2024 Jan 12;8(3):519–35. doi: 10.1038/s41559-023-02299-z (PMC10927551; doi:10.1038/s41559-023-02299-z)
Supplement: Supplementary file 2 — Reporting Summary [file 41559_2023_2299_MOESM2_ESM.pdf]

nature portfolio

Corresponding author(s): Juan Pascual-Anaya, Yong E. Zhang, Philip C. J. Donoghue, Wen Wang, Fergal J. Martin

Last updated by author(s): Nov 28, 2023

Reporting Summary

Nature Portfolio wishes to improve the reproducibility of the work that we publish. This form provides structure for consistency and transparency in reporting. For further information on Nature Portfolio policies, see our Editorial Policies and the Editorial Policy Checklist.

Statistics

For all statistical analyses, confirm that the following items are present in the figure legend, table legend, main text, or Methods section.

n/a	Confirmed	
☐	☒	The exact sample size (*n*) for each experimental group/condition, given as a discrete number and unit of measurement
☒	☐	A statement on whether measurements were taken from distinct samples or whether the same sample was measured repeatedly
☐	☒	The statistical test(s) used AND whether they are one- or two-sided<br>*Only common tests should be described solely by name; describe more complex techniques in the Methods section.*
☒	☐	A description of all covariates tested
☒	☐	A description of any assumptions or corrections, such as tests of normality and adjustment for multiple comparisons
☐	☒	A full description of the statistical parameters including central tendency (e.g. means) or other basic estimates (e.g. regression coefficient) AND variation (e.g. standard deviation) or associated estimates of uncertainty (e.g. confidence intervals)
☐	☒	For null hypothesis testing, the test statistic (e.g. *F*, *t*, *r*) with confidence intervals, effect sizes, degrees of freedom and *P* value noted<br>*Give P values as exact values whenever suitable.*
☐	☒	For Bayesian analysis, information on the choice of priors and Markov chain Monte Carlo settings
☒	☐	For hierarchical and complex designs, identification of the appropriate level for tests and full reporting of outcomes
☐	☒	Estimates of effect sizes (e.g. Cohen's *d*, Pearson's *r*), indicating how they were calculated

Our web collection on statistics for biologists contains articles on many of the points above.

Software and code

Policy information about availability of computer code

Data collection	No software was used for data collection
Data analysis	The following software was used for data anlysis:<br><br>- SOAPec (v2.03)<br>- SOAPdenovo2 (v2.04-r24154)<br>- ABySS (v1.9.055)<br>- GapCloser (v1.12-r6)<br>- Pilon (v1.22)<br>- LACHESIS (compiled Apr. 19, 2019)<br>- R (v2.6-4 and v3.6.0)<br>- ggplot2 (v3.3.5)<br>- dispRity (v1.7.0)<br>- pheatmap (v1.0.12)<br>- LastZ (v1.04)<br>- BWA (v0.7.2-r351)<br>- bedtools (v2.25.0 and v2.29.2)<br>- MirMachine (v0.1.2)<br>- MirMiner (v1.0)<br>- BUSCO (v5.2.2)<br>- HaMSTR (1.3.2.6)

- MAFFT (v7.402)
- trimAl (v1.2rev59)
- PhyloBayes (v4.1)
- MCMCtree (v4.9j)
- CODEML (v4.9j)
- ProtTest (v3.4.2)
- IQ-TREE (v1.6.3, v1.6.12 and v2.1.3)
- OrthoFinder (v2.3.5)
- ModelFinder (part of IQ-TREE package v1.6.3)
- Tracer (1.7.1)
- ALE (v1.0)
- CONSEL (v0.2.0)
- DIAMOND (v0.9.30.131)
- MCL (v1:14-137+ds-4)
- PANTHER GO (v15.0 and v17.0)
- BLAST package (v2.6.0+ and v2.10.1+)
- MEGA7 (v7.0.18)
- MEGAX (v10.2.4)
- Gblocks (v0.91b)
- Astral-III (v5.6.3)
- MUSCLE (v5; and that bundled with MEGAX v10.2.4)
- MrBayes (v3.2.6)
- PRANK (v150803)
- RAxML-ng (v0.9.0)
- ETE Toolkit (v3.1.3)
- bowtie (v2.4.2)
- NGmerge (v0.3)
- Picard (v2.23.8)
- SAMtools (v1.10)
- MACS (v2.2.7.1)
- RSEM (v1.3.1)
- STAR (v2.6.1d)

For manuscripts utilizing custom algorithms or software that are central to the research but not yet described in published literature, software must be made available to editors and reviewers. We strongly encourage code deposition in a community repository (e.g. GitHub). See the Nature Portfolio guidelines for submitting code & software for further information.

Data

Policy information about availability of data

All manuscripts must include a data availability statement. This statement should provide the following information, where applicable:

- Accession codes, unique identifiers, or web links for publicly available datasets
- A description of any restrictions on data availability
- For clinical datasets or third party data, please ensure that the statement adheres to our policy

The Eptatretus burgeri (inshore hagfish) v4.0 genome is available in NCBI GenBank under accession number GCA_900186335.3. Raw genome sequencing data together with adult RNA-seq data have been deposited in the European Nucleotide Archive (ENA) at EMBL-EBI under accession number PRJEB21290. ATAC-seq sequencing data have been deposited in Gene Expression Omnibus (GEO) under the following accession numbers: GSE247552. Supplementary files are available at FigShare (https://figshare.com/projects/Hagfish_Genome_Project/163186). Gene annotation used in this study is available at https://www.ensembl.org/Eptatretus_burgeri. A mirror of the UCSC Genome Browser containing hagfish assembly and annotations is available at http://ucsc.crg.eu/.

Research involving human participants, their data, or biological material

Policy information about studies with human participants or human data. See also policy information about sex, gender (identity/presentation), and sexual orientation and race, ethnicity and racism.

Reporting on sex and gender	N/A
Reporting on race, ethnicity, or other socially relevant groupings	N/A
Population characteristics	N/A
Recruitment	N/A
Ethics oversight	N/A

Note that full information on the approval of the study protocol must also be provided in the manuscript.

Field-specific reporting

Please select the one below that is the best fit for your research. If you are not sure, read the appropriate sections before making your selection.

☒ Life sciences ☐ Behavioural & social sciences ☐ Ecological, evolutionary & environmental sciences

For a reference copy of the document with all sections, see nature.com/documents/nr-reporting-summary-flat.pdf

Life sciences study design

All studies must disclose on these points even when the disclosure is negative.

Sample size	No statistical method was used to predetermine sample size. Our statistical tests involved comparison among gene groups. We have included all analyzable genes in each group. In each group, the sample size was always over 80 genes, providing enough statistical power for conventional Student's t test and two-sided Wilcoxon rank sum test.
Data exclusions	No data was excluded from the analyses.
Replication	Nearly all the findings reported in this study correspond to computational analyses of next generation sequencing data, and we thoroughly describe the methods and provide relevant data and code when necessary to reproduce our findings, including raw seq data (genome sequencing, RNA-seq and ATAC-seq; see Data Availability). ATAC-seq data was obtained from two single embryos (one at each stage) due to the scarcity and difficulty in obtaining hagfish embryonic material (few embryos per year, obtained from a single laboratory in the world - Shigeru Kuratani laboratory at RIKEN, Japan-); in this case, we divided the nuclei into two pools per sample in order to at least provide technical replicates. Regulatory profiling analyses have been performed in two different laboratories (RIKEN, Japan, and Institute of Zoology, China) to confirm reproducibility.
Randomization	This study did not involved experimental groups, thus experiments were not randomized.
Blinding	Investigators were not blinded to allocation during experiments and outcome assessment because this study did not involve comparisons between treatment and control groups. Blinding was thus not applicable to this study.

Reporting for specific materials, systems and methods

We require information from authors about some types of materials, experimental systems and methods used in many studies. Here, indicate whether each material, system or method listed is relevant to your study. If you are not sure if a list item applies to your research, read the appropriate section before selecting a response.

Materials & experimental systems

n/a	Involved in the study	
☒	☐	Antibodies
☒	☐	Eukaryotic cell lines
☒	☐	Palaeontology and archaeology
☐	☒	Animals and other organisms
☒	☐	Clinical data
☒	☐	Dual use research of concern
☒	☐	Plants

Methods

n/a	Involved in the study	
☒	☐	ChIP-seq
☒	☐	Flow cytometry
☒	☐	MRI-based neuroimaging

Animals and other research organisms

Policy information about studies involving animals; ARRIVE guidelines recommended for reporting animal research, and Sex and Gender in Research

Laboratory animals	No laboratory animals were used in this study.
Wild animals	Sexually mature adults (unkown age) of the inshore hagfish, Eptatretus burgeri, were captured from the Japan Sea, off the Shimane coast in Japan, on field trips done on different years (stated in the manuscript). Animals were captured with tradicional hagfish traps in determined spots and brought to the lab at RIKEN, Kobe, Japan, where they were kept. A ratio of 3:1 females to males were kept in cages in the sea in their natural environment and eggs were retrieved from the cages a few months later (animals caputured in August, eggs collected in November). Eggs were brought back to the lab where they were assayed at appropiate stages. Adults in the lab were euthanized with an overdose of MS-222 (Tricaine) with the approval of RIKEN ethical committee since they needed to be dissected for the sampling of tissues of interest for the study (brain, testis, liver, skeletal muscle, gills, heart, intestine, kidney).
Reporting on sex	Genome data was obtained from the testis of two male individuals. This was confirmed dissecting the animal and extracting the testis. For embryonic material, sex was undetermined.

Field-collected samples	Adult hagfishes and eggs are kept in tanks of artificial sea water at 16 C, covered to keep a 24-hour dark cycle.
Ethics oversight	The sampling and experiments were conducted according to the institutional and national (Japan) guidelines for animal ethics, approved by the RIKEN Animal Experiments Committee (approvals H14-25-23 and H14-25-25).

Note that full information on the approval of the study protocol must also be provided in the manuscript.
